# A bioinspired redox-modulating copper(ii)–macrocyclic complex bearing non-steroidal anti-inflammatory drugs with anti-cancer stem cell activity†

**DOI:** 10.1039/d2dt00788f

**Published:** 2022-03-16

**Authors:** Alice Johnson, Linda Iffland-Mühlhaus, Joshua Northcote-Smith, Kuldip Singh, Fabrizio Ortu, Ulf-Peter Apfel, Kogularamanan Suntharalingam

**Affiliations:** a School of Chemistry, University of Leicester Leicester LE1 7RH UK k.suntharalingam@leicester.ac.uk fabrizio.ortu@leicester.ac.uk; b Ruhr-Universität Bochum, Anorganische Chemie I Universitätsstraße 150 44801 Bochum Germany ulf.apfel@rub.de; c Fraunhofer UMSICHT Osterfelder Str. 3 46047 Oberhausen Germany

## Abstract

Copper(ii) coordination compounds have been investigated for their anticancer properties for decades, however, none have reached advanced human clinical trials. The poor translation of copper(ii) complexes from *in vitro* studies to (pre)clinical studies can be attributed to their limited efficacy in animal models, which is largely associated with copper leaching and speciation (in biological fluids). Here we report a biologically stable copper(ii) complex based on the active site of Type I Cu electron transport proteins. The copper(ii) complex 1 comprises of dithiacyclam (with soft and hard donor atoms) and two diclofenac units, a nonsteriodial anti-inflammatory drug (NSAID). Extensive biophysical and electrochemical studies show that the solid state structure of 1 is preserved in solution and that it can access both copper(i) and copper(ii) oxidation states without leaching copper or undergoing speciation (in the presence of a cellular reductant). Cell studies show that 1 kills bulk breast cancer cells and highly resistant breast cancer stem cells (CSCs) at micromolar concentrations, and is significantly less toxic towards a panel of non-cancerous cells. Clinically relevant spheroid studies show that 1 is able to inhibit breast CSC-enriched mammosphere formation to a similar extent as salinomycin, a gold standard anti-CSC agent. Mechanistic studies show that 1 evokes breast CSC death by elevating intracellular reactive oxygen species (ROS) and inhibiting cyclooxygenase-2 (COX-2) activity. The former leads to the activation of stress pathways (JNK and p38), which culminates in caspase-dependent apoptosis. This study reinforces the therapeutic potential of copper(ii)–NSAID complexes and provides a bioinspired route to develop stable, ROS-generating copper-based anti-CSC drug candidates.

## Abstract 2

Here we report a biologically stable copper(ii) complex based on the active site of Type I Cu electron transport proteins.

## Introduction

Tumours are inherently heterogeneous, consisting of various subsets of cancer cells with distinct morphological and phenotypic characteristics.^1,2^ Tumours also contain a small proportion of cells with embryonic stem cell-like properties.^3^ These sub-populations, termed cancer stem cells (CSCs), act as drivers for tumour establishment, growth, and spread.^4–6^ CSCs respond differently to therapy compared to the bulk of cancer cells.^7^ CSCs are able to overcome current therapeutic options as their cell cycling profiles tend to mirror slow dividing stem cells rather than rapidly proliferating bulk cancer cells which are the target for most oncological treatments.^8–11^ Once CSCs survive therapy, they are able to reform the primary tumour through asymmetric and symmetric cell division, and produce cells with high metastatic potential that can establish and maintain tumour populations at secondary sites.^12–14^ Therefore, the presence of CSCs within tumours predisposes patients to inferior clinical outcomes.^15^ The positive correlation between CSCs found in tumour biopsies and patient prognosis has prompted intense research activity in the development of therapies that account for tumour heterogeneity, including CSCs.^16^ Current strategies (chemical agents, biologics, and antibody drug conjugates) aimed at removing CSCs are all in the preclinical or clinical trials stage of development, and no method has achieved clinical approval.^17–19^

Several pharmaceutical companies and academic groups have launched programs aimed at targeting functional CSC markers such as components within CSC niches, stem cell-associated signalling pathways, and overexpressed cell surface glycoproteins.^17–19^ A global CSC feature that has been largely overlooked as a therapeutic target is their redox state.^20,21^ CSCs are able to maintain relatively low levels of reactive oxygen species (ROS) compared to bulk cancer cells, and are susceptible to perturbations in their redox state.^20,21^ Others and we have previously shown that small molecules containing metals, capable of generating ROS under physiologically conditions, are able to exploit the finely balanced redox state of CSCs and potently and selectively kill them.^22,23^ Specifically, we have shown that copper(ii)-Schiff base and -polypyridyl complexes bearing one or two nonsteriodial anti-inflammatory drug (NSAID) ligands can kill breast CSCs *in vitro* (in the submicromolar range) by elevating intracellular ROS and inhibiting cyclooxygenase-2 (COX-2).^24–27^ COX-2 is an inflammation-related enzyme that is overexpressed in certain CSCs, and linked to their renewal and proliferation.^28^ Despite the highly promising anti-CSC properties of the copper(ii) complexes *in vitro*, further translation was curtailed due to their limited stability in biologically relevant solutions and their inability to access both copper(ii) and copper(i) oxidation states without undergoing structural reorganisation.^29^

Copper(ii) complexes have been extensively studied as anticancer agents over the last few decades.^30,31^ The mechanism of action of these copper(ii) complexes is highly dependent on the coordinating ligands, however, a significant proportion induce cancer cell death by evoking oxidative stress by way of generating ROS *via* Fenton-type reactions.^30,31^ Other mechanisms of action for copper(ii) complexes include DNA binding, topoisomerase I,II inhibition, and proteasome disruption.^30–33^ A common limitation for copper-based ROS inducers is that they are prone to undergo speciation and leach copper during the redox process required to generate ROS. This arises from the discrepancy in the preferred geometrical preferences of the copper(i) and copper(ii) oxidation states. Copper(i) centres commonly adopt tetrahedral geometries although linear and trigonal planar geometries are possible, while copper(ii) centres prefer square planar, trigonal pyramidal, and octahedral geometries.^34^ The most clinically advanced anticancer copper complexes, called Casiopeinas, are currently in Phase I clinical trials in Mexico.^35,36^ Casiopeinas comprise of copper(ii) bound to bidentate polypyridyl ligands and bidentate α-l-amino acidato or acetylacetone ligands.^37^ Even this promising class of copper agents can suffer from speciation and yield unwanted side effects *in vivo* (such as cardiotoxicity, pulmonary oedema, and inflammation on the peritoneal surface).^38–40^

In order to develop ROS-generating copper(ii) complexes that can kill bulk cancer cells and CSCs, and at the same time remain intact in biological systems, we sought inspiration from nature. Type I Cu electron transport proteins (such as plastocyanin and azurin) undergo efficient redox cycling between the copper(i) and copper(ii) oxidation states without leaching copper owing to hard and soft ligands (*NNSS*-donor set) within their active site.^41–43^ This configuration stabilises both the reduced and oxidised copper forms. Therefore in an attempt to recapitulate the stability conferred by Type I Cu electron transport protein active sites we used dithiacyclam, a flexible two nitrogen-, two sulphur-donor macrocyclic ligand,^44,45^ to prepare ROS-generating copper(ii) agents appended to NSAIDs. Coordination of copper to dithiacyclam *via* the *NNSS*-donor set is known to stabilise both copper(i) and copper(ii) oxidation states, accommodate copper(i) and copper(ii) geometrical preferences, give rise to low reorganisation energy between the two redox states,^46^ and thus envisaged to enable efficient ROS generation and prevent copper leaching or speciation. Herein, we report the synthesis and characterisation of a novel copper(ii)–dithiacyclam complex bound to NSAIDs (and various analogues), and show through detailed electrochemical and biophysical studies that the lead complex is stable and able to redox cycle in solution. Cell-based studies aimed at showcasing the potency of the copper(ii)–dithiacyclam complex toward CSCs (cultured in monolayers and three-dimensional models) and probing intracellular ROS generation and mechanism of action, are also reported.

## Results and discussion

### Synthesis and characterisation of copper(ii)–dithiacyclam and copper(ii)–cyclam complexes bearing NSAIDs

The copper(ii) complexes investigated in this study are depicted in Fig. 1. Copper(ii)–dithiacyclam complexes appended to NSAIDs, diclofenac or naproxen (1 and 2) were synthesised by reacting sodium salts of the NSAIDs with copper(ii) chloride in the presence of dithiacyclam, in methanol or water. The copper(ii)–dithiacyclam complexes 1 and 2 were isolated as indigo or blue solids, respectively, in good yields (66–87%) and characterised by high-resolution mass spectrometry, *infra*-red spectroscopy, and elemental analysis (Fig. S1–S3†). Distinctive molecular ion peaks corresponding to the [M − NSAID]^+^ ion with the appropriate isotopic pattern were observed in the positive mode of the HR ESI-MS spectrum for 1 and 2 (*m*/*z* = 593.0582, [1 − diclofenac]^+^; 526.1383, [2 − naproxen]^+^, Fig. S1 and S2†). The difference between the vibrational stretching frequencies between the asymmetric, *ν*_asym_(CO_2_) and symmetric, *ν*_sym_(CO_2_) carboxylato peaks gives an indication of the binding mode of the associated carboxylic acid group to a given metal centre.^47,48^ According to the IR spectra, the difference (*Δ*) between *ν*_asym_(CO_2_) and *ν*_sym_(CO_2_) stretching bands for 1 and 2 varied between 205–213 cm^−1^ (Fig. S3†), suggestive of a unidentate coordination mode for the carboxylate group on the NSAIDs (diclofenac and naproxen) to the copper(ii) centre (as depicted in Fig. 1). Purity of the bulk solids of 1 and 2 was confirmed by elemental analysis (see ESI†). Indigo crystals of 1 suitable for X-ray diffraction analysis were obtained by slow diffusion of diethyl ether into a concentrated methanolic solution of 1 (CCDC 2130539, Fig. 2A and Table S1†). The complex adopts a distorted octahedral geometry, with copper(ii) bound equatorially to dithiacyclam *via* the *NNSS*-donor set and axially to two diclofenac ligands *via* the carboxylate group. The copper(ii) coordination sphere is consistent with the aforementioned spectroscopic and analytic data for 1. The Cu–N (2.03 Å), Cu–S (2.33 Å) and Cu–O (2.51 Å) bond distances are consistent with bond parameters for related copper(ii) complexes (Table S2†).^44,46,49^

**Fig. 1:**
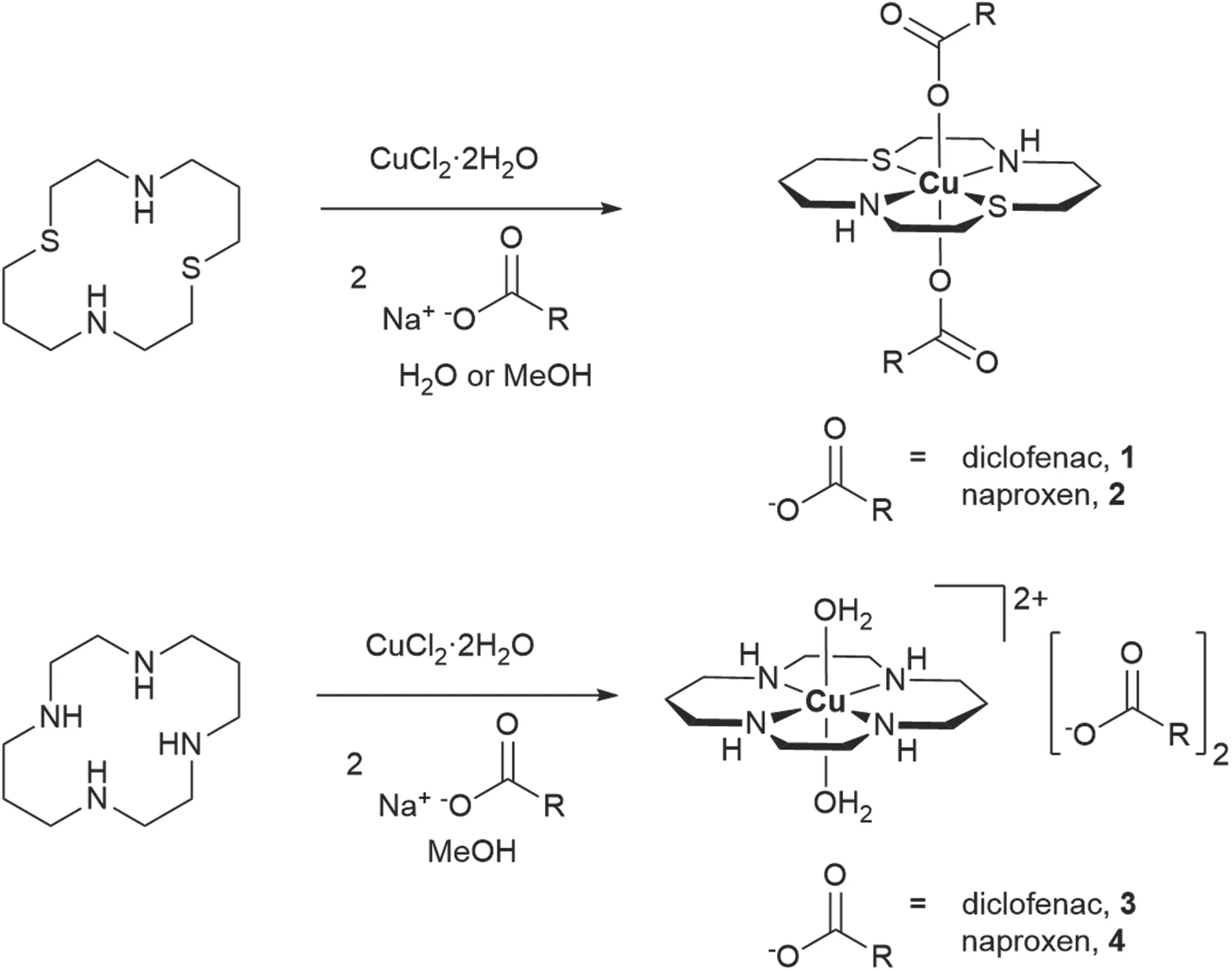
Reaction scheme for the preparation of the copper(ii)–macrocyclic complexes containing diclofenac or naproxen 1–4.

**Fig. 2:**
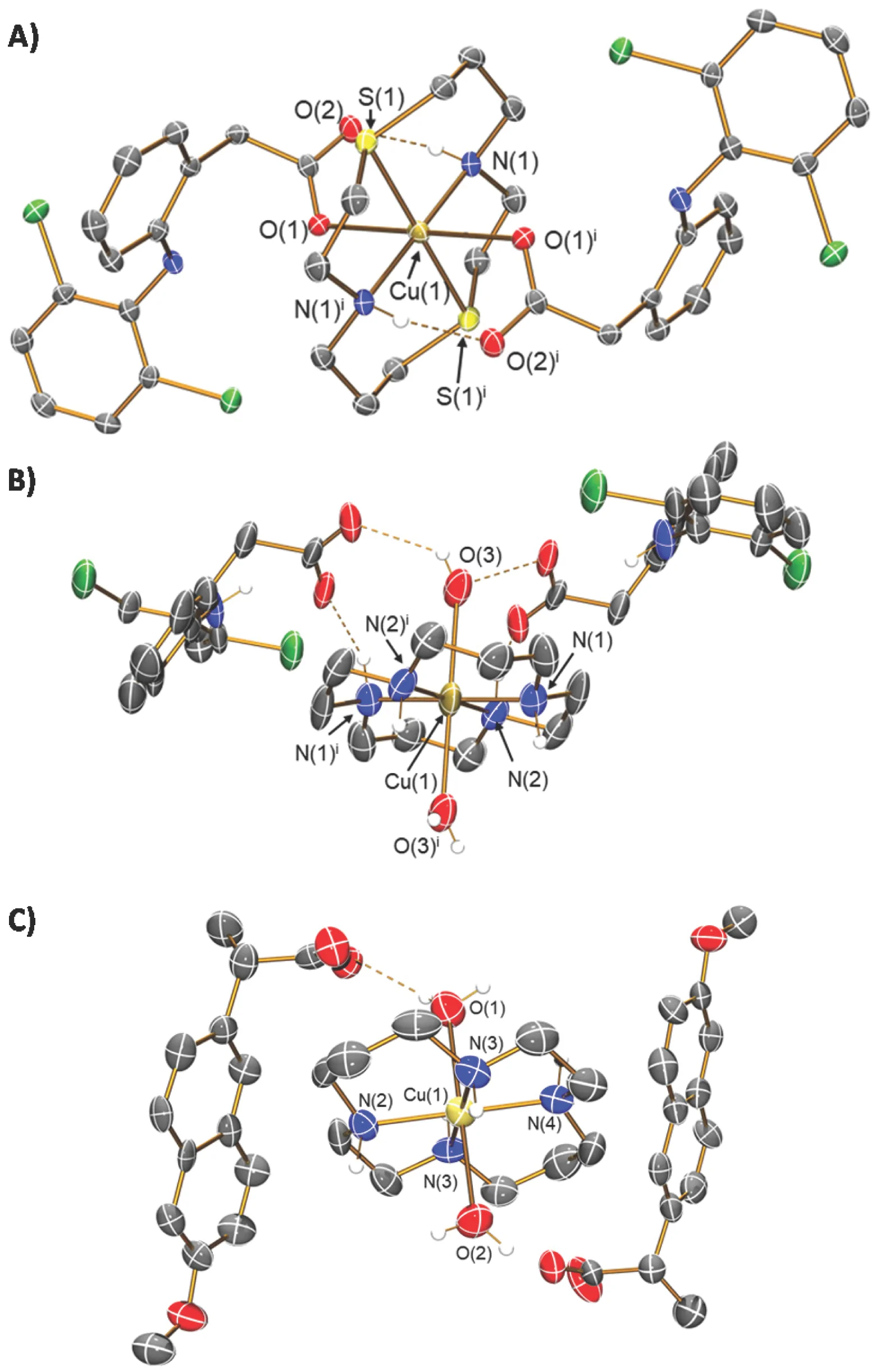
(A) X-ray structure of 1 comprising of a dithiacyclam ligand and two diclofenac ligands; symmetry operation to generate equivalent atoms: *i* = ½ − *x*, ½ − *y*, −*z*. (B) X-ray structure of 3 comprising of a cyclam ligand, two water molecules, and two uncoordinated diclofenac ligands which serve as counter-anions; symmetry operation to generate equivalent atoms: *i* = 1 − *x*, 1 − *y*, 1 − *z*. (C) X-ray structure of 4 comprising of a cyclam ligand, two water molecules, and two uncoordinated naproxen ligands which serve as counter-anions. For (A)–(C), ellipsoids are shown at 50% probability. Cl atoms are shown in green, N in dark blue, C in grey, O in red, S in yellow, and Cu in dark yellow; hydrogen atoms have been excluded for clarity with the exception of those attached to amine functionalities or involved in hydrogen bonding interactions.

The corresponding copper(ii)–cyclam complexes (3 and 4, Fig. 1) were also prepared using the same protocol employed for 1 and 2, with cyclam as the coordinating ligand. The copper(ii)–cyclam complexes 3 and 4 served as control compounds – complexes with *NNNN*-donor sets as opposed to *NNSS*-donor sets. The copper(ii)–cyclam complexes 3 and 4 were isolated as purple solids in reasonable yields (46–53%) and characterised by high-resolution mass spectrometry, *infra*-red spectroscopy, and elemental analysis (see ESI, Fig. S3–S5†). The vibrational stretching frequencies associated to the carboxylato peaks in 3 and 4 appeared in the same region as the corresponding sodium salts of the NSAIDs (Fig. S3†). This suggests that the NSAIDs do not coordinate to the copper(ii) centre and instead remain in their unbound, ionic form. This assumption was confirmed by the X-ray crystal structures of 3 and 4 which revealed a salt, with the cationic component made up of a distorted octahedral copper(ii) centre bound to cyclam *via* the *NNNN*-donor set, and two water molecules (CCDC 2130540–2130541,†Fig. 2B, C and Tables S1, S3, S4†). The NSAIDs (diclofenac or naproxen) remained unbound and served as counter anions, consistent with the IR data. Collectively, the X-ray crystal structures of 1, 3, and 4 show that, within this series of compounds, dithiacyclam promotes coordination of NSAIDs to the copper(ii) centre, whereas cyclam does not. Copper(ii)–macrocyclic complexes without bound or unbound NSAIDs, Cu(dithiacyclam)(NO_3_)_2_ (5) and Cu(cyclam)(NO_3_)_2_ (6), were also prepared and used as additional control compounds. The synthetic protocol and characterisation of 5 and 6 are reported in the Experimental Details section in the ESI (Fig. S6 and S7†).

### Structural integrity and redox-cycling properties in solution

Powder X-ray diffraction studies were performed to confirm that the structures obtained from the single crystal X-ray diffraction analysis of 1 and 3 (Fig. 2A and B) were representative of the bulk solid. For both complexes 1 and 3, the experimental powder diffraction pattern matched the simulated pattern (derived from the single crystal X-ray diffraction data), with the high intensity peaks matching the corresponding 2*θ* positions (Fig. S8 and S9†). This suggested the structures identified from the single crystal X-ray diffraction analysis of 1 and 3 were indeed representative of the bulk samples. This result is also consistent with the elemental analysis of the bulk solids (see ESI†). Having established the structures of 1 and 3 in the corresponding bulk samples, we investigated whether these structures were preserved in solution by comparing the UV-vis spectra of 1 and 3 in solid state and in solution, H_2_O : DMSO (10 : 1). The solid state UV-vis spectra of 1 and 3 showed the same absorption pattern as their spectra in solution (1 mM) (Fig. S10 and S11†). Notably, the broad d–d transition band observed in the solid state UV-vis spectra of 1 (*ca.* 550 nm) and 3 (*ca.* 515 nm) was retained in their solution state spectra, suggesting that the copper(ii) coordination environment present in 1 and 3 was similar in both.

Time course ESI mass spectrometry and UV-vis spectroscopy studies were carried out to further assess the stability of 1 and 3 in solution. In H_2_O : DMSO (10 : 1), the ESI mass spectra (positive mode) of 1 and 3 (500 μM) exhibited distinctive peaks corresponding to the intact complexes, with the expected isotopic pattern, throughout the course of 72 h at 37 °C (*m*/*z* = 593, [1 − diclofenac]^+^; 559, [3 − 2H_2_O − diclofenac]^+^), indicative of solution stability (Fig. S12 and S13†). In PBS : DMSO (200 : 1) and mammary epithelial cell growth medium (MEGM):DMSO (200 : 1) the absorbance and wavelength of the d–d transition band associated to 1 and 3 remained largely unchanged over the course of 24 h at 37 °C (Fig. S14–S17†), implying that no change in the copper(ii) coordination environment occurred under these conditions. Taken together, the ESI mass spectrometry and UV-vis spectroscopy studies show that the structures of 1 and 3 are preserved in solutions relevant for biological studies.

Having confirmed the structural integrity of 1 and 3 in solution, we set out to determine if the copper(ii) complexes could redox cycle in solution. To this aim, cyclic voltammetry and UV-vis absorption spectroelectrochemistry studies were carried out. In DMSO, 1 (1 mM) displayed quasi-reversible redox behaviour between the copper(ii) and copper(i) states, with reduction at −0.14 V *vs.* Ag and corresponding oxidation of copper(i) to copper(ii) at 0.15 V *vs.* Ag (*E*_1/2_(Cu^I/II^) = −0.005 V *vs.* Ag) (Fig. 3A). In H_2_O : DMSO (10 : 1), the cyclic voltammogram of 1 displayed three reduction signals for copper(ii) to copper(i) at potentials of −0.21, −0.41 and −0.57 V *vs.* Ag implicating that three very similar, but electronically slightly different copper(ii) species are present due to solvent effects (Fig. 3A). A similar phenomenon was previously reported for a related nickel(ii)–dithiacyclam complex.^50^ For the corresponding copper(i) to copper(ii) oxidation one peak at 0.13 V *vs.* Ag is observed, leading to the assumption that after reduction of the initial copper(ii) species the same copper(i) species is formed (Fig. 3A). Under identical conditions, for 3 copper(ii) to copper(i) reduction at −0.90 V *vs.* Ag proved to be irreversible in DMSO, while in H_2_O : DMSO (10 : 1) the same reduction is not evident, and probably occurs at a potential outside of the potential window of the solvent system (<−0.8 V *vs.* Ag) (Fig. 3B). As expected the reduction of 1 occurs at significantly more positive potential than 3, owing to the coordination of soft sulphur atoms on the dithiacyclam ligand, which stabilises the reduced copper(i) state.^46,51^ The reduction potential of 1 is within the biological range, and thus 1 is likely to undergo reduction in biologically relevant solutions and inside cells, whereas the reduction of 3 occurs outside this window. UV-Vis absorption spectroelectrochemistry studies in DMSO showed that when a potential of −0.2 V *vs.* Ag (matching the reduction potential) was applied to 1, the corresponding d–d transition band disappeared, indicative of reduction from the copper(ii) to the copper(i) form (Fig. 3C and S18, S19†). Application of the corresponding positive potential to the reduced sample of 1 led to the reappearance of the d–d transition band suggesting oxidation to the copper(ii) form (Fig. 3D and S18, S19†), and moreover showed that 1 could redox cycle in solution. Similar observations were made for 1 in H_2_O : DMSO (10 : 1) showing that 1 is able to undergo a reversible copper(ii)/copper(i) redox process in partially aqueous solution (Fig. S20–S22†). For 3, the corresponding d–d transition band markedly red shifted upon applying a potential of −0.8 V *vs.* Ag (matching the copper(ii)/copper(i) reduction potential) in DMSO (Fig. S23–S25†). Application of a more positive potential to the same sample of 3 produced no further spectral changes (Fig. S23–S25†). This suggests that the reduced form of 3 does not re-oxidise, and thus 3 is unable to redox cycle in solution. Since in H_2_O : DMSO (10 : 1) the potential for the copper(ii)/copper(i) reduction of 3 was not evident within the solvent potential window, UV-vis absorption spectroelectrochemistry studies unsurprisingly resulted in no spectral changes (Fig. S26–S28†).

**Fig. 3:**
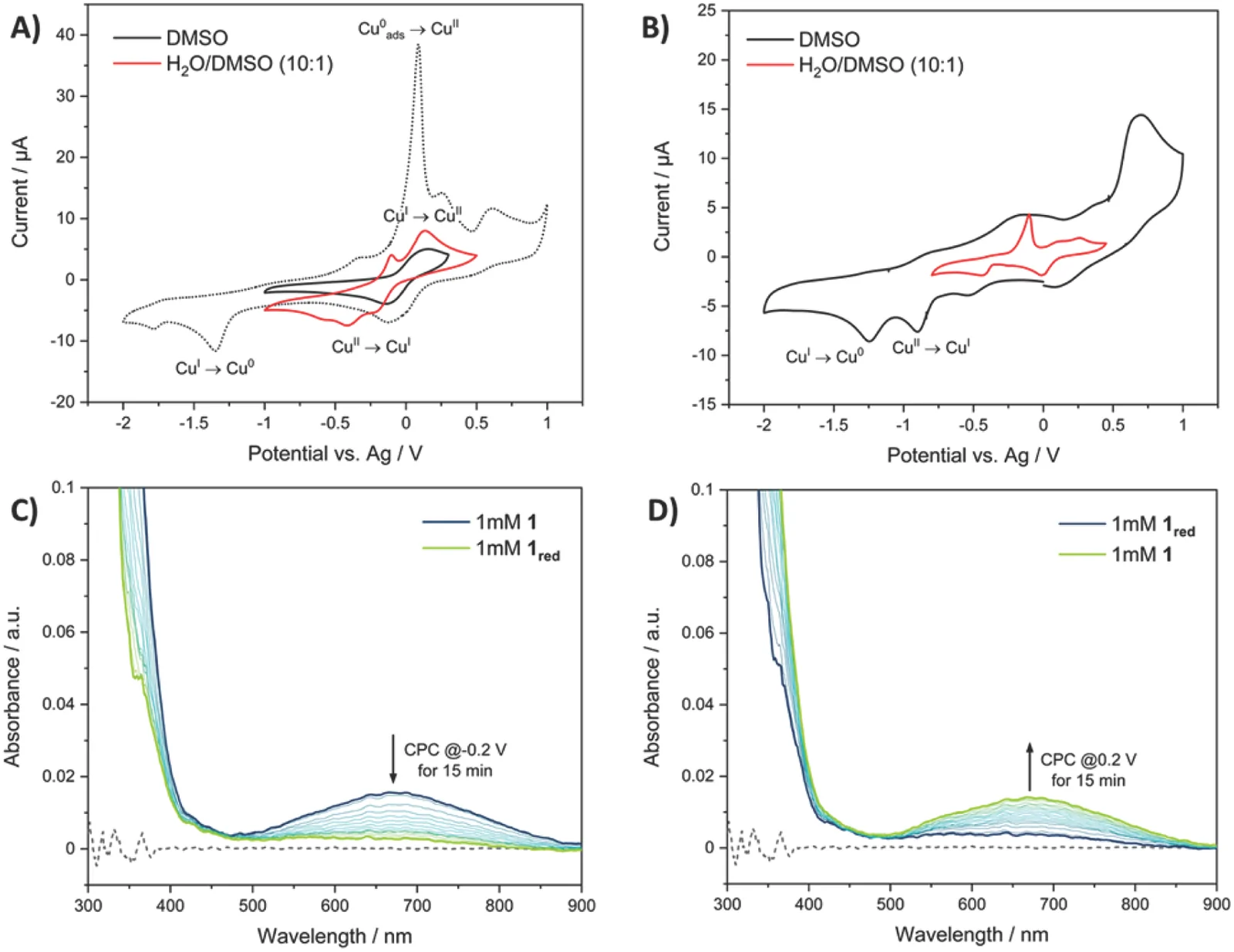
(A) Cyclic voltammograms of 1 (1 mM) in DMSO (black) and in H_2_O : DMSO (10 : 1) (red) with a scan rate of 100 mV s^−1^. Supporting electrolyte: TBAPF_6_ (0.1 M, DMSO) and KCl (0.1 M, H_2_O : DMSO (10 : 1)). (B) Cyclic voltammograms of 3 (1 mM) in DMSO (black) and in H_2_O : DMSO (10 : 1) (red) with a scan rate of 100 mV s^−1^. Supporting electrolyte: TBAPF_6_ (0.1 M, DMSO) and KCl (0.1 M, H_2_O : DMSO (10 : 1)). (C) UV-vis spectra recorded during the electrolysis of 1 (1 mM) in DMSO at a potential of −0.2 V *vs.* Ag (1 min intervals over 15 min). (D) UV-vis spectra of the same solution in (C) after a potential of 0.2 V *vs.* Ag was applied (1 min intervals over 15 min).

Once we recognised the ability of 1 to undergo reversible reduction in solution, we studied the ability of 1 to undergo reduction by glutathione (a cellular reductant) and subsequent oxidation by air. UV-vis spectroscopy studies showed that upon incubation of 1 (1 mM) with glutathione (1 equivalent) in DMSO at 37 °C, the d–d transition band associated to the copper(ii) centre faded rapidly within minutes implicative of reduction to copper(i) (Fig. S29†). Exposure of the same sample to air for 72 h led to the reappearance of the d–d transition band suggesting oxidation to the copper(ii) form (Fig. S29†). Addition of a further equivalences of glutathione led to the disappearance of the d–d transition band (Fig. S29†). Collectively, this shows that 1 is able to undergo reduction by glutathione and subsequent oxidation by air. The ESI mass spectrum of the sample after treatment with glutathione/air exhibited a distinctive peak corresponding to [1 − 2diclofenac]^+^ (*m*/*z* = 297) with the expected isotopic pattern, implying that the copper is retained within the dithiacyclam macrocycle and not lost in solution during this process (Fig. S30†).

### Cancer stem cell and bulk cancer cell potency

The cytotoxicity of the copper(ii) complexes against bulk breast cancer cells (HMLER) and breast CSC-enriched cells (HMLER-shEcad), grown in monolayer cultures, was assessed using the colorimetric MTT assay. The associated IC_50_ values, determined by plotting dose–response curves (Fig. S31 and S32†), are shown in Table 1. The diclofenac-bearing copper(ii) complexes 1 and 3 displayed micromolar toxicity toward both cells, in the same concentration range as salinomycin (an established anti-breast CSC agent).^24^ Of note, 1 and 3 exhibited significantly greater potency (*p* < 0.05, *n* = 18) for HMLER-shEcad cells than HMLER cells with a similar toxicity differential (the concentration difference between the IC_50_ values for HMLER and HMLER-shEcad cells) as salinomycin. The toxicity differential for salinomycin is 7.20 μM whereas the differential for 1 and 3 is 8.1 μM and 15.1 μM, respectively, therefore 1 and 3 theoretically have a similar concentration window to treat CSCs over bulk cancer cells as salinomycin. The naproxen-containing complexes 2 and 4 were non-toxic (IC_50_ > 100 μM) toward both HMLER and HMLER-shEcad cells, suggesting that the NSAID component influences potency (Fig. S33 and S34†). It is worth noting that the cytotoxicity of 1 towards HMLER and HMLER-shEcad cells was significantly higher (*p* < 0.05, *n* = 18) than 3, implying that the nature of the macrocyclic component also plays a determinant role in potency.

**Table 1:** IC_50_ values (μM) of 1, 3, Na diclofenac, 1 : 2 mixture of 5 or 6 with Na diclofenac, and salinomycin against HMLER and HMLER-shEcad cells determined after 72 h incubation (mean of three independent experiments ± SD)

Compound	HMLER IC_50_/μM	HMLER-shEcad IC_50_/μM
1	20.7 ± 0.2	12.5 ± 0.4
3	34.6 ± 3.0	19.5 ± 0.2
Na diclofenac	55.6 ± 0.9	54.0 ± 4.2
5 + Na diclofenac	46.8 ± 3.6	38.4 ± 3.7
6 + Na diclofenac	56.9 ± 3.2	33.1 ± 2.5
Salinomycin^a^	11.4 ± 0.4	4.2 ± 0.4

a Taken from ref. 24.

Control studies showed that the macrocyclic ligands, dithiacyclam and cyclam were non-toxic (IC_50_ > 100 μM) toward HMLER and HMLER-shEcad cells, while sodium diclofenac displayed up to 2.7- and 4.3-fold lower potency compared to 1 and 3 against HMLER or HMLER-shEcad cells (Fig. S35–S37† and Table 1). Cu(dithiacyclam)(NO_3_)_2_ (5) and Cu(cyclam)(NO_3_)_2_ (6) were non-toxic (IC_50_ > 100 μM) toward HMLER and HMLER-shEcad cells (Fig. S38 and S39†). This suggests that the diclofenac component in 1 and 3 is required for potency and the copper(ii)–macrocyclic unit alone is not sufficient to induce cell death (at micromolar concentrations). Upon treatment of 1 : 2 mixtures of sodium diclofenac and either 5 or 6, a 2.3–2.6-fold decrease in potency towards CSC-enriched HMLER-shEcad cells was observed compared to 1 and 3 (Fig. S40, S41† and Table 1). This demonstrates that the preformed complexes 1 and 3 are significantly (*p* < 0.05) better at killing CSCs than a mixture of its individual components. Further toxicity studies with epithelial breast MCF10A cells (IC_50_ = 92.2 ± 1.2 μM), bronchial epithelium BEAS-2B cells (IC_50_ = 57.1 ± 0.9 μM), and embryonic kidney HEK 293 cells (IC_50_ = 57.2 ± 1.0 μM) showed that the most effective copper(ii) complex 1 was significantly (*p* < 0.05, up to 7.4-fold) less toxic towards non-cancerous cells than HMLER or HMLER-shEcad cells (Fig. S42†).

When grown under serum-free, low-attachment conditions, HMLER-shEcad cells can form three-dimensional spheroids called mammospheres. These structures provide a more representative model of tumours than monolayer cell cultures. The inhibitory effect of 1 and 3 on the formation of HMLER-shEcad mammospheres was probed using an inverted microscope. Addition of 1 and 3 at 2 μM (for 5 days) to single cell suspensions of HMLER-shEcad cells led to a significant decrease in the number of mammospheres formed (39–40%) compared to the untreated control (Fig. 4A). Addition of salinomycin at 2 μM (for 5 days) led to an 78% decrease in the number of mammospheres formed (Fig. 4A). Despite 1 and 3 displaying a lower mammosphere inhibitory effect than salinomycin, 1 and 3 (at 2 μM for 5 days) were able to reduce the size of mammospheres formed to a similar extent as salinomycin when compared to the untreated control (Fig. 4B).

**Fig. 4:**
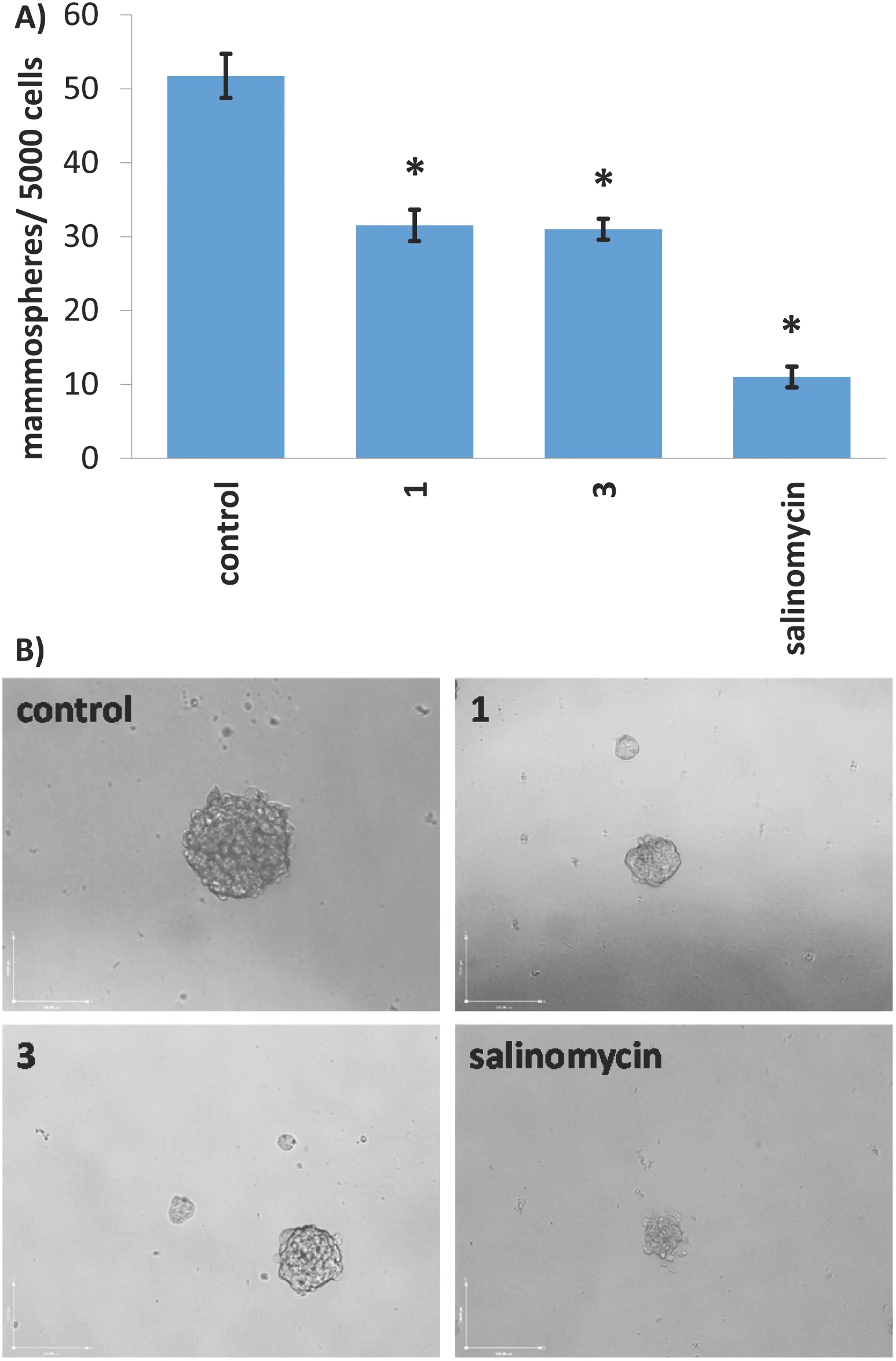
(A) Quantification of mammosphere formation with HMLER-shEcad cells untreated and treated with 1, 3, or salinomycin at 2 μM for 5 days. Student *t*-test, * = *p* < 0.05. (B) Representative bright-field images (×10) of the mammospheres in the absence and presence of 1, 3, or salinomycin at 2 μM for 5 days.

### Cellular uptake and localisation in cancer stem cells

The hydrophobicity of a given small molecule is a predictor of their capacity to be taken up by cells. The lipophilicity of 1 and 3 was determined by measuring the extent to which it partitioned between octanol and water, *P*. The experimentally determined Log *P* values for 1 and 3 were −0.21 ± 0.03 and −0.15 ± 0.01, respectively. The Log *P* values of 1 and 3 are indicative of amphiphilicity, and thus suggests both complexes should be partially soluble in aqueous solutions and readily taken up by cells.

Cellular uptake studies were carried out to determine the CSC permeability of 1 and 3. HMLER-shEcad cells were incubated with 1 (10 μM for 24 h) or 3 (10 μM for 24 h) and the intracellular copper content was determined by inductively coupled plasma mass spectrometry (ICP-MS). Both copper(ii) complexes, 1 and 3 (37.5 ± 0.2 and 27.7 ± 2.0 ng of Cu per million cells, respectively) were readily internalised by HMLER-shEcad cells (Fig. 5). The similar level of HMLER-shEcad cell uptake of 1 and 3 means that cell uptake alone cannot account for the variance in cytotoxicity of 1 and 3 (Table 1). The cellular uptake data also showed that the structural differences between 1 and 3 do not markedly affect CSC uptake. Fractionation studies with HMLER-shEcad cells treated with 1 (10 μM for 24 h) or 3 (10 μM for 24 h) indicated that the majority of internalised 1 and 3 (61–69%, Fig. 5) was detected in the cytoplasm, with only 11–14% (Fig. 5) found in the nucleus. The remainder of 1 and 3 that was taken up by HMLER-shEcad cells was observed in the cell membrane fraction. This suggests that 1 and 3 are likely to induce their cytotoxic effect through changes in the cytosol rather than interactions with nucleus-housed or membrane-bound biomolecules.

**Fig. 5:**
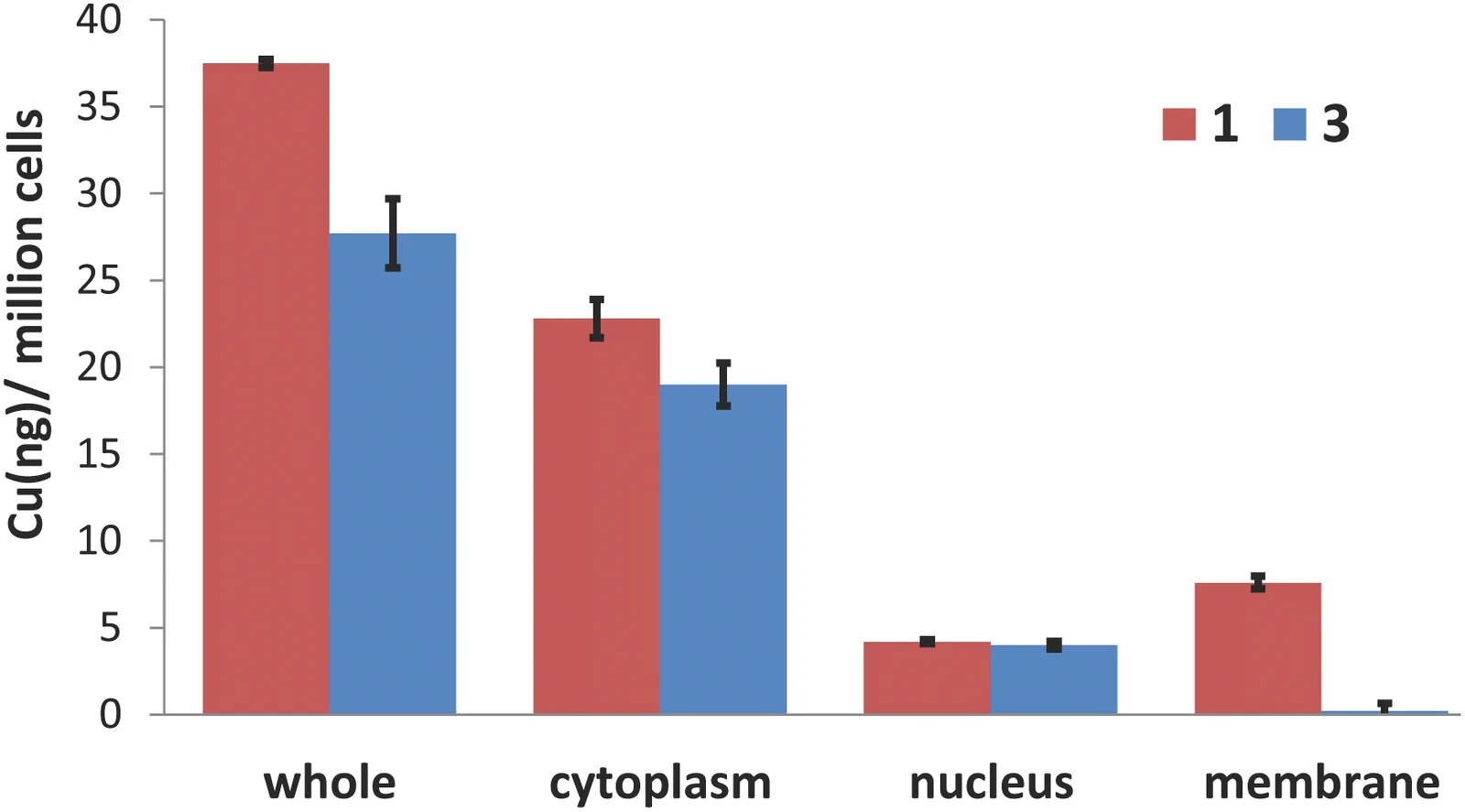
Copper content (ng of Cu/10^6^ cells) in various cellular components upon treatment of HMLER-shEcad cells with 1 or 3 (5 μM for 24 h).

### ROS-generating ability and downstream effects in cancer stem cells

Given that 1 displayed the highest potency towards CSCs and is able to access both copper(ii) and copper(i) oxidation states in solution without leaching copper or undergoing speciation, we investigated whether 1 could elevate intracellular ROS in CSCs. Intracellular ROS levels were determined, over the course of 24 h by flow cytometry, using dichloro-dihydro-fluorescein diacetate (DCFH-DA), a well-established ROS indicator. HMLER-shEcad cells treated with 1 (IC_50_ value × 2) displayed a steady increase in ROS levels, from 3 h exposure to 16 h exposure (29 to 100% increase, *p* < 0.05), relative to untreated control cells (Fig. 6A–G). Shorter (0.5–1 h) or prolonged (24 h) exposure of 1 led to statistically insignificant increases in ROS levels (*p* > 0.05). Notably, for HMLER-shEcad cells treated with 1, ROS levels peaked at 16 h exposure (Fig. 6A–G). The ROS generation profile of 1 is atypical, as most CSC-potent copper(ii) complexes reported thus far induce a short ROS burst within the first few hours of exposure.^25,52^ The difference in the ROS generation profiles could be related to the varying stability thresholds of the copper(ii) complexes in solution. The copper(ii) complex 1 is able to access copper(i/ii) oxidation states without leaching copper, and thus can potentially generate ROS over relatively long periods of time. Previously reported CSC–potent copper(ii) complexes on the other hand, tend to disassemble upon reduction leading to copper release or structural changes,^29,52^ which can evoke uncontrolled redox perturbations over short periods of time. HMLER-shEcad cells treated with 3 (IC_50_ value × 2) displayed a statistically significant increase in ROS levels after 6 h exposure (80% increase, *p* < 0.05) (Fig. S43†). Shorter (0.5–3 h) or longer (16–24 h) exposure of 3 led to statistically insignificant increases in ROS levels (*p* > 0.05). The extent of ROS increase by 3 at its optimal exposure time was lower than that induced by 1 (80% increase after 6 h for 3 and 100% increase after 16 h for 1). This is consistent with the ability of 1 to redox cycle in solution and the inability of 3 to do likewise. As expected, HMLER-shEcad cells treated with H_2_O_2_ (150 μM) exhibited a significant increase (*p* < 0.05) in ROS levels after short (0.5–6 h, up to 13.5-fold) and long (16–24 h, up to 5-fold) exposure times, compared to untreated control cells (Fig. S44†). Independent cell viability studies in the presence of *N*-acetylcysteine (2.5 mM), a ROS scavenger, showed that the potency of 1 towards HMLER-shEcad cells decreased significantly (IC_50_ value increased from 12.5 ± 0.4 μM to 16.1 ± 0.7 μM, *p* < 0.05) (Fig. S45†). This indicates that 1-mediated CSC death could be related to intracellular ROS generation.

**Fig. 6:**
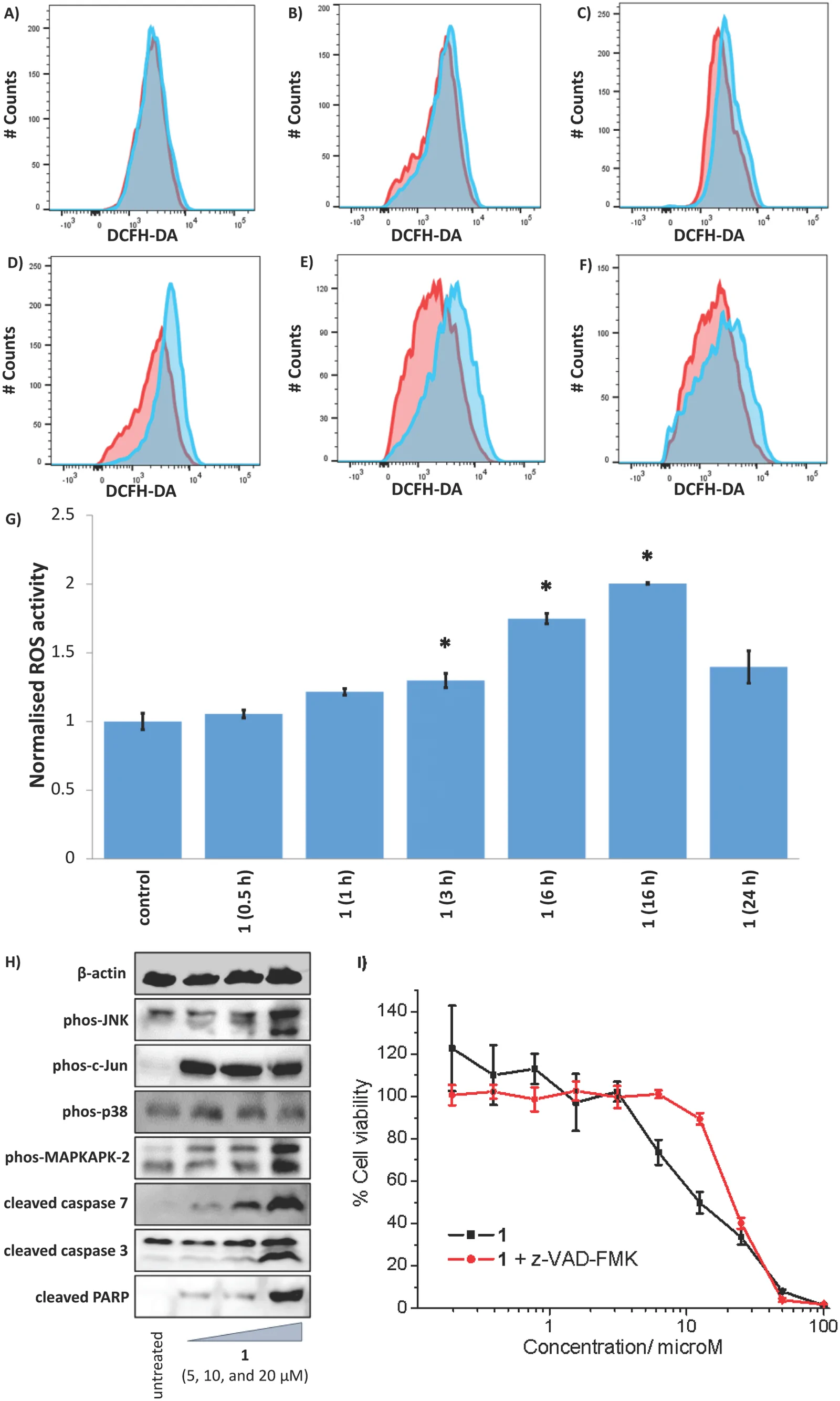
Representative histograms displaying the green fluorescence emitted by DCFH-DA-stained HMLER-shEcad cells (red) and HMLER-shEcad cells treated with 1 (IC_50_ value × 2) (blue) for (A) 30 min, (B) 1 h, (C) 3 h, (D) 6 h, (E) 16 h, and (F) 24 h. (G) Summarised ROS data. Normalised ROS activity in untreated HMLER-shEcad cells (control) and HMLER-shEcad cells treated with 1 (IC_50_ value × 2 for 0.5, 1, 3, 6, 16, and 24 h). (H) Immunoblotting analysis of proteins related to the JNK, p38, and apoptosis pathways. Protein expression in HMLER-shEcad cells following treatment with 1 (5, 10, and 20 μM for 72 h) (I) Representative dose–response curves of 1 against HMLER-shEcad cells in the absence and presence of z-VAD-FMK (5 μM) after 72 h incubation.

The elevation of intracellular ROS levels can activate stress pathways such as the Jun-amino-terminal kinase (JNK) and/or p38 MAP kinase (MAPK) pathways.^53^ Therefore, we carried out immunoblotting studies to monitor changes in the expression of biomarkers related to these pathways. HMLER-shEcad cells treated with 1 (5–20 μM for 72 h) displayed enhanced phosphorylation of JNK and p38 and their respective downstream effectors, c-Jun and MAP kinase-activated protein kinase 2 (MAPKAPK-2) (Fig. 6H). The activation of JNK and p38 pathways can induce cell death *via* apoptosis.^54^ HMLER-shEcad cells incubated with 1 (5–20 μM for 72 h) expressed higher levels of cleaved caspase 3, caspase 7, and poly-ADP ribose polymerase (PARP) relative to untreated cells (Fig. 6H), suggestive of caspase-dependent apoptosis. Furthermore, upon co-treatment of 1 and z-VAD-FMK (5 μM), the potency of 1 towards HMLER-shEcad cells decreased significantly (IC_50_ value increased from 12.5 ± 0.4 μM to 21.7 ± 0.4 μM, *p* < 0.05) (Fig. 6I). This further proves that 1 induces caspase-dependent apoptosis in CSCs. Collectively the flow cytometry, immunoblotting, and cytotoxicity studies shows that 1-induced ROS production promotes JNK and p38 pathway activation and apoptosis.

### COX-2 dependent cancer stem cell death

COX-2 is highly expressed in certain CSCs and has been linked to CSC regulation and preservation.^28^ The expression of COX-2 in CSCs is also positively correlated to their ability to resist chemotherapeutic and radiotherapeutic interventions.^55^ Given that the copper(ii) complex 1 comprises of two diclofenac moieties, we carried out flow cytometry studies to determine if the cytotoxic mechanism of action of 1 was related to changes in COX-2 expression. HMLER-shEcad cells were pre-treated with lipopolysaccharide (LPS) (2.5 μg L^−1^ for 24 h), to increase basal COX-2 levels, and then treated with 1 (IC_50_ value) or diclofenac (10–40 μM) for 48 h. COX-2 expression was then determined by flow cytometry. A noticeable decrease in COX-2 expression was observed in HMLER-shEcad cells treated with either 1 or diclofenac compared to untreated cells (Fig. S46 and S47†). This suggests that the cytotoxic mechanism of action of 1 may involve COX-2 downregulation. To probe this further, cytotoxicity studies were carried out with HMLER-shEcad cells in the presence of prostaglandin E2 (PGE2) (20 μM, 72 h), the functional product of COX-2-catalysed arachidonic acid metabolism. The IC_50_ value for 1 against HMLER-shEcad cells in the presence of PGE2 was significantly (*p* < 0.05, 2.2-fold, IC_50_ value = 28.1 ± 0.4 μM) higher than for 1 alone (Fig. S48†), implying that 1 induces COX-2-dependent CSC death.

## Conclusions

In summary we report the synthesis, characterisation, redox properties, and anti-CSC activity of a novel copper(ii)–dithiacyclam complex 1 containing two diclofenac moieties. Single crystal X-ray analysis showed that 1 consists of copper(ii) bound to the *NNSS*-donor set within dithiacyclam and to the carboxylate group in two independent diclofenac ligands. The coordination environment of the copper(ii) centre in 1 resembles that of the active sites of Type I Cu electron transport proteins (such as plastocyanin and azurin), and thus was expected to accommodate both copper(ii) and copper(i) oxidation states. Solid and solution state UV-vis spectroscopy studies showed that the structure of 1 was retained in both states. Time course ESI mass spectrometry and UV-vis spectroscopy studies showed that the structure of 1 was preserved in solutions relevant for biological studies (including cell media) over the course of several days. Cyclic voltammetry and UV-vis absorption spectroelectrochemistry studies proved that 1 is capable of undergoing reversible reduction in solution. Furthermore, 1 was shown to undergo reduction by glutathione and subsequent oxidation in air without losing copper. Collectively, the biophysical studies showed that 1 could potentially overcome copper leaching and speciation in solution, a common limitation of many copper-based anticancer agents. Monolayer cell studies showed that 1 was able to kill CSC-enriched HMLER-shEcad cells over CSC-depleted HMLER cells, with a similar toxicity differential to salinomycin, a clinically-tested potassium ionophore known to selectively kill CSCs. Spheroid studies (carried out with three-dimensional cultures) showed that 1 reduced the formation and size of mammospheres to a similar extent as salinomycin. Mechanistic studies revealed that 1 induced its CSC cytotoxic effect by generating intracellular ROS and inhibiting COX-2. The mechanism was consistent with the structural design of 1. The elevation of ROS by 1 in CSCs leads to JNK and p38 pathway activation and caspase-dependent apoptosis. Our findings show that biologically stable copper(ii) complexes can be prepared with macrocyclic ligands containing soft and hard donor sites, and such complexes can induce CSC death by elevating intracellular ROS levels without leaching copper. More generally, our studies could pave the way for the development of other bioinspired redox-active copper(ii) complexes for use in CSC-focussed chemotherapy.

## Author contributions

Conceptualization, K.S., A.J., and U.-P.A.; methodology, A.J., L.I-M., J.N.S., K.S., F.O., U.-P.A., and K.S.; formal analysis, A.J., L.I-M., J.N.S., K.S., F.O., U.-P.A., and K.S.; investigation, A.J., L.I-M., J.N.S., K.S., F.O., U.-P.A., and K.S.; writing—original draft preparation, K.S., A.J., L.I-M., and U.-P.A.; writing—review and editing, A.J., L.I-M., J.N.S., K.S., F.O., U.-P.A., and K.S.; supervision, K.S., F.O., and U.-P.A.; project administration, K.S., F.O., and U.-P.A.; funding acquisition, K.S., F.O., and U.-P.A.

## Conflicts of interest

There are no conflicts to declare.

## Supplementary Material

DT-051-D2DT00788F-s001

DT-051-D2DT00788F-s002
