# Supplementary material for: A bioinspired redox-modulating copper(ii)–macrocyclic complex bearing non-steroidal anti-inflammatory drugs with anti-cancer stem cell activity
Source: Dalton Trans. 2022 Mar 16;51(15):5904–12. doi: 10.1039/d2dt00788f (PMC13762695; doi:10.1039/d2dt00788f)
Supplement: Supplementary file 1 [file DT-051-D2DT00788F-s002.pdf]

Electronic Supplementary Material (ESI) for Dalton Transactions.
This journal is © The Royal Society of Chemistry 2022
Supplementary Information (SI) for Dalton Transactions.
This journal is © The Royal Society of Chemistry

Supporting Information for

A bioinspired redox-modulating copper(II)-macrocyclic complex bearing non-steroidal anti-inflammatory drugs with anti-cancer stem cell activity

Alice Johnson,[a] Linda Iffland,[b] Joshua Northcote-Smith,[a] Kuldip Singh,[a] Fabrizio Ortu,*[a] Ulf-Peter Apfel,*[b,c] and Kogularamanan Suntharalingam*[a]

[a] School of Chemistry, University of Leicester, Leicester, UK

[b] Ruhr-Universität Bochum, Anorganische Chemie I, Universitätsstraße 150, 44801 Bochum, Germany

[c] Fraunhofer UMSICHT, Osterfelder Str. 3, 46047 Oberhausen, Germany

* To whom correspondence should be addressed:
Email: k.suntharalingam@leicester.ac.uk; ulf.apfel@rub.de; fabrizio.ortu@leicester.ac.uk

Table of Content

Experimental Details

Fig. S1 (Top) Theoretical isotope model for [**1**-diclofenac]$^+$ ($C_{24}H_{32}Cl_2CuN_3O_2S_2$) and (bottom) the experimentally determined high-resolution ESI-QTOF mass spectrum for the [**1**-diclofenac]$^+$ ion.

Fig. S2 (Top) Theoretical isotope model for [**2**-naproxen]$^+$ ($C_{24}H_{35}CuN_2O_3S_2$) and (bottom) the experimentally determined high-resolution ESI-QTOF mass spectrum for the [**2**-naproxen]$^+$ ion.

Fig. S3 ATR spectrum of (A) **1**, (B) **2**, (C) **3**, (D) **4**, (E) sodium diclofenac, and (F) sodium naproxen in the solid form.

Fig. S4 (Top) Theoretical isotope model for [**3**-2H_2O-diclofenac]$^+$ ($C_{24}H_{34}Cl_2CuN_5O_2$) and (bottom) the experimentally determined high-resolution ESI-QTOF mass spectrum for the [**3**-2H_2O-diclofenac]$^+$ ion.

Fig. S5 (Top) Theoretical isotope model for [**4**-2H_2O-naproxen]$^+$ ($C_{24}H_{37}CuN_4O_3$) and (bottom) the experimentally determined high-resolution ESI-QTOF mass spectrum for the [**4**-2H_2O-naproxen]$^+$ ion.

Fig. S6 ESI mass spectrum (positive mode) for [Cu(1,8-dithia-4,11-diazacyclotetradecane)$(NO_3)_2$], **5** in methanol.

Fig. S7 ESI mass spectrum (positive mode) for [Cu(1,4,8,11-tetraazacyclotetradecane)$(NO_3)_2$], **6** in methanol.

Table S1. Crystallographic data for **1**, **3** and **4**.

Table S2. Selected bond lengths (Å) and angles (°) for **1**.

Table S3. Selected bond lengths (Å) and angles (°) for **3**.
Table S4. Selected bond lengths (Å) and angles (°) for **4**.
Fig. S8 (Top) Experimentally determined powder X-ray diffraction pattern of **1** and (bottom) simulated pattern based on the single crystal X-ray diffraction analysis of **1**.
Fig. S9 (Top) Experimentally determined powder X-ray diffraction pattern of **3** and (bottom) simulated pattern based on the single crystal X-ray diffraction analysis of **3**.
Fig. S10. Solid state UV-vis spectra of **1** and **3**.
Fig. S11 UV-vis spectra of **1** and **3** (1 mM) in H_2O:DMSO (10:1).
Fig. S12 ESI mass spectra (positive mode) of **1** (500 μM) in H_2O:DMSO (10:1) (A) before and after incubation for (B) 24 h, (C) 48 h, and (D) 72 h at 37 ^{o}C.
Fig. S13 ESI mass spectra (positive mode) of **3** (500 μM) in H_2O:DMSO (10:1) (A) before and after incubation for (B) 24 h, (C) 48 h, and (D) 72 h at 37 ^{o}C.
Fig. S14 UV-vis spectra of **1** (1 mM) in PBS:DMSO (200:1) over the course of 24 h.
Fig. S15 UV-vis spectra of **3** (1 mM) in PBS:DMSO (200:1) over the course of 24 h.
Fig. S16 UV-vis spectra of **1** (1 mM) in MEGM:DMSO (200:1) over the course of 24 h.
Fig. S17 UV-vis spectra of **3** (1 mM) in MEGM:DMSO (200:1) over the course of 24 h.
Fig. S18 Cyclic voltammogram of **1** (1 mM) within the SEC-UV-vis set-up in DMSO using $TBAPF_6$ (0.1 M) as the supporting electrolyte.
Fig. S19 Controlled potential coulometry of **1** (1 mM) in DMSO with $TBAPF_6$ (0.1 M) for 15 min at -0.2 V vs. Ag (A) and 0.2 V vs. Ag (B) within the SEC-UV-vis set-up.
Fig. S20 Cyclic voltammogram of **1** (1 mM) within the SEC-UV-vis set-up in H_2O:DMSO (10:1) using KCl (0.1 M) as the supporting electrolyte.
Fig. S21 Controlled potential coulometry of **1** (1 mM) in H_2O:DMSO (10:1) with KCl (0.1 M) for 10 min at -0.2 V vs. Ag (A) and 0.5 V vs. Ag (B) within the SEC-UV-vis set-up.
Fig. S22 UV-vis spectra recorded during the electrolysis of **1** (1 mM) in H_2O:DMSO (10:1) at a potential of -0.2 V vs. Ag (1 min interval) (A). To the same solution afterwards a potential of 0.5 V vs. Ag was applied, and UV-vis spectra were recorded in 1 min interval over 15 min (B).
Fig. S23 Cyclic voltammogram of **3** (1 mM) within the SEC-UV-vis set-up in DMSO using $TBAPF_6$ (0.1 M) as the supporting electrolyte.
Fig. S24 Controlled potential coulometry of **3** (1 mM) in DMSO with $TBAPF_6$ (0.1 M) for 15 min at -0.8 V vs. Ag (A) and 0 V vs. Ag (B) within the SEC-UV-vis set-up.
Fig. S25 UV-vis spectra recorded during the electrolysis of **3** (1 mM) in DMSO at a potential of -0.8 V vs. Ag (1 min interval) (A). To the same solution afterwards a potential of 0 V vs. Ag was applied, and UV-vis spectra were recorded in 1 min interval over 15 min (B).
Fig. S26 Cyclic voltammogram of **3** (1 mM) within the SEC-UV-vis set-up in H_2O:DMSO (10:1) using KCl (0.1 M) as the supporting electrolyte.
Fig. S27 Controlled potential coulometry of **3** (1 mM) in H_2O:DMSO (10:1) with KCl (0.1 M) for 10 min at -1.0 V vs. Ag within the SEC-UV-vis set-up.
Fig. S28 UV-vis spectra recorded during the electrolysis of **3** (1 mM) in H_2O:DMSO (10:1) at a potential of -1.0 V vs. Ag (1 min interval) over 10 min.

Fig. S29 UV-vis spectra of **1** (1 mM) in DMSO, upon addition of glutathione (1 mM, 1 equivalence), exposure to air for 72 h, and further addition of glutathione (1 mM, 1 equivalence).

Fig. S30 ESI mass spectra (positive mode) of **1** (1 mM) in DMSO after addition of glutathione (1 mM, 1 equivalence), exposure to air for 72 h, and further addition of glutathione (1 mM, 1 equivalence).

Fig. S31 Representative dose-response curves for the treatment of (A) HMLER or (B) HMLER-shEcad cells with **1** after 72 h incubation.

Fig. S32 Representative dose-response curves for the treatment of (A) HMLER or (B) HMLER-shEcad cells with **3** after 72 h incubation.

Fig. S33 Representative dose-response curves for the treatment of (A) HMLER or (B) HMLER-shEcad cells with **2** after 72 h incubation.

Fig. S34 Representative dose-response curves for the treatment of (A) HMLER or (B) HMLER-shEcad cells with **4** after 72 h incubation.

Fig. S35 Representative dose-response curves for the treatment of (A) HMLER or (B) HMLER-shEcad cells with dithiacyclam after 72 h incubation.

Fig. S36 Representative dose-response curves for the treatment of (A) HMLER or (B) HMLER-shEcad cells with cyclam after 72 h incubation.

Fig. S37 Representative dose-response curves for the treatment of (A) HMLER or (B) HMLER-shEcad cells with sodium diclofenac after 72 h incubation.

Fig. S38 Representative dose-response curves for the treatment of (A) HMLER or (B) HMLER-shEcad cells with **5** after 72 h incubation.

Fig. S39 Representative dose-response curves for the treatment of (A) HMLER or (B) HMLER-shEcad cells with **6** after 72 h incubation.

Fig. S40 Representative dose-response curves for the treatment of (A) HMLER or (B) HMLER-shEcad cells with **5** and sodium diclofenac after 72 h incubation.

Fig. S41 Representative dose-response curves for the treatment of (A) HMLER or (B) HMLER-shEcad cells with **6** and sodium diclofenac after 72 h incubation.

Fig. S42 Representative dose-response curves for the treatment of (A) MCF10A, (B) BEAS-2B, or (C) HEK293 cells with **1** after 72 h incubation.

Fig. S43 Normalised ROS activity in untreated HMLER-shEcad cells (control) and HMLER-shEcad cells treated with **3** (IC_{50} value × 2 for 0.5, 1, 3, 6, 16, and 24 h).

Fig. S44 Normalised ROS activity in untreated HMLER-shEcad cells (control) and HMLER-shEcad cells treated with H_2O_2 (150 μM for 0.5, 1, 3, 6, 16, and 24 h).

Fig. S45 Representative dose-response curve of **1** against HMLER-shEcad cells in the presence of *N*-acetylcysteine (2.5 mM) after 72 h incubation.

Fig. S46 Representative histograms displaying the green fluorescence emitted by anti-COX-2 Alexa Fluor 488 nm antibody-stained HMLER-shEcad cells treated with LPS (2.5 μg/ L) for 24 h (red) followed by 48 h in media containing **1** (IC_{50} value, blue).

Fig. S47 Representative histograms displaying the green fluorescence emitted by anti-COX-2 Alexa Fluor 488 nm antibody-stained HMLER-shEcad cells treated with LPS (2.5 μg/ L) for 24 h (red) followed by 48 h in media containing diclofenac (10 μM, blue; 20 μM, orange; and 40 μM, green).

Fig. S48 Representative dose-response curve of **1** against HMLER-shEcad cells in the presence of PGE2 (20 μM) after 72 h incubation.

References

Experimental Details. Instrumentation. Mass spectra were recorded on a Micromass Quattro with the electrospray (ESI) technique and on a Kratos Concept 1H (ESI-TOF). ^1H NMR were recorded at room temperature on a Bruker Avance 400 spectrometer (^1H 400.0 MHz) with chemical shifts (δ, ppm) reported relative to the solvent peaks of the deuterated solvent. ICP-MS were measured using a Thermo Scientific ICAP-Qc quadrupole ICP mass spectrometer. Elemental Analysis was performed commercially at the University of Cambridge. Solid state UV-vis spectra were recorded on a JASCO V-670 spectrophotometer equipped with an integrating sphere and barium sulphate as a background reference. Powder X-ray diffraction measurements of bulk samples were performed on a Bruker Phaser D2 diffractometer equipped with a LynxEye detector operating at 30 kV acceleration voltage and 10 mA emission current using a Cu-K_α radiation source (λ = 1.54184 Å). Based on the corresponding cif data obtained by single crystal X-ray diffraction analysis powder XRD patterns were simulated with Mercury 3.10.2 software using the wavelength of a Cu-K_α radiation source as for the experimental obtained data.

Starting Materials. 1,8-dithia-4-11-diazacyclotetradecane (**L^1**) was prepared according to a previously reported procedure.[1] 1,4,8,11-Tetraazacyclotetradecane, $CuCl_2{\cdot}2H_2O$, $Cu(NO_3)_2{\cdot}3H_2O$, sodium diclofenac, and sodium naproxen were purchased from Sigma-Aldrich and used without further purification. Solvents were purchased from Fisher and used without further purification.

Synthesis of [Cu(1,8-dithia-4,11-diazacyclotetradecane)(diclofenac)$_2$], 1. To a solution of 1,8-dithia-4,11-diazacyclotetradecane (46.9 mg, 0.2 mmol) in H_2O (5 mL) was added $CuCl_2{\cdot}2H_2O$ (34.1 mg, 0.2 mmol) and sodium diclofenac (126.4 mg, 0.4 mmol) and the resultant dark blue solution stirred for 2 h. An indigo precipitate formed which was collected by filtration and vacuum dried overnight to give the product (155.0 mg, 87%); IR (solid, ATR, cm^{-1}): 3388, 3311, 3068, 3039, 2914, 2865, 1569, 1549, 1498, 1466, 1451, 1443, 1364, 1281, 773, 737, 699, 658, 619, 520, 481, 445, 303; HR-ESI-QTOF-MS *m/z*: [**1**-diclofenac]$^+$ Calcd for $C_{24}H_{32}Cl_2CuN_3O_2S_2$: 593.0586; Found 593.0582; Anal. Calcd. for $C_{38}H_{42}Cl_4CuN_4O_4S_2{\cdot}2H_2O$: C 49.38, H 5.02, N 6.06. Found: C 49.28, H 4.93, N 6.15.

Synthesis of [Cu(1,8-dithia-4,11-diazacyclotetradecane)(naproxen)$_2$], 2. To a solution of 1,8-dithia-4,11-diazacyclotetradecane (46.9 mg, 0.2 mmol) in MeOH (10 mL) was added $CuCl_2{\cdot}2H_2O$ (34.1 mg, 0.2 mmol) and sodium naproxen (100.9 mg, 0.4 mmol) and the resultant dark blue solution stirred for 2 h. MeOH was removed *in vacuo* and the resultant residue dissolved in MeCN (10 mL). The solution was filtered through Celite, the dark blue filtrate evaporated to minimum volume and Et_2O (10 mL) added to precipitate a blue solid which was collected by filtration and vacuum dried overnight to give the product (99.9 mg, 66%); IR (solid, ATR, cm^{-1}): 3403, 2933, 2868, 1605, 1564, 1484, 1383, 1351, 1266, 1234, 1214, 1162, 1029, 892, 872, 852, 812, 687, 526, 477, 445, 373, 336; HR-ESI-QTOF-MS *m/z*: [**2**-naproxen]$^+$ Calcd for $C_{24}H_{35}CuN_2O_3S_2$: 526.1385; Found 526.1383; Anal. Calcd. $C_{38}H_{48}CuN_2O_6S_2{\cdot}2H_2O$: C 57.59, H 6.61, N 3.53. Found: C 57.39, H 6.54, N 3.54.

Synthesis of [Cu(1,4,8,11-tetraazacyclotetradecane)(H_2O)$_2$](diclofenac)$_2$, 3. To a solution of 1,4,8,11-tetraazacyclotetradecane (60.1 mg, 0.3 mmol) in MeOH (10 mL) was added $CuCl_2{\cdot}2H_2O$ (51.1 mg, 0.3 mmol) and sodium diclofenac (189.1 mg, 0.6 mmol) and the solution stirred for 2 h. A purple precipitate formed which was collected by filtration, washed with Et_2O (2 x 10 mL) and vacuum dried overnight to give the product (142.5 mg, 53%); IR (solid, ATR, cm^{-1}): 3349, 3171, 2964, 2914, 2866, 1583, 1576, 1555, 1506, 1452, 1369, 1303, 1096, 1067, 1005, 889, 868, 744, 628, 562, 541, 525, 450, 364, 301; HR-ESI-QTOF-

MS *m/z*: [**3**-2H_2O-diclofenac]$^+$ Calcd for $C_{24}H_{34}Cl_2CuN_5O_2$: 559.1364; Found 559.1360; Anal. Calcd. $C_{38}H_{48}Cl_4CuN_6O_6$: C 51.27, H 5.44, N 9.44. Found: C 51.07, H 5.10, N 9.30.

Synthesis of [Cu(1,4,8,11-tetraazacyclotetradecane)(H_2O)$_2$](naproxen)$_2$, 4. To a solution of 1,4,8,11-tetraazacyclotetradecane (60.1 mg, 0.3 mmol) in MeOH (10 mL) was added $CuCl_2{\cdot}2H_2O$ (51.1 mg, 0.3 mmol) and sodium naproxen (151.3 mg, 0.6 mmol) and the solution stirred for 2 h. MeOH was removed *in vacuo* and the purple residue dissolved in MeCN (10 mL). The solution was filtered through Celite, the purple filtrate evaporated to minimum volume and Et_2O (10 mL) added to precipitate a purple solid which was collected by filtration and vacuum dried overnight to give the product (103.8 mg, 46%); IR (solid, ATR, cm^{-1}): 3364, 3230, 3174, 2932, 1590, 1504, 1480, 1463, 1382, 1354, 1265, 1232, 1208, 1159, 1123, 1098, 1062, 1029, 1005, 924, 892, 851, 746, 685, 523, 477, 435, 324, 302; HR-ESI-QTOF-MS *m/z*: [**4**-2H_2O-naproxen]$^+$ Calcd for $C_{24}H_{37}CuN_4O_3$: 492.2162; Found 492.2171; Anal. Calcd. $C_{38}H_{54}CuN_4O_8{\cdot}H_2O$: C 58.78, H 7.27, N 7.22. Found: C 58.86, H 7.08, N 7.26.

Synthesis of [Cu(1,8-dithia-4,11-diazacyclotetradecane)(NO_3)$_2$], 5. To a solution of the 1,8-dithia-4,11-diazacyclotetradecane (46.9 mg, 0.2 mmol) in acetone (5 mL) was added $Cu(NO_3)_2{\cdot}3H_2O$ (48.3 mg, 0.2 mmol) and the solution stirred for 1 h. The solvent was evaporated to minimum volume and Et_2O (10 mL) added. The resultant precipitate was collected and vacuum dried overnight to give the product as a blue solid (58.3 mg, 69%); ESI-MS *m/z*: [M-2(NO_3)]$^+$ Calcd for $C_{10}H_{22}CuN_2S_2$: 297.1; Found 297.0; Anal. Calcd. $C_{10}H_{22}CuN_4O_6S_2$: C 28.46, H 5.26, N 13.28. Found: C 28.45, H 4.97, N 13.28.

Synthesis of [Cu(1,4,8,11-tetraazacyclotetradecane)(NO_3)$_2$], 6. To a solution of the 1,4,8,11-tetraazacyclotetradecane (60.1 mg, 0.3 mmol) in dichloromethane (10 mL) was added $Cu(NO_3)_2.3H_2O$ (72.5 mg, 0.3 mmol) and the solution stirred overnight. A purple precipitate formed which was collected by filtration and vacuum dried overnight to give the product as a purple solid (76.3 mg, 65%); ESI-MS *m/z*: [M-2(NO_3)]$^+$ Calcd for $C_{10}H_{24}CuN_4$: 263.1; Found 262.0; Anal. Calcd. $C_{10}H_{24}CuN_6O_6{\cdot}0.75H_2O$: C 29.92, H 6.40, N 20.94. Found: C 30.10, H 6.15, N 20.74.

Crystallographic method. The crystal data for all compounds are compiled in Table S1-S4. Crystals of **1**, **3** and **4** were examined using a Bruker Apex 2000 CCD area detector diffractometer and data was collected using graphite-monochromated Mo-Kα radiation (λ = 0.71073). Intensities were integrated from data recorded on 1° frames by ω rotation. A multiscan method for absorption correction (SADABS)[2] was applied. The structures were solved using SHELXS;[3] the datasets were refined by full-matrix least-squares on reflections with $F^2 \geq 2\sigma(F^2)$ values, with anisotropic displacement parameters for all non-hydrogen atoms, and with constrained riding hydrogen geometries;[4] U_{iso}(H) was set at 1.2 (1.5 for methyl groups) times U_{eq} of the parent atom. The largest features in final difference syntheses were close to heavy atoms and were of no chemical significance. SHELX[3,4] was employed through OLEX2 for structure solution and refinement.[5] ORTEP-3[6] and POV-Ray[7] were employed for molecular graphics. The structures have been deposited with the Cambridge Crystallographic Data Centre (CCDC 2130539-2130541). This information can be obtained free of charge from www.ccdc.cam.ac.uk/data_request/cif.

Electrochemical studies. Cyclic voltammetry measurements were performed using a PalmSens4 potentiostat in a standard three-electrode set-up. A glassy carbon electrode was used as the working electrode (WE), a Pt wire as the counter electrode (CE) and a Ag wire as

the pseudo-reference electrode (RE). Prior to its use, the working electrode was successively polished with sandpaper and subsequent sonicated in DMSO for 10 min. For electrochemical measurements in DMSO tetrabutylammonium hexafluorophosphate ([^{n}Bu$_4$N]PF$_6$, 0.1 M) was used as the supporting electrolyte and in H_2O:DMSO (10:1) potassium chloride (0.1 M) was used. Prior to each experiment, the electrochemical cell as well as the used solvent was degassed with Ar and an Ar atmosphere was maintained throughout the measurement. All cyclic voltammograms were recorded with a scan rate of 100 mV s^{-1}.

UV-vis absorption spectroelectrochemical studies. Spectrochemical experiments were performed in a SEC-2F Spectroelectrochemical flow cell from ALS Co., Ltd attached to a UV-1900i spectrophotometer from Shimadzu via optical fibres, and a PalmSens4 potentiostat. The flow cell was equipped with a standard three-electrode set-up containing a glassy carbon grid electrode as the working electrode (WE), a Pt wire as the counter electrode (CE) and a Ag wire as the pseudo-reference electrode (RE). For electrochemical measurements in DMSO tetrabutylammonium hexafluorophosphate ([^{n}Bu$_4$N]PF$_6$, 0.1 M) was used as the supporting electrolyte and in H_2O:DMSO (10:1) potassium chloride (0.1 M) was used. Controlled potential coulometries (CPC) were performed at a defined potential for 10 or 15 min while UV-vis spectra were recorded every 1 min.

Cell culture. The human mammary epithelial cell lines, HMLER and HMLER-shEcad were kindly donated by Prof. R. A. Weinberg (Whitehead Institute, MIT). The BEAS-2B bronchial epithelium, HEK 293 embryonic kidney, and epithelial breast MCF710A cell lines were acquired from American Type Culture Collection (ATCC, Manassas, VA, USA). HMLER, HMLER-shEcad, and MCF10A cells were maintained in Mammary Epithelial Cell Growth Medium (MEGM) with supplements and growth factors (BPE, hydrocortisone, hEGF, insulin, and gentamicin/amphotericin-B). BEAS-2B cells were cultured in RPMI 1640 medium with 2 mM L-glutamine supplemented with 1% penicillin and 10% fetal bovine serum. HEK 293 cells were cultured in Dulbecco's Modified Eagle's Medium (DMEM) supplemented with 1% penicillin and 10% fetal bovine serum. The cells were grown at 310 K in a humidified atmosphere containing 5% CO_2.

Antiproliferative studies: MTT assay. Exponentially growing cells were seeded at a density of approximately 5×10^3 cells per well in 96-well flat-bottomed microplates and allowed to attach for 24 h prior to addition of compounds. Various concentrations of the test compounds (0.2-100 μM) were added and incubated for 72 h at 37 °C (total volume 200 μL). Stock solutions of the compounds were prepared as 10 mM DMSO solutions and diluted using cell media. The final concentration of DMSO in each well was ≤1 %. After 72 h, 20 μL of MTT (4 mg mL^{-1} in PBS) was added to each well and the plates incubated for an additional 4 h at 37 °C. The media/MTT mixture was eliminated and DMSO (100 μL per well) was added to dissolve the formazan precipitates. The optical density was measured at 550 nm using a 96-well multiscanner autoreader. Absorbance values were normalised to (DMSO-containing) control wells and plotted as concentration of compound versus % cell viability. IC_{50} values were interpolated from the resulting dose dependent curves. The reported IC_{50} values are the average of three independent experiments (n = 18). Appropriate control studies (MTT assay reagent and test compounds in cell culture medium without cells present) were conducted to probe for chemical interference. No chemical interference was observed for the compounds reported in this manuscript. Monitoring the cell confluence and morphology at the end of the MTT assay using an inverted microscope further validated the quantitative potency data (IC_{50} values) determined from the assay.

Tumoursphere formation assay. HMLER-shEcad cells (5 × 10^3) were plated in ultralow-attachment 96-well plates (Corning) and incubated in MEGM supplemented with B27 (Invitrogen), 20 ng mL^{-1} EGF and 4 μg mL^{-1} heparin (Sigma) for 5 days. Studies were also conducted in the presence of **1**, **3**, and salinomycin (0-133 μM). Mammospheres treated with **1**, **3**, or salinomycin (at 2 μM for 5 days) were counted and imaged using an inverted microscope.

Measurement of water-octanol partition coefficient (LogP). The LogP value for **1** and **3** was determined using the shake-flask method and ICP-MS. The 1-octanol used in this experiment was pre-saturated with water. A DMSO solution of **1** or **3** (10 μL, 10 mM) was incubated with 1-octanol (495 μL) and H_2O (495 μL) in a 1.5 mL tube. The tube was shaken at room temperature for 24 h. The two phases were separated by centrifugation and the copper content in the water phase was determined by inductively coupled plasma mass spectrometry (ICP-MS, Thermo Scientific ICAP-Qc quadrupole ICP mass spectrometer).

Cellular uptake. To measure the cellular uptake of **1** and **3** about 1 million HMLER-shEcad cells were treated with **1** or **3** (10 μM) at 37 °C for 24 h. After incubation, the media was removed, the cells were washed with PBS (2 mL × 3) and harvested. The number of cells was counted at this stage, using a haemocytometer. This mitigates any cell death induced by **1** or **3** at the administered concentration and experimental cell loss. The cellular pellets were dissolved in 65% HNO_3 (250 mL) overnight. The cellular pellet of HMLER-shEcad cells treated with **1** or **3** were also used to determine the copper content in the nuclear, cytoplasmic and membrane fractions. The Thermo Scientific NE-PER Nuclear and Cytoplasmic Extraction Kit was used to extract and separate the nuclear, cytoplasmic and membrane fractions. The fractions were dissolved in 65% HNO_3 (250 μL final volume) overnight. All samples were diluted 17-fold with water and analysed using inductively coupled plasma mass spectrometry (ICP-MS, Thermo Scientific ICAP-Qc quadrupole ICP mass spectrometer). Copper levels are expressed as mass of Cu (ng) per million cells. Results are presented as the mean of four determinations for each data point.

Intracellular ROS Assay. Untreated, **1**-treated (IC_{50} value × 2 for 0.5-24 h), **3**-treated (IC_{50} value × 2 for 0.5-24 h), and H_2O_2-treated (150 μM for 0.5-24 h) HMLER-shEcad cells (5 × 10^5 cells/well) grown in six-well plates were incubated with dichloro-dihydro-fluorescein diacetate (DCFH-DA) (20 μM) for 30 min. The cells were then washed with PBS (1 mL), harvested by trypsinisation, and analysed using a FACSCanto II flow cytometer (BD Biosciences) (10,000 events per sample were acquired) at the University of Leicester FACS Facility. The FL1 channel was used to assess intracellular ROS levels. Cell populations were analysed using the FlowJo software (Tree Star).

Immunoblotting Analysis. HMLER-shEcad cells (5 × 10^6) were incubated with **1** (5-20 μM for 72 h) at 37 °C. HMLER-shEcad cells were harvested and isolated as pellets. SDS-PAGE loading buffer (64 mM Tris-HCl (pH 6.8)/ 9.6% glycerol/ 2%SDS/ 5% β-mercaptoethanol/ 0.01% Bromophenol Blue) was added to the pellets, and this was incubated at 95 °C for 10 min. Cell lysates were resolved by 4-20 % sodium dodecylsulphate polyacylamide gel electrophoresis (SDS-PAGE; 200 V for 25 min) followed by electro transfer to polyvinylidene difluoride membrane, PVDF (350 mA for 1 h). Membranes were blocked in 5% (w/v) non-fat milk in PBST (PBS/0.1% Tween 20) and incubated with the appropriate primary antibodies (Cell Signalling Technology). After incubation with horseradish peroxidase-conjugated secondary antibodies (Cell Signalling Technology), immune

complexes were detected with the ECL detection reagent (BioRad) and analysed using a chemiluminescence imager (Bio-Rad ChemiDoc Imaging System).

COX-2 Expression Assay. HMLER-shEcad cells were seeded in 6-well plates (at a density of 5 × 10^5 cells/ mL) and the cells were allowed to attach overnight. The cells were treated with lipopolysaccharide (LPS) (2.5 μg/ L for 24 h), and then treated with **1** (IC_{50} value) or diclofenac (10-40 μM) and incubated for a further 48 h. The cells were then harvested by trypsinisation, fixed with 4% paraformaldehyde (at 37 °C for 10 min), permeabilized with ice-cold methanol (for 30 min), and suspended in PBS (200 μL). The Alexa Fluor® 488 nm labelled anti-COX-2 antibody (5 μL) was then added to the cell suspension and incubated in the dark for 1 h. The cells were then washed with PBS (1 mL) and analysed using a FACSCanto II flow cytometer (BD Biosciences) (10,000 events per sample were acquired) at the University of Leicester FACS Facility. The FL1 channel was used to assess COX-2 expression. Cell populations were analysed using the FlowJo software (Tree Star).

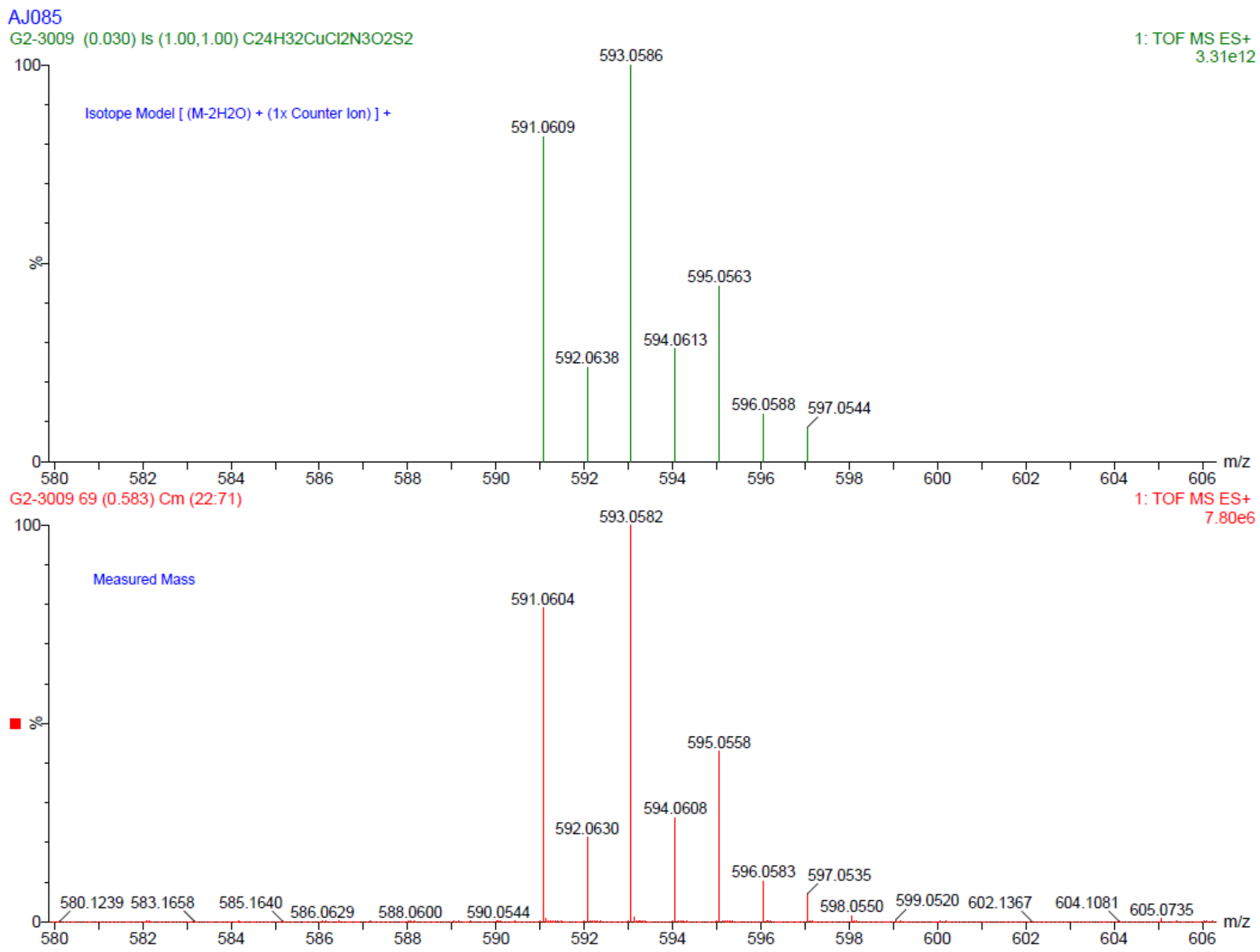

Fig. S1 (Top) Theoretical isotope model for [**1**-diclofenac]$^+$ ($C_{24}H_{32}Cl_2CuN_3O_2S_2$) and (bottom) the experimentally determined high-resolution ESI-QTOF mass spectrum for the [**1**-diclofenac]$^+$ ion.

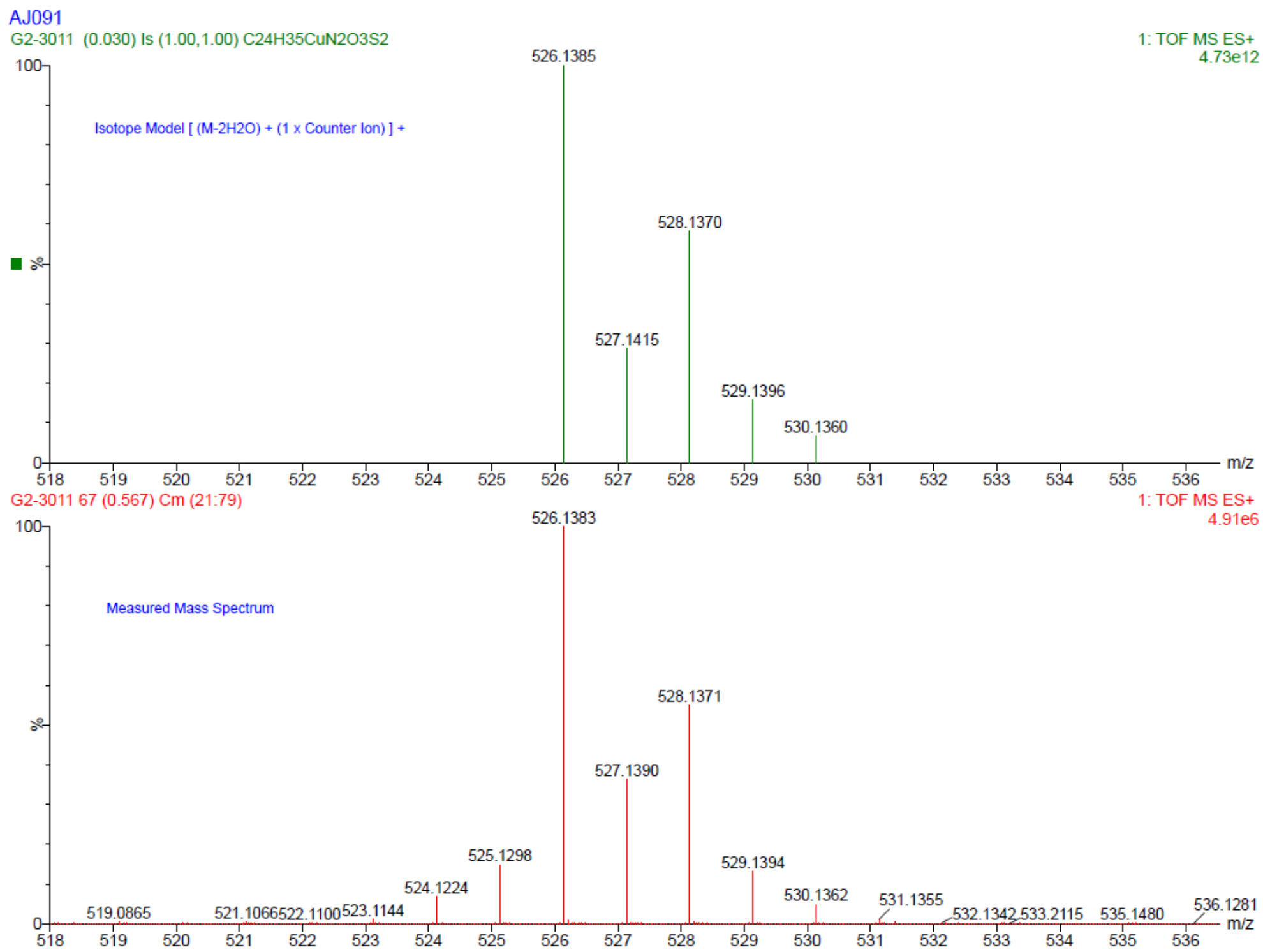

Fig. S2 (Top) Theoretical isotope model for [**2**-naproxen]$^+$ ($C_{24}H_{35}CuN_2O_3S_2$) and (bottom) the experimentally determined high-resolution ESI-QTOF mass spectrum for the [**2**-naproxen]$^+$ ion.

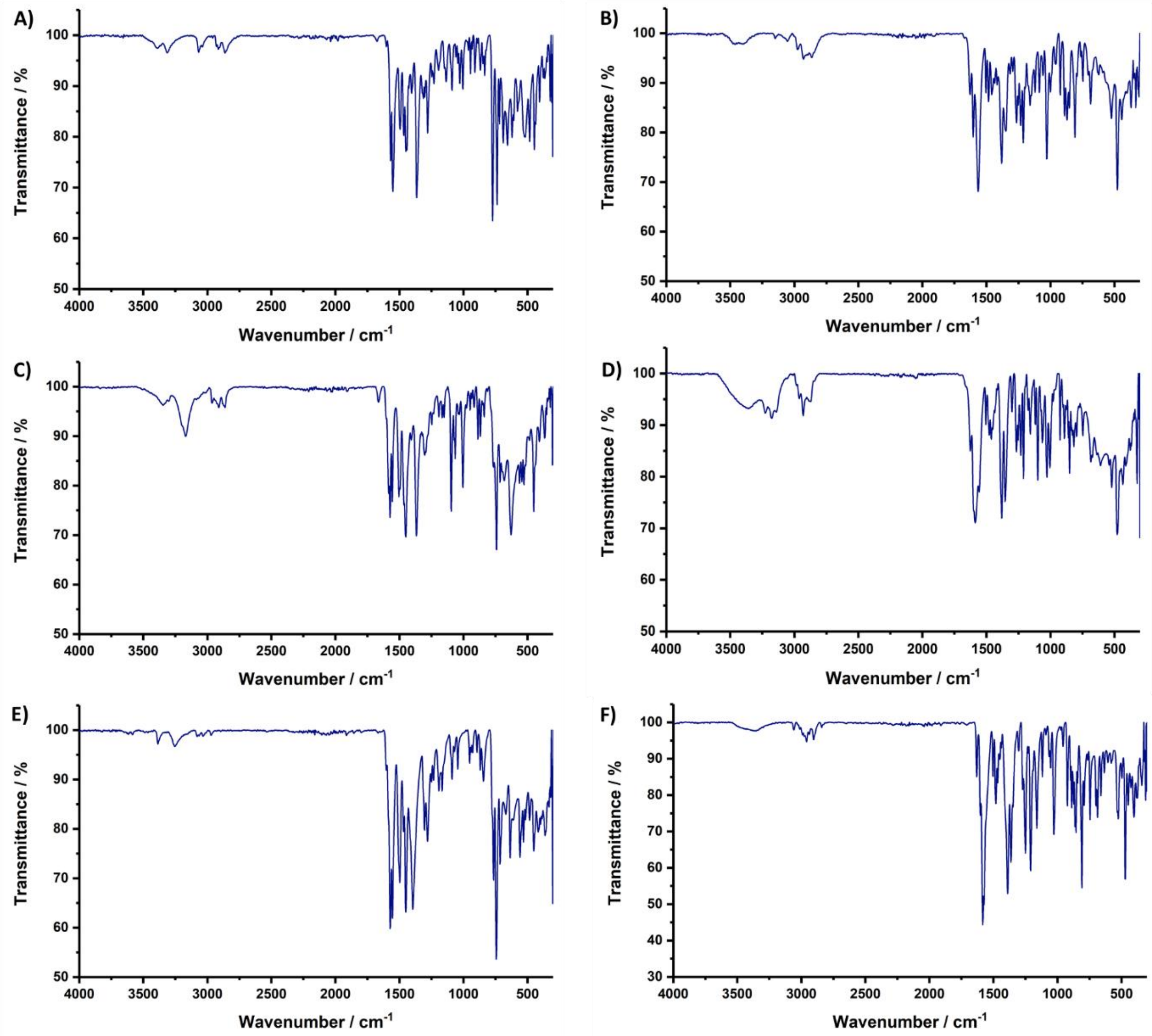

Fig. S3 ATR spectrum of (A) **1**, (B) **2**, (C) **3**, (D) **4**, (E) sodium diclofenac, and (F) sodium naproxen in the solid form.

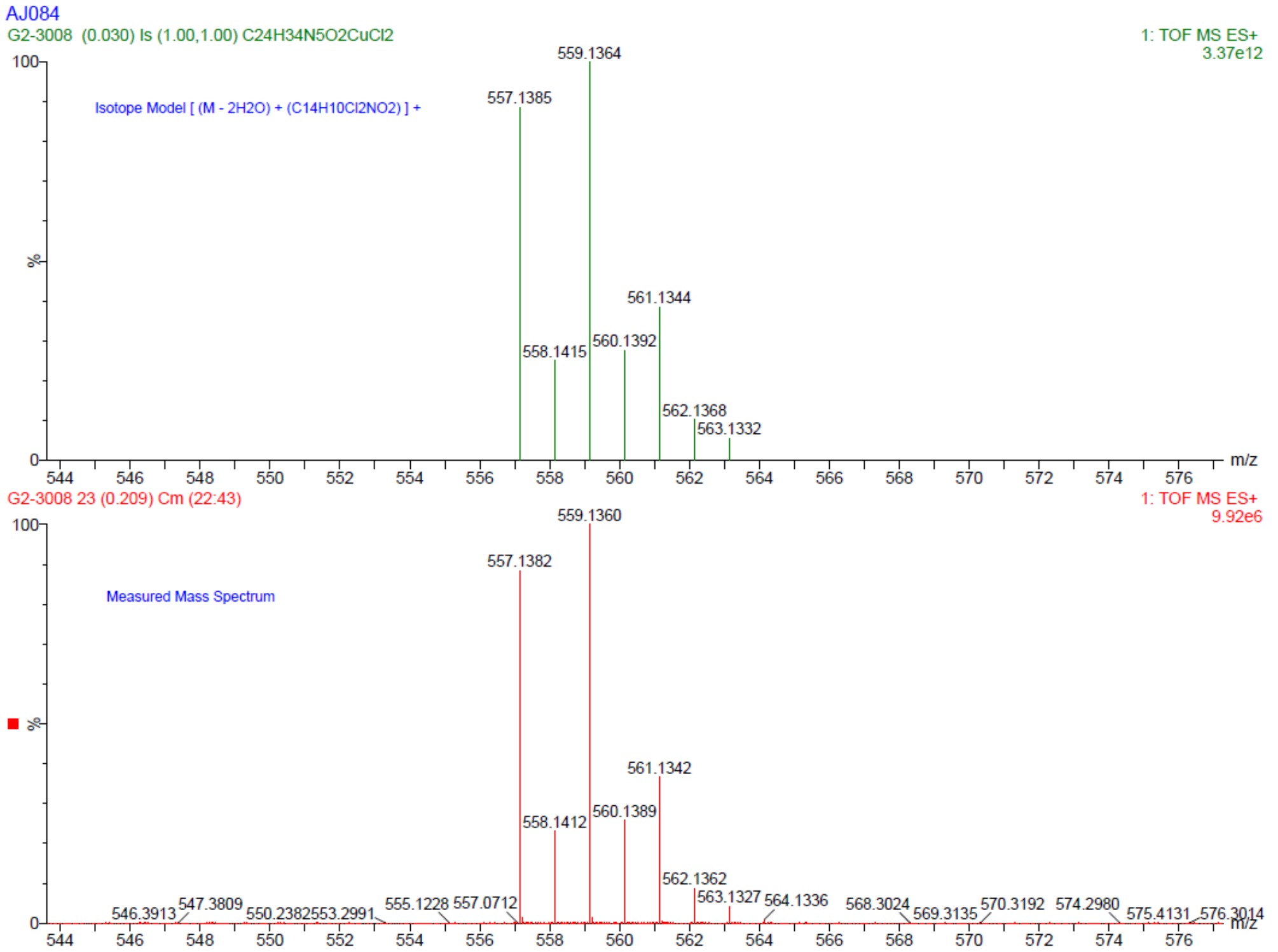

Fig. S4 (Top) Theoretical isotope model for [**3**-2H_2O-diclofenac]$^+$ ($C_{24}H_{34}Cl_2CuN_5O_2$) and (bottom) the experimentally determined high-resolution ESI-QTOF mass spectrum for the [**3**-2H_2O-diclofenac]$^+$ ion.

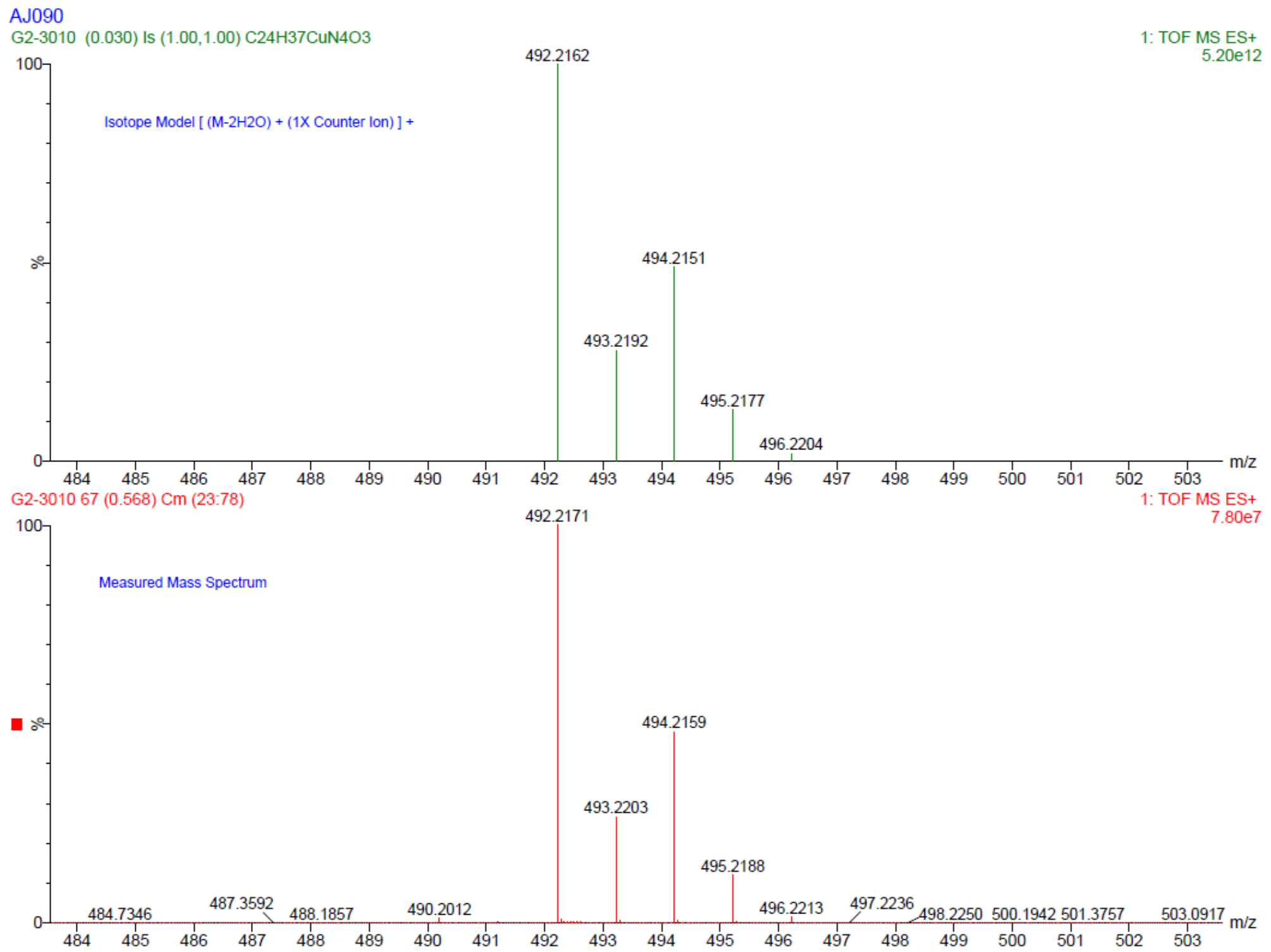

Fig. S5 (Top) Theoretical isotope model for [**4**-2H_2O-naproxen]$^+$ ($C_{24}H_{37}CuN_4O_3$) and (bottom) the experimentally determined high-resolution ESI-QTOF mass spectrum for the [**4**-2H_2O-naproxen]$^+$ ion.

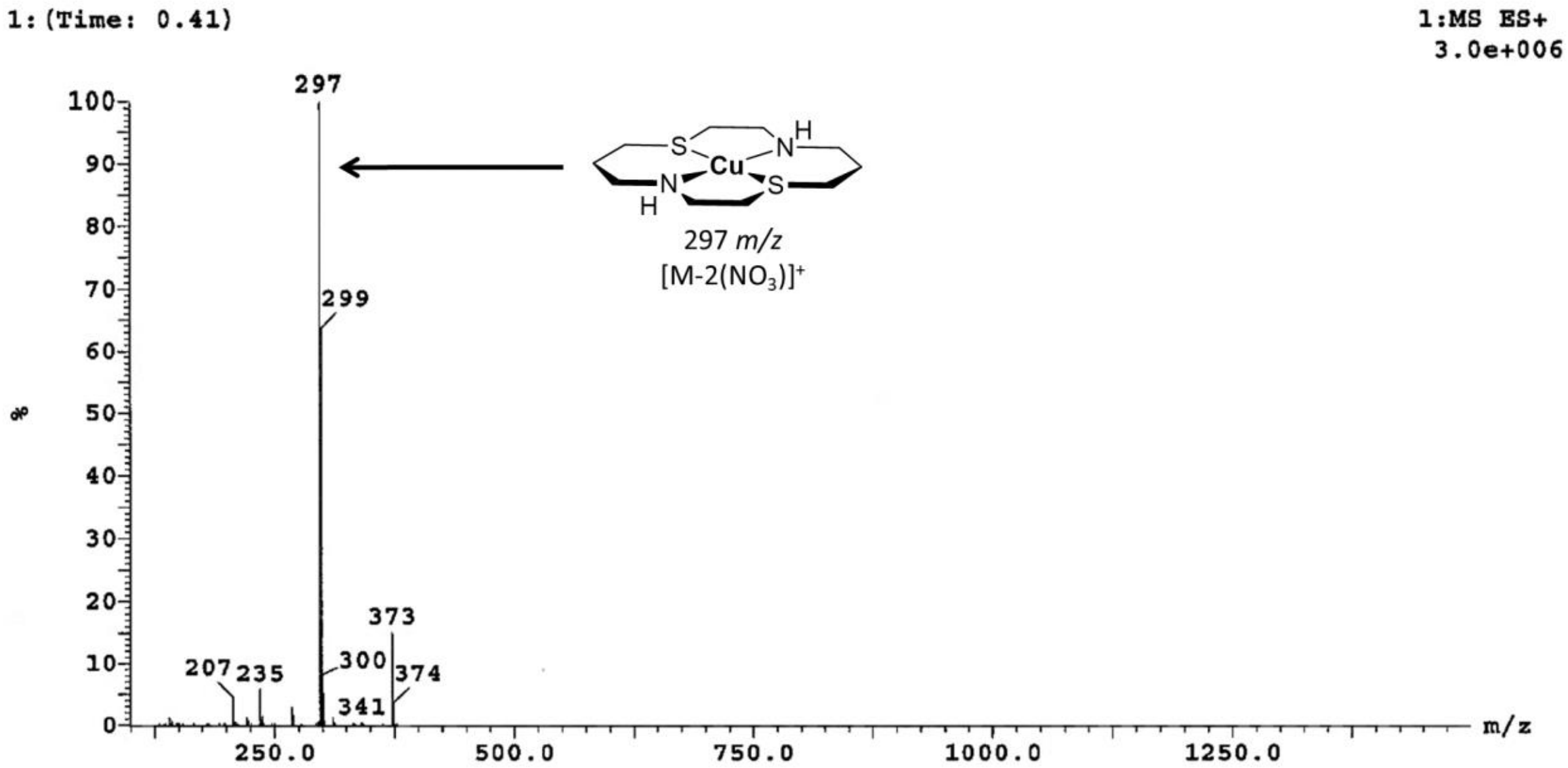

Fig. S6 ESI mass spectrum (positive mode) for [Cu(1,8-dithia-4,11-diazacyclotetradecane)$(NO_3)_2$], **5** in methanol.

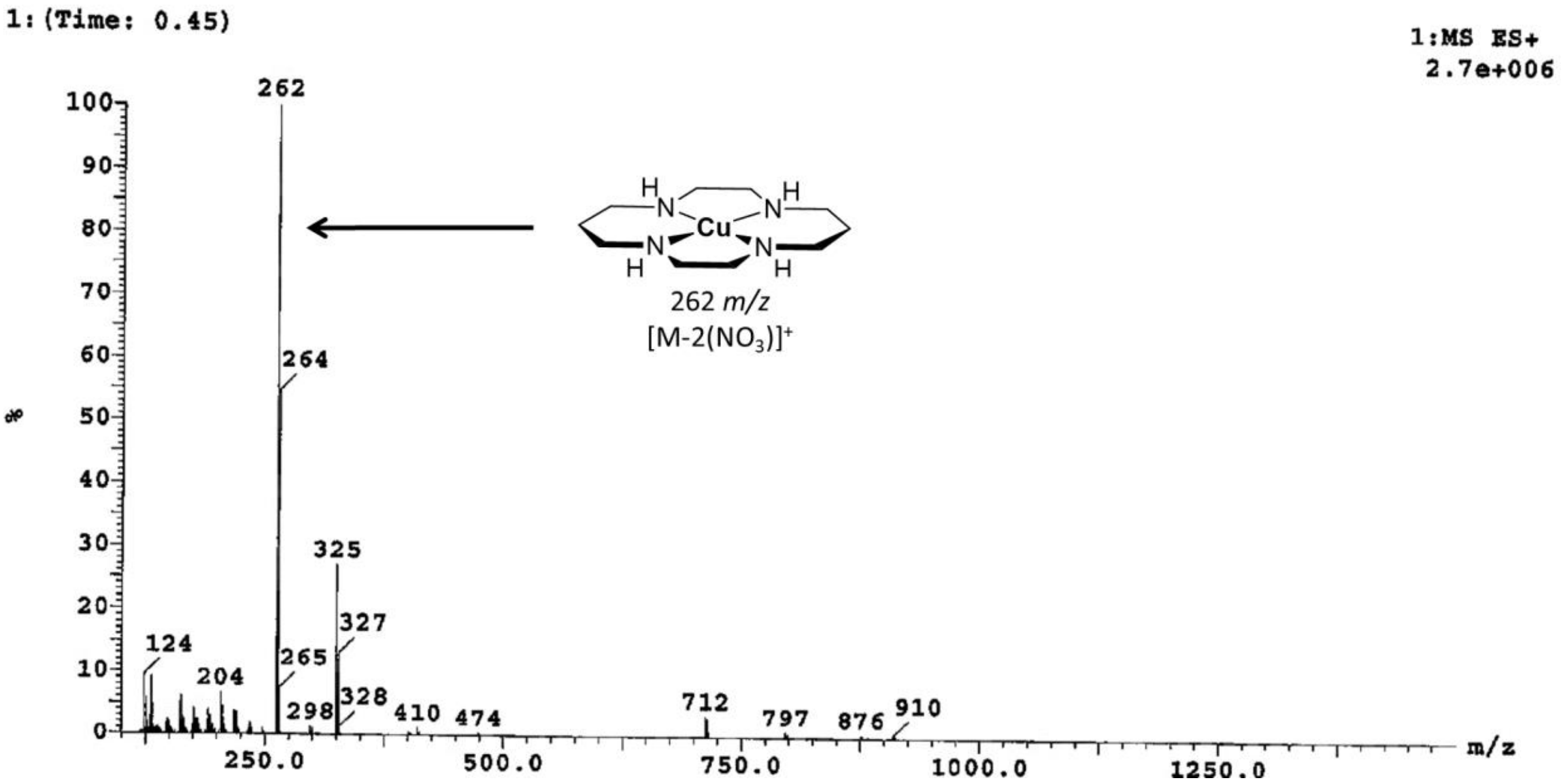

Fig. S7 ESI mass spectrum (positive mode) for [Cu(1,4,8,11-tetraazacyclotetradecane)$(NO_3)_2$], **6** in methanol.

Table S1. Crystallographic data for **1**, **3** and **4**.

[a]Conventional $R = \Sigma||Fo| - |Fc||/\Sigma|Fo|$; $Rw = [\Sigma w(Fo^2 - Fc^2)^2/\Sigma w(Fo^2)^2]^{1/2}$; $S = [\Sigma w(Fo^2 - Fc^2)^2$/no. data – no. params)]$^{1/2}$ for all data.

**Metal complex**	**1**	**3**	**4**
CCDC No.	2130539	2130540	2130541
Formula	$C_{38}H_{44}Cl_4CuN_4O_5S_2$	$C_{38}H_{48}Cl_4CuN_6O_6$	$C_{38}H_{62}CuN_4O_{12}$
Fw	906.23	890.16	830.45
Crystal system	monoclinic	monoclinic	monoclinic
Space group	*C*2/*c*	$P2_1/c$	$P2_1$
a, Å	19.076(5)	18.109(15)	7.917(4)
b, Å	15.842(5)	7.269(7)	30.22(2)
c, Å	15.133(4)	15.999(14)	8.837(5)
α, °	90	90	90
β, °	120.693(4)	101.71(2)	99.700(14)
γ, °	90	90	90
V, Å^3	3933(2)	2062(3)	2084(2)
Z	4	2	2
ρ_{calc}, g/cm^3	1.531	1.434	1.323
μ, mm^{-1}	0.983	0.841	0.588
F(*000*)	1876	926	886
No. of reflections (unique)	15933 (4278)	15546 (4034)	16525 (8085)
S[a]	1.05	0.87	0.82
$R_1(wR_2)$ $(F^2 > 2\sigma(F^2))$	0.0368, 0.0920	0.1013 (0.2788)	0.0826, 0.1718
R_{int}	0.039	0.360	0.174
Min./max. diff map, Å^{-3}	–0.54, 0.34	–1.33, 0.64	–0.59, 0.56

Table S2. Selected bond lengths (Å) and angles (°) for **1**.

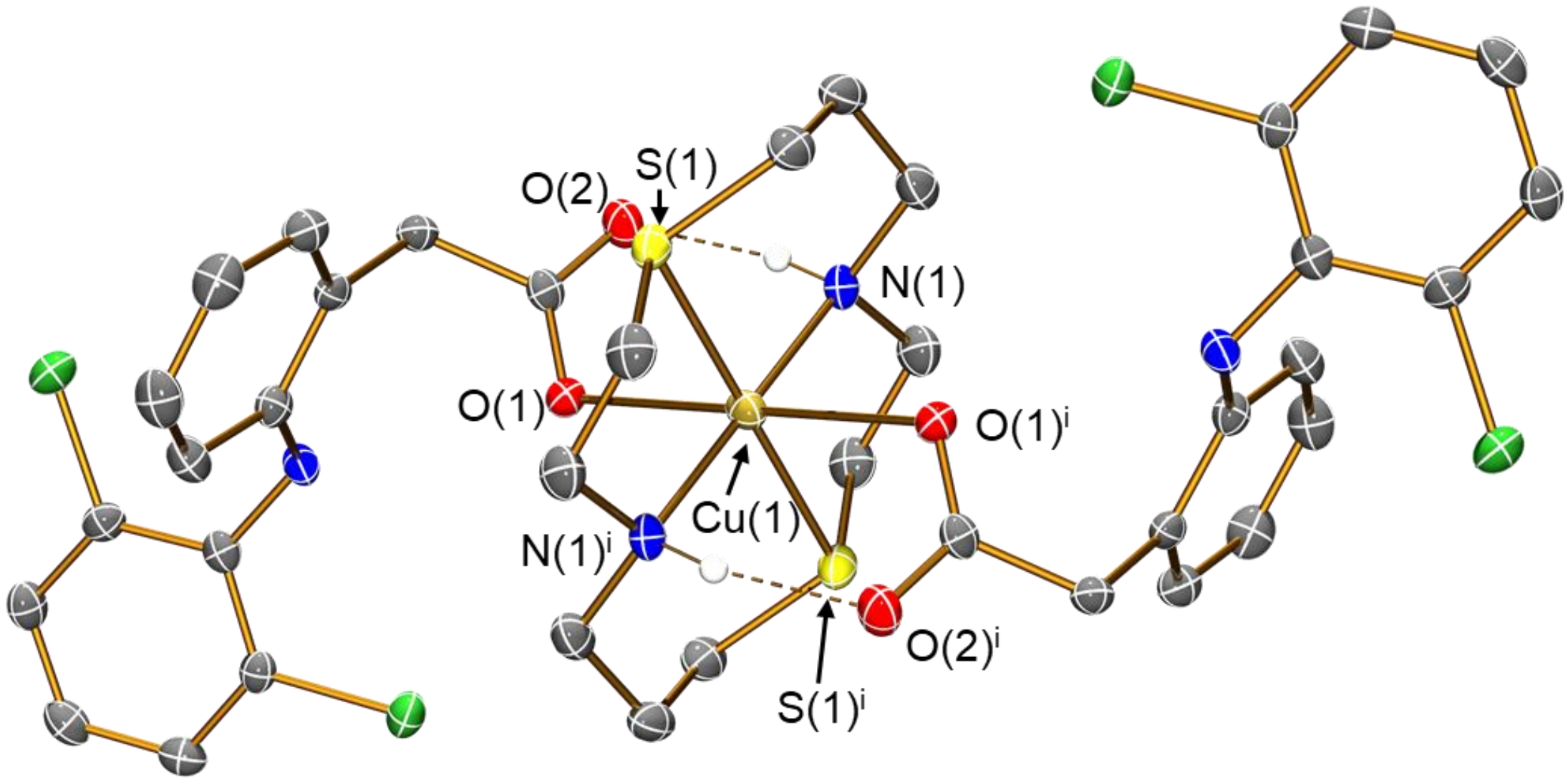

Cu(1)-S(1)	2.3303(8)	Cu(1)-S(1)^{i}	2.3303(8)
Cu(1)-O(1)	2.5148(17)	Cu(1)-O(1)^{i}	2.5148(17)
Cu(1)-N(1)	2.0319(18)	Cu(1)-N(1)^{i}	2.0319(18)
S(1)-Cu(1)-S(1)^{i}	180.0	N(1)-Cu(1)-S(1)^{i}	92.77(5)
S(1)^{i}-Cu(1)-O(1)	91.20(4)	N(1)^{i}-Cu(1)-S(1)	92.77(5)
S(1)-Cu(1)-O(1)	88.80(4)	N(1)-Cu(1)-O(1)	90.00(6)
N(1)-Cu(1)-S(1)	87.23(5)	N(1)^{i}-Cu(1)-O(1)	90.00(6)
N(1)^{i}-Cu(1)-S(1)^{i}	87.23(5)	N(1)^{i}-Cu(1)-N(1)	180.00(9)

Table S3. Selected bond lengths (Å) and angles (°) for **3**.

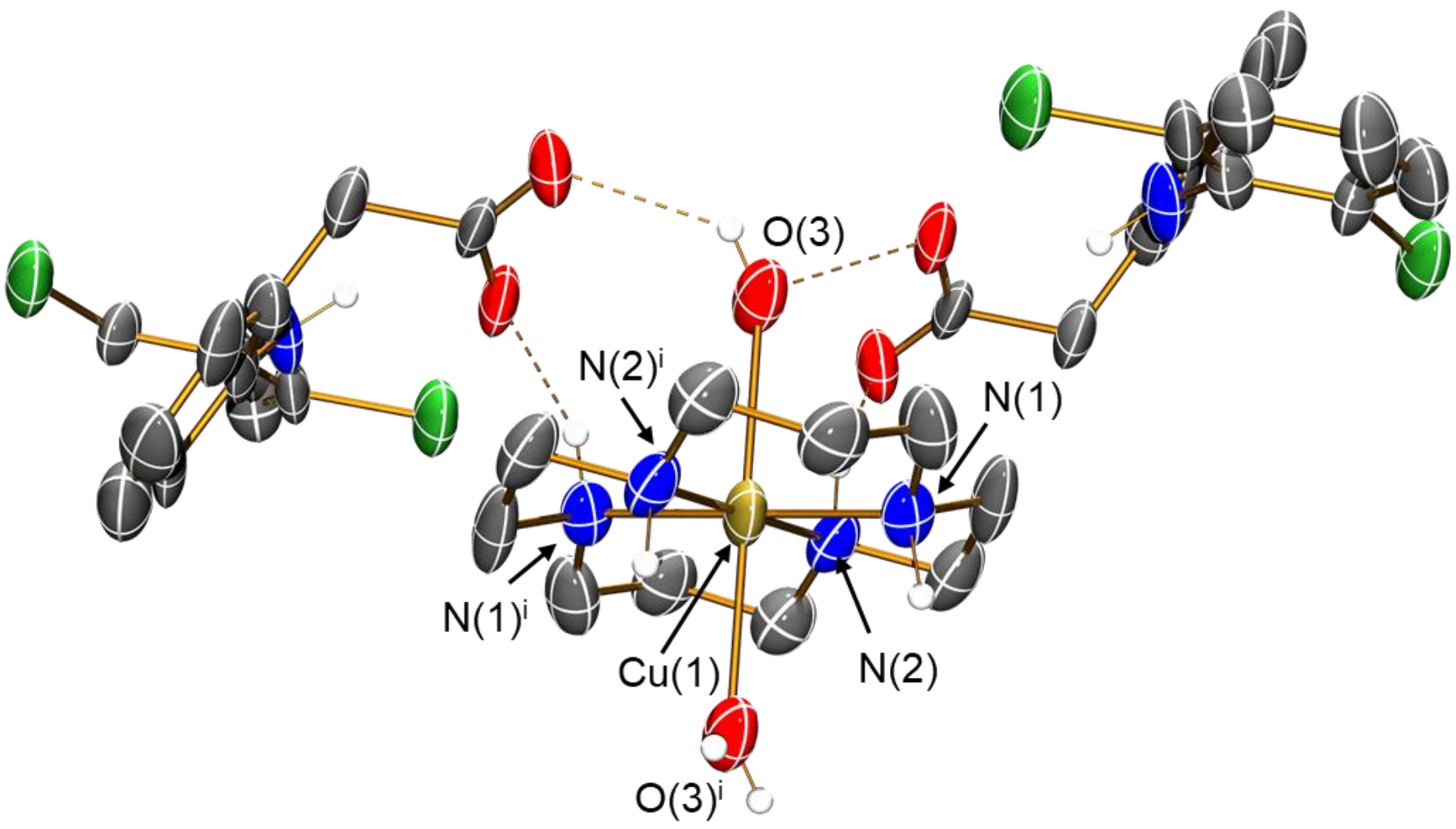

Cu(1)-N(1)^{i}	2.013(8)	Cu(1)-N(2)	2.035(9)
Cu(1)-N(1)	2.013(8)	Cu(1)-O(3)^{i}	2.524(8)
Cu(1)-N(2)^{i}	2.035(9)	Cu(1)-O(3)	2.524(8)
N(1)^{i}-Cu(1)-N(1)	180.0(5)	N(1)-Cu(1)-O(3)^{i}	91.8(3)
N(1)-Cu(1)-N(2)^{i}	86.5(4)	N(1)^{i}-Cu(1)-O(3)	91.8(3)
N(1)-i-Cu(1)-N(2)	86.5(4)	N(1)^{i}-Cu(1)-O(3)^{i}	88.2(3)
N(1)-Cu(1)-N(2)	93.5(4)	N(2)-Cu(1)-O(3)	88.5(3)
N(1)^{i}-Cu(1)-N(2)^{i}	93.5(4)	N(2)-Cu(1)-O(3)^{i}	91.5(3)
N(2)-Cu(1)-N(2)^{i}	180.0	N(2)^{i}-Cu(1)-O(3)	88.5(3)
O(3)-Cu(1)-O(3)^{i}	180.0(5)	N(2)^{i}-Cu(1)-O(3)^{i}	91.5(3)
N(1)-Cu(1)-O(3)	88.2(3)		

Table S4. Selected bond lengths (Å) and angles (°) for **4**.

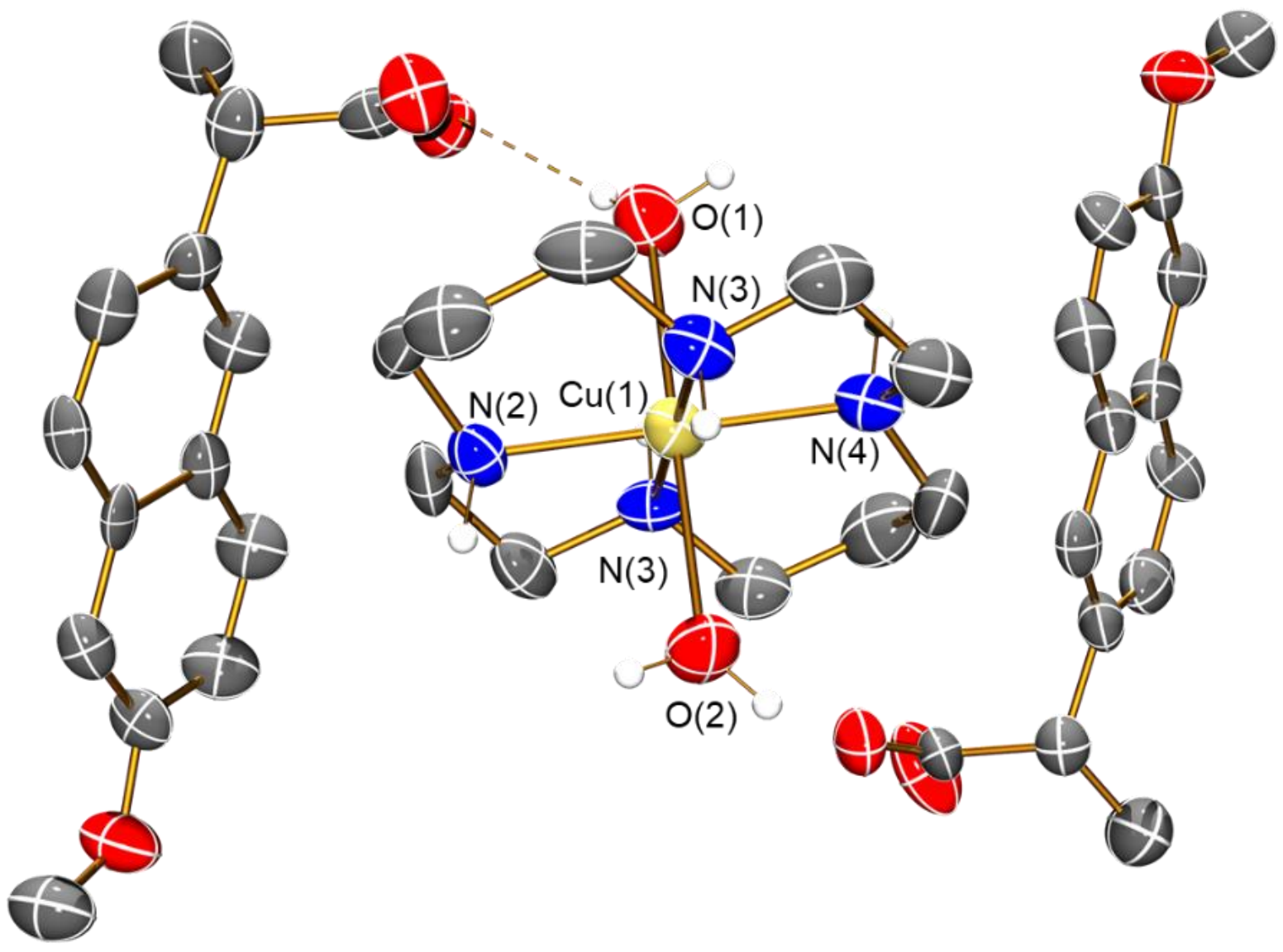

Cu(1)-O(1)	2.428(8)	Cu(1)-N(2)	2.019(11)
Cu(1)-O(2)	2.473(8)	Cu(1)-N(3)	2.026(9)
Cu(1)-N(1)	2.017(9)	Cu(1)-N(4)	2.019(12)
O(1)-Cu(1)-O(2)	179.4(4)	N(2)-Cu(1)-N(3)	85.3(5)
N(1)-Cu(1)-O(1)	93.5(4)	N(2)-Cu(1)-N(4)	179.2(5)
N(1)-Cu(1)-O(2)	86.0(4)	N(3)-Cu(1)-O(1)	86.7(4)
N(1)-Cu(1)-N(2)	94.7(5)	N(3)-Cu(1)-O(2)	93.8(3)
N(1)-Cu(1)-N(3)	179.8(6)	N(4)-Cu(1)-O(1)	86.9(4)
N(1)-Cu(1)-N(4)	86.0(5)	N(4)-Cu(1)-O(2)	93.0(4)
N(2)-Cu(1)-O(1)	92.8(4)	N(4)-Cu(1)-N(3)	93.9(5)
N(2)-Cu(1)-O(2)	87.4(4)		

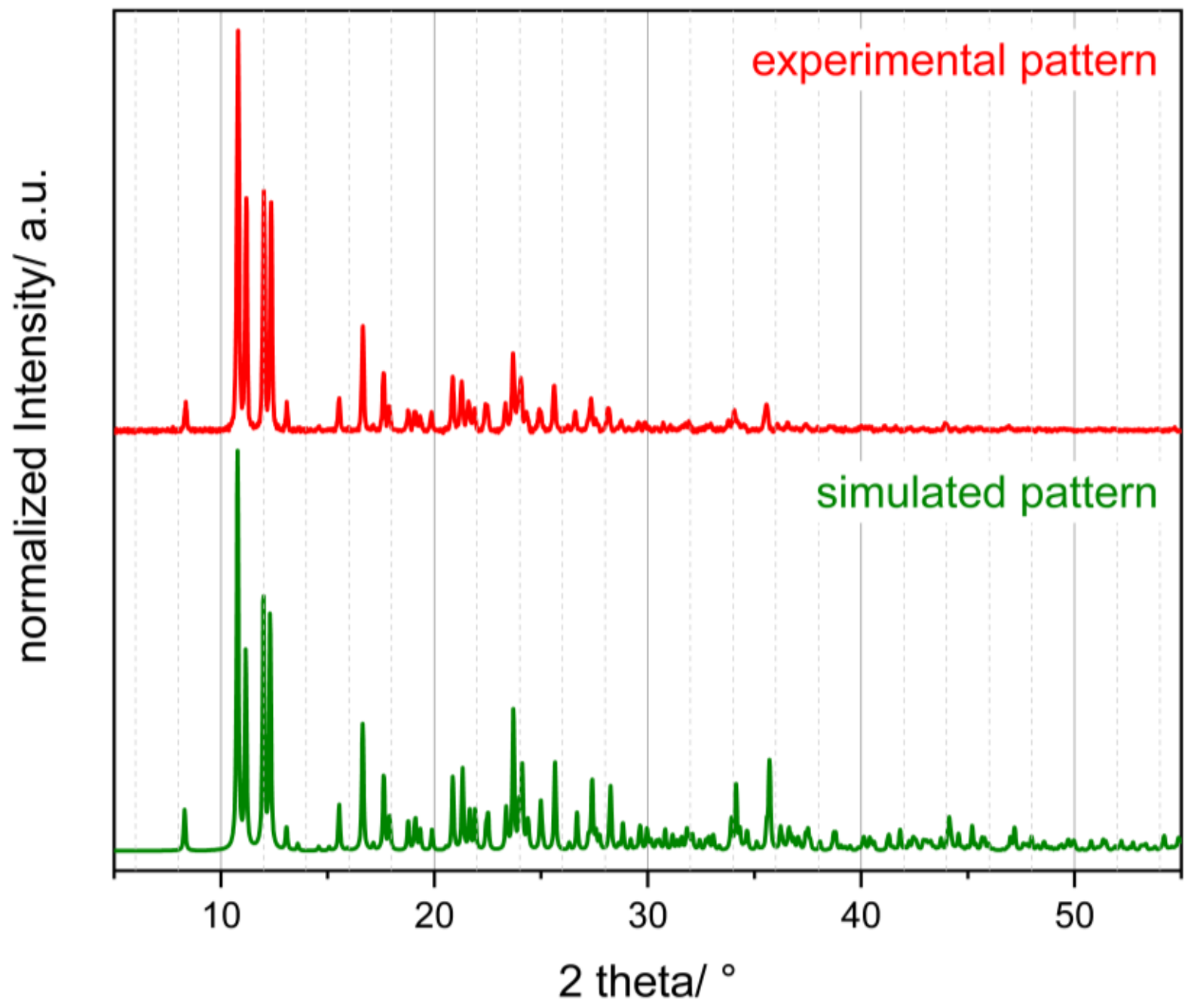

Fig. S8 (Top) Experimentally determined powder X-ray diffraction pattern of **1** and (bottom) simulated pattern based on the single crystal X-ray diffraction analysis of **1**.

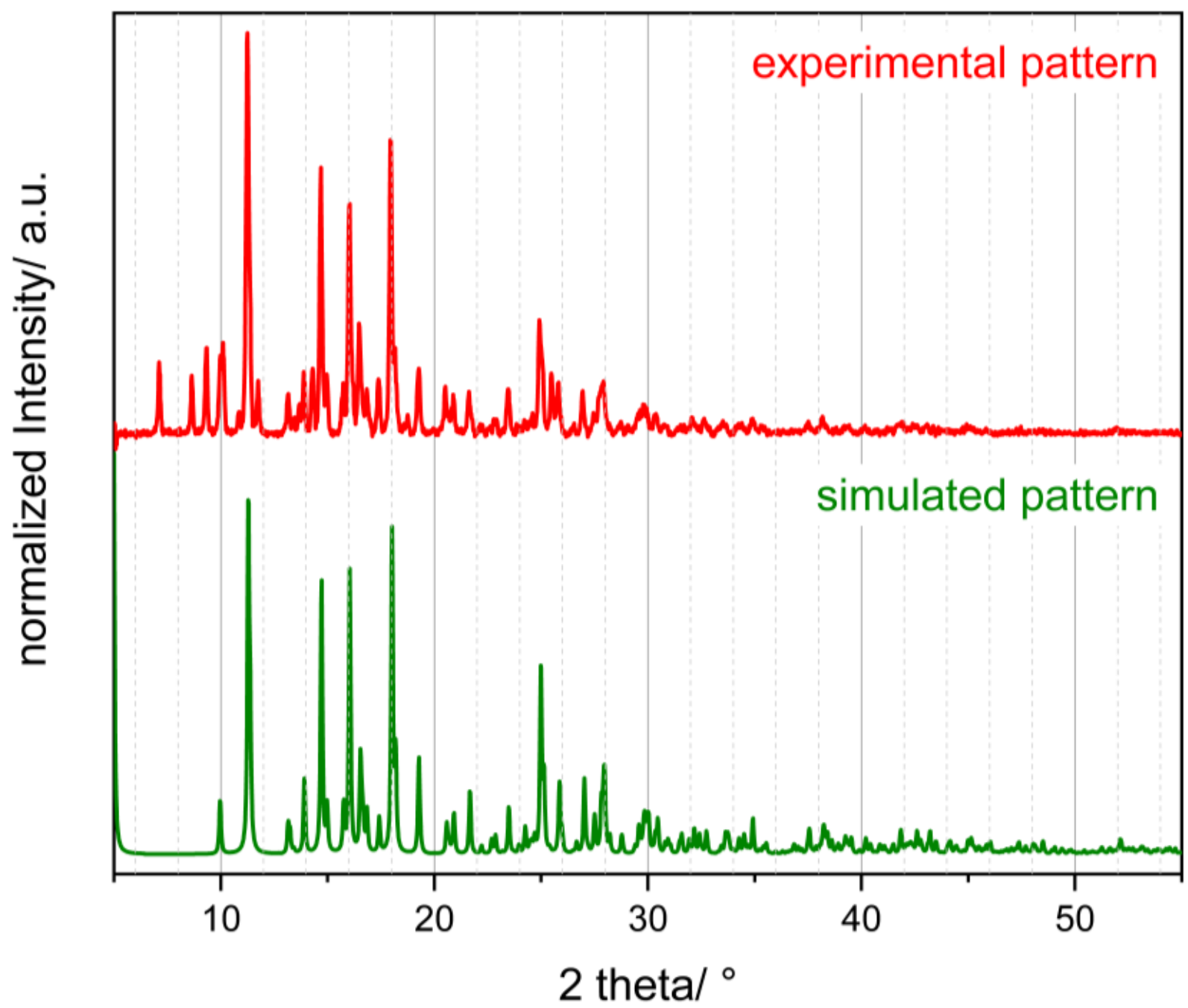

Fig. S9 (Top) Experimentally determined powder X-ray diffraction pattern of **3** and (bottom) simulated pattern based on the single crystal X-ray diffraction analysis of **3**.

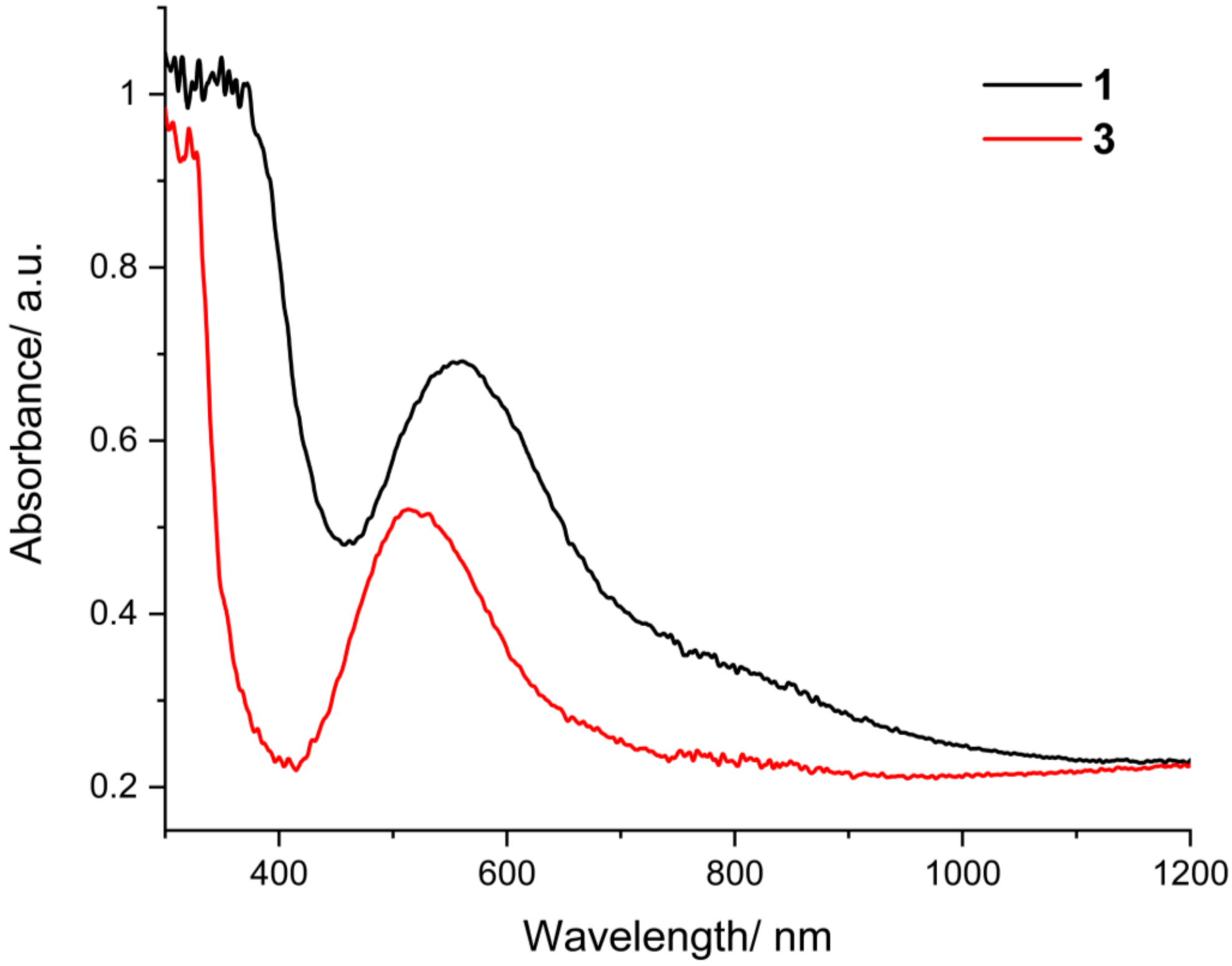

Fig. S10. Solid state UV-vis spectra of **1** and **3**.

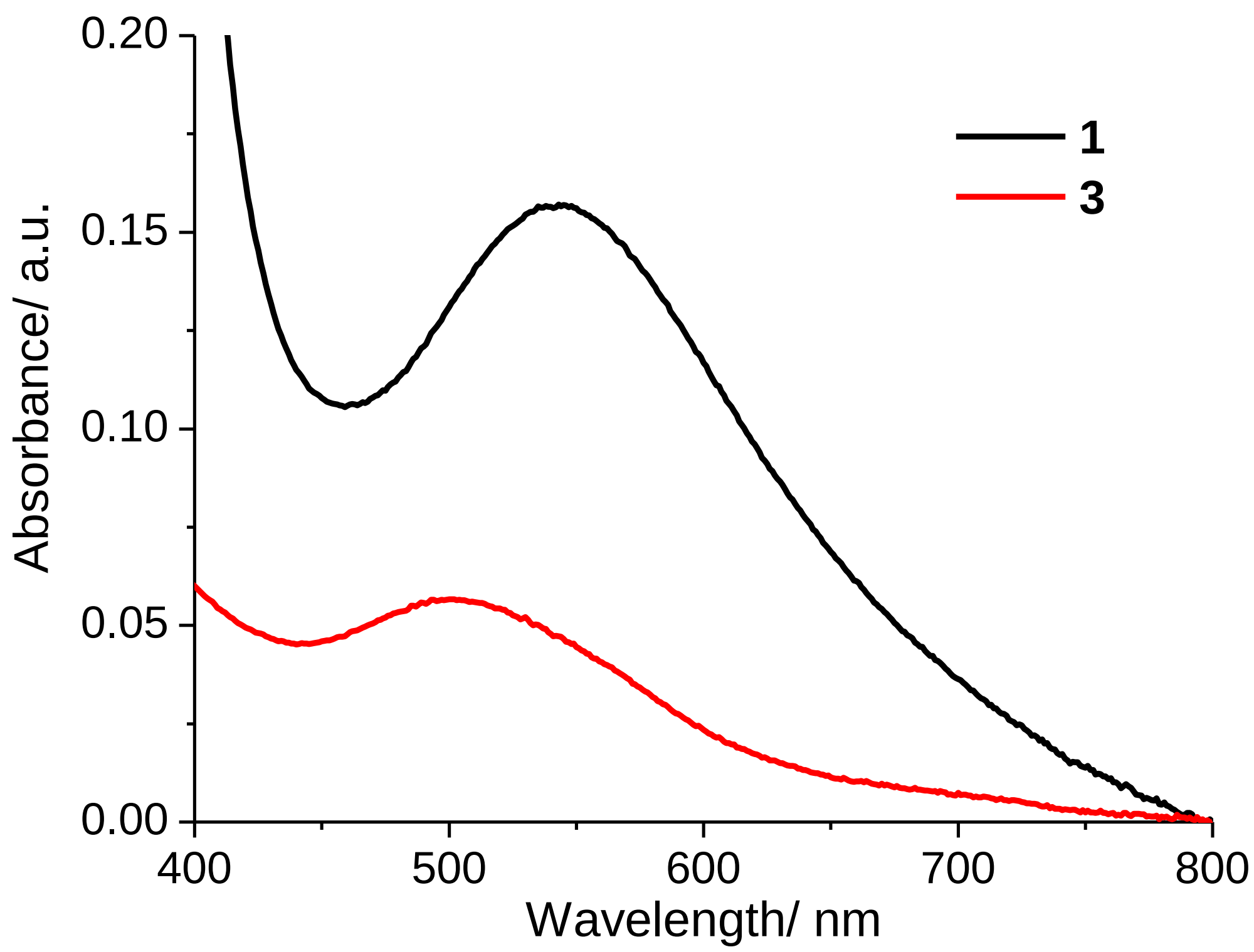

Fig. S11 UV-vis spectra of **1** and **3** (1 mM) in H_2O:DMSO (10:1).

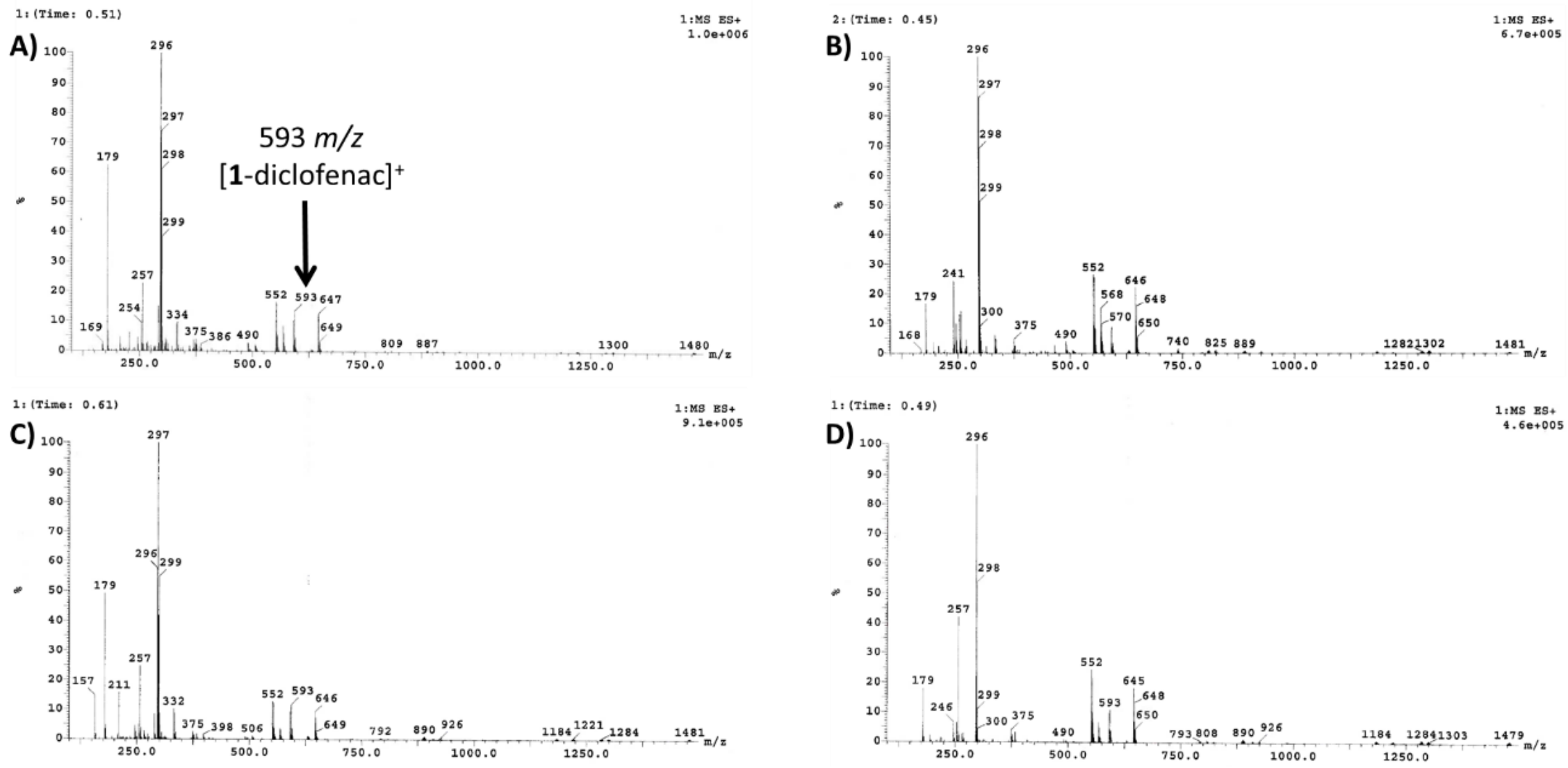

Fig. S12 ESI mass spectra (positive mode) of **1** (500 μM) in H_2O:DMSO (10:1) (A) before and after incubation for (B) 24 h, (C) 48 h, and (D) 72 h at 37 °C.

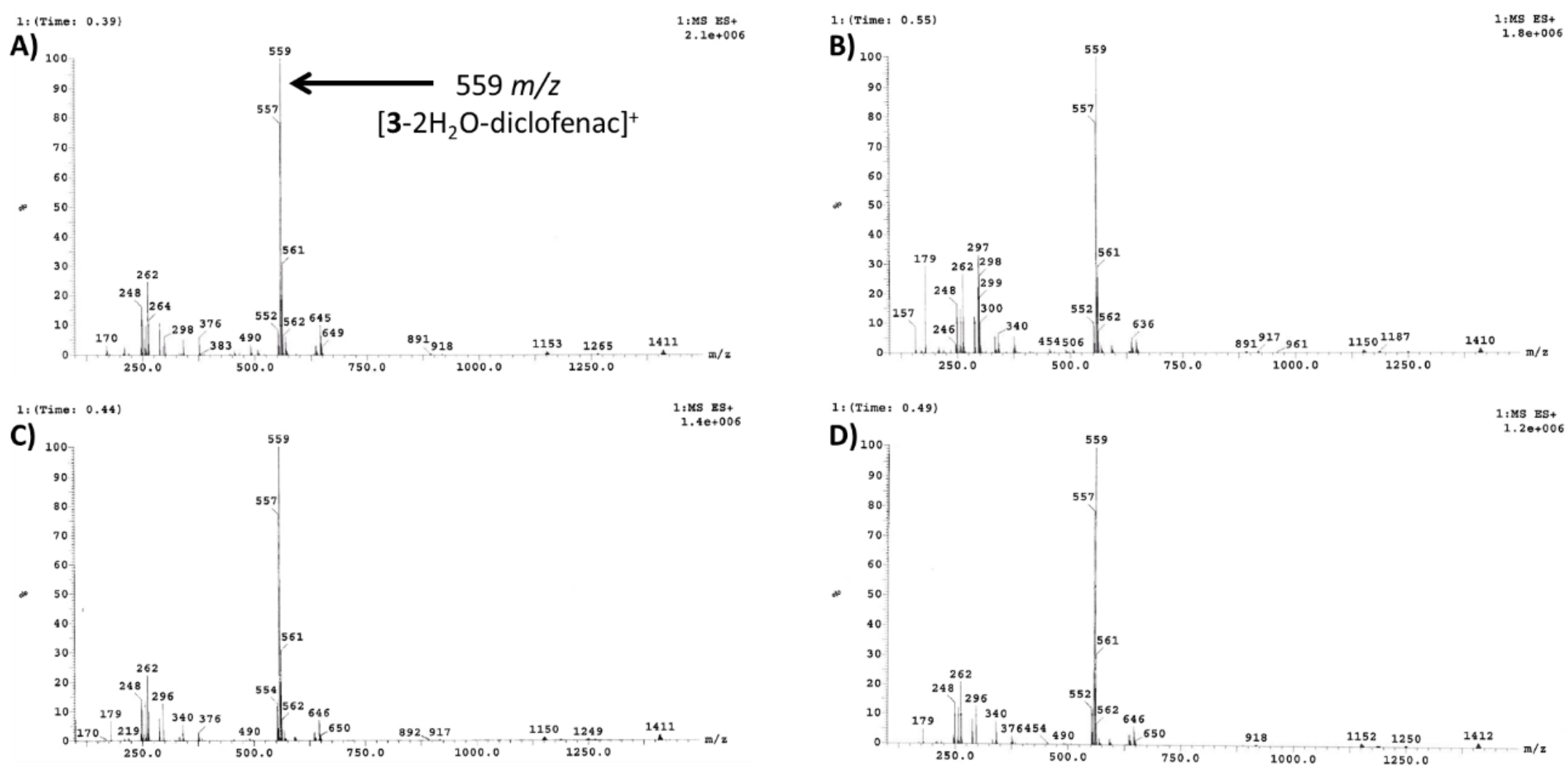

Fig. S13 ESI mass spectra (positive mode) of **3** (500 μM) in H_2O:DMSO (10:1) (A) before and after incubation for (B) 24 h, (C) 48 h, and (D) 72 h at 37 °C.

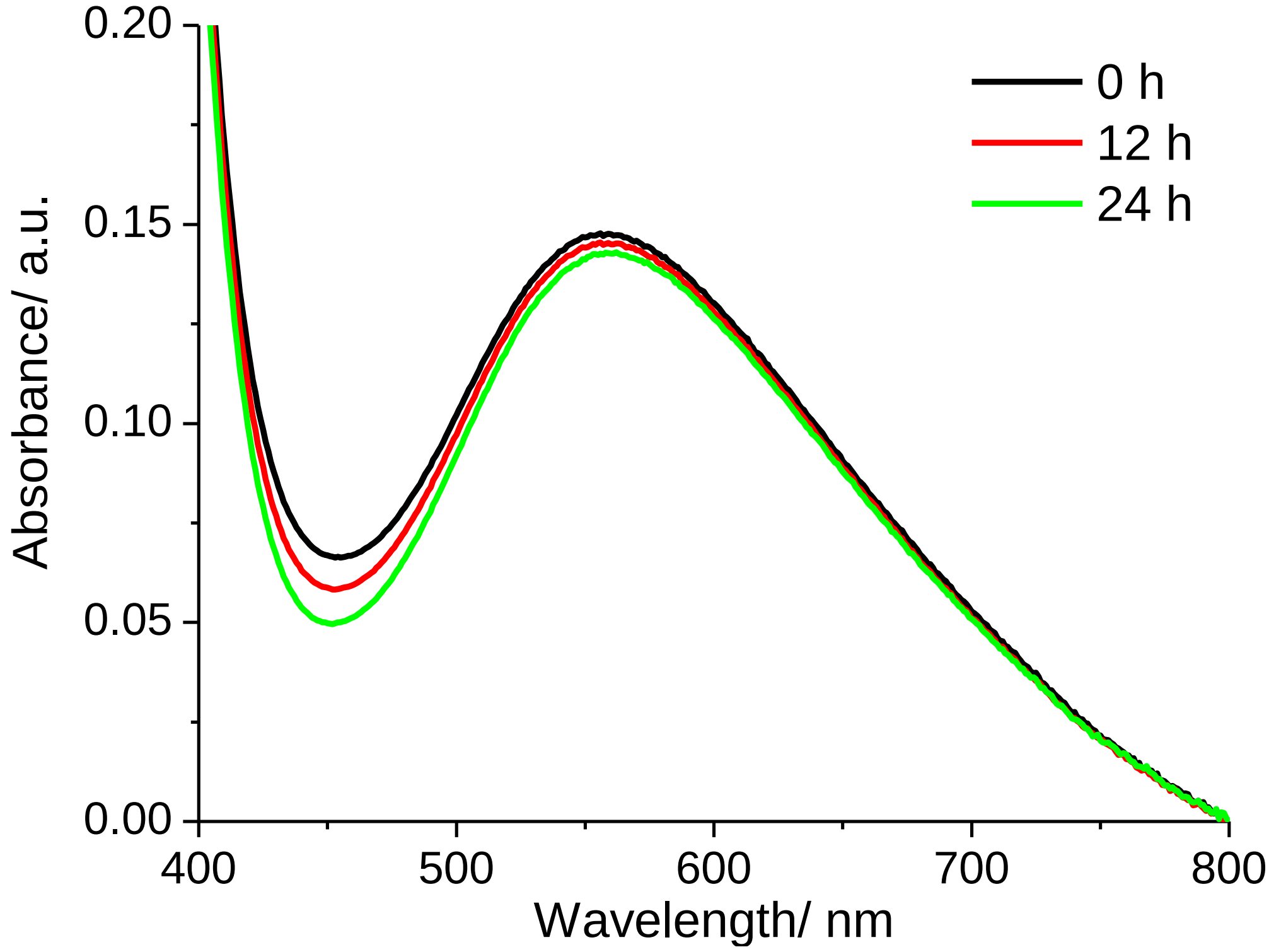

Fig. S14 UV-vis spectra of **1** (1 mM) in PBS:DMSO (200:1) over the course of 24 h.

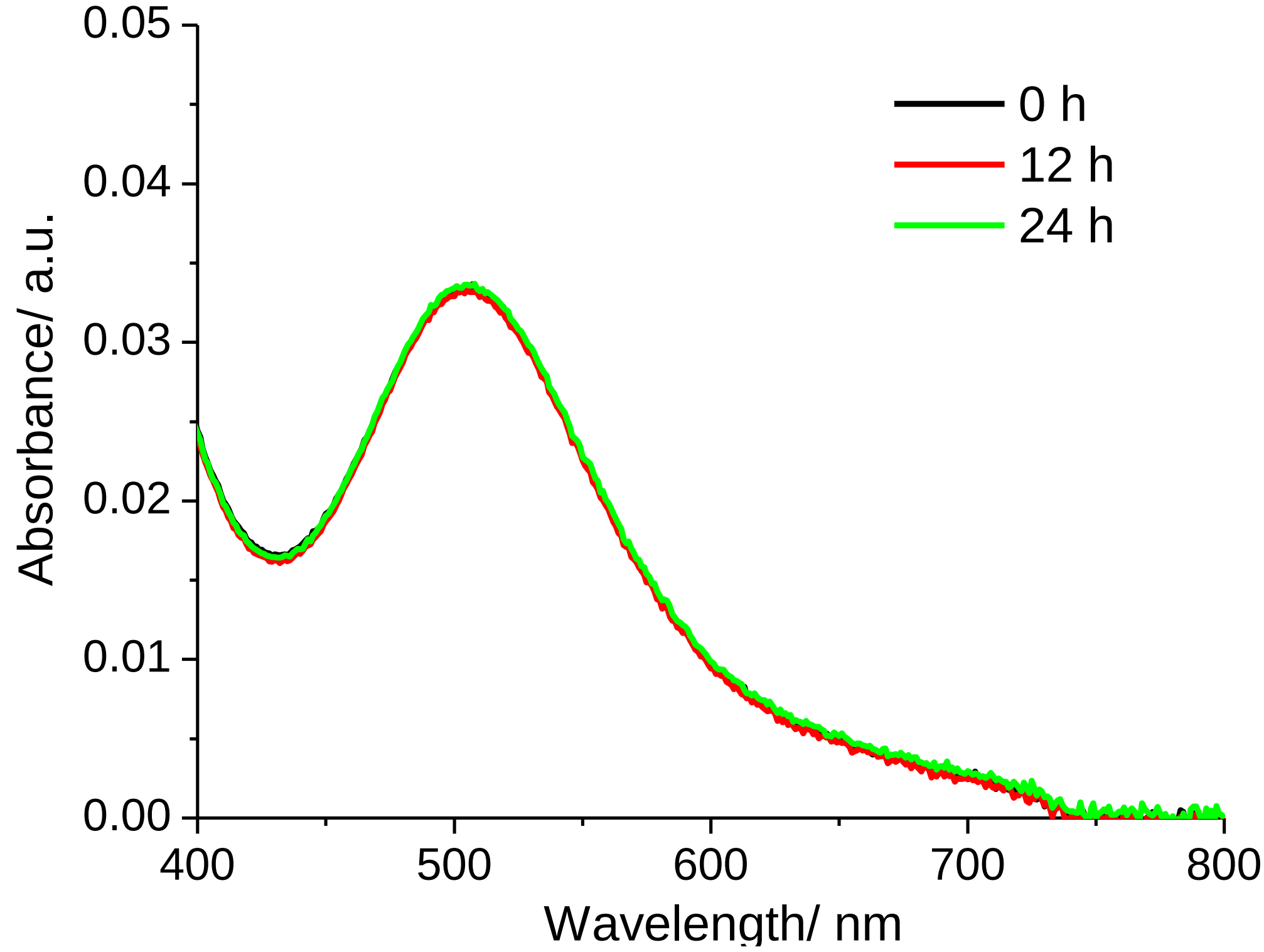

Fig. S15 UV-vis spectra of **3** (1 mM) in PBS:DMSO (200:1) over the course of 24 h.

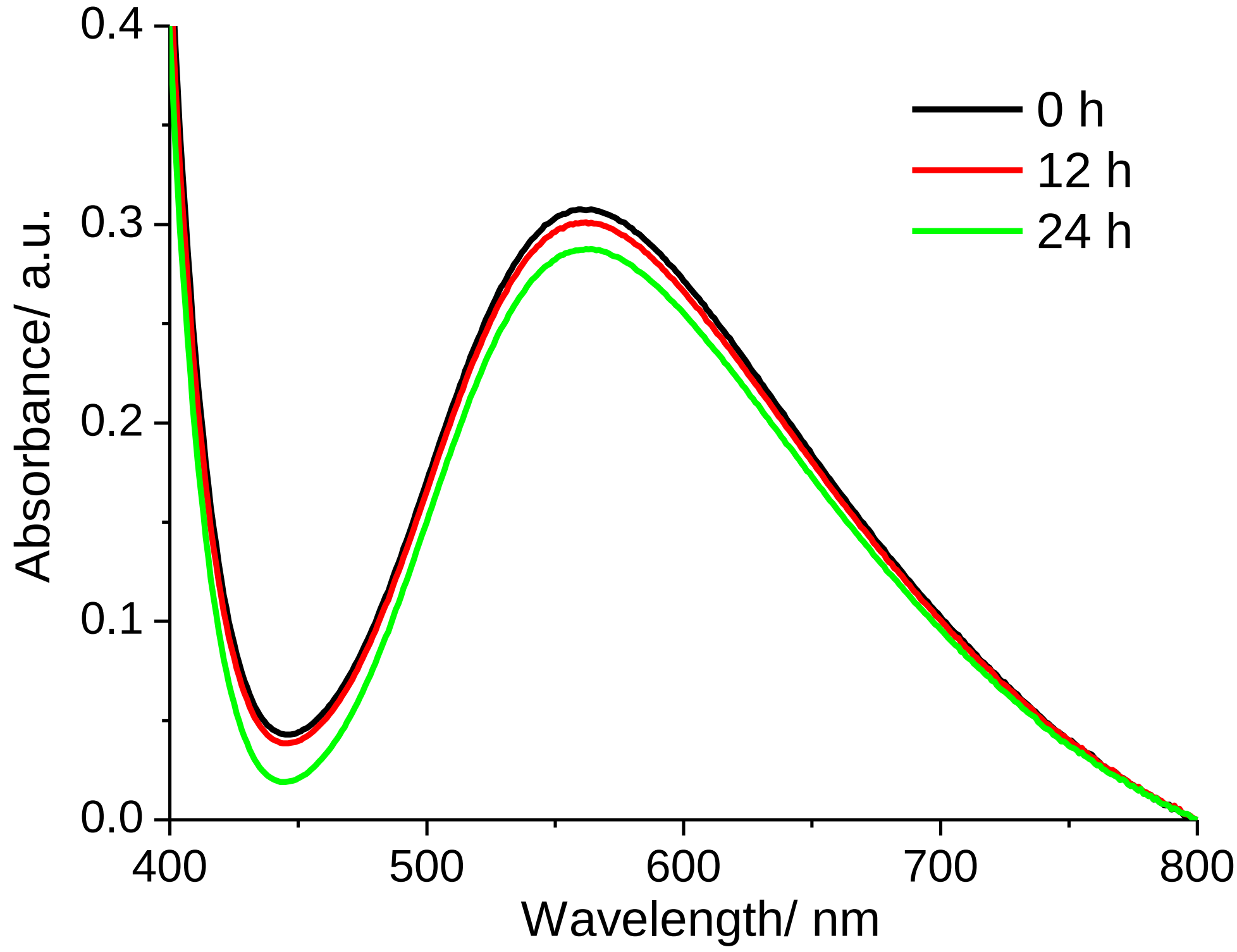

Fig. S16 UV-vis spectra of **1** (1 mM) in MEGM:DMSO (200:1) over the course of 24 h.

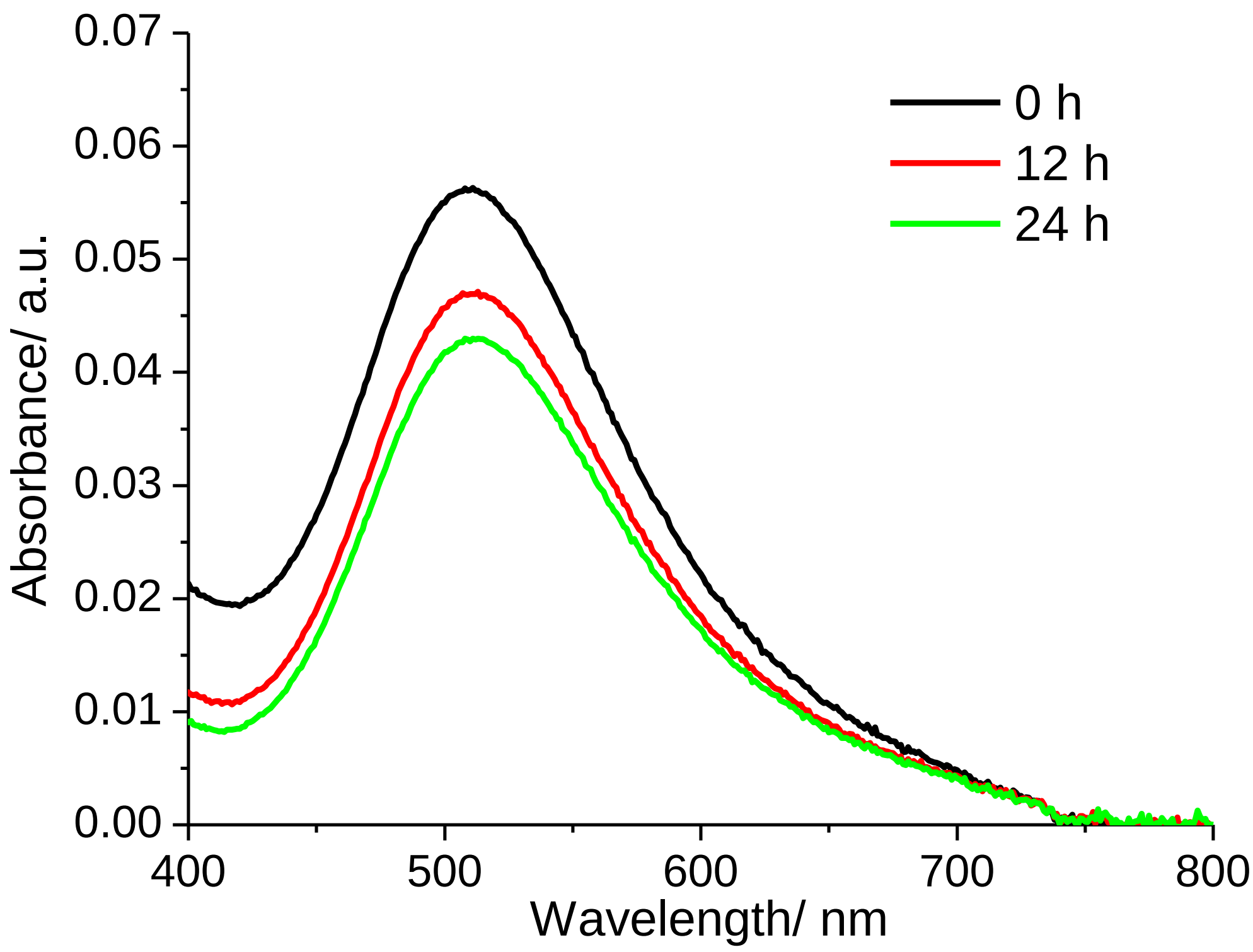

Fig. S17 UV-vis spectra of **3** (1 mM) in MEGM:DMSO (200:1) over the course of 24 h.

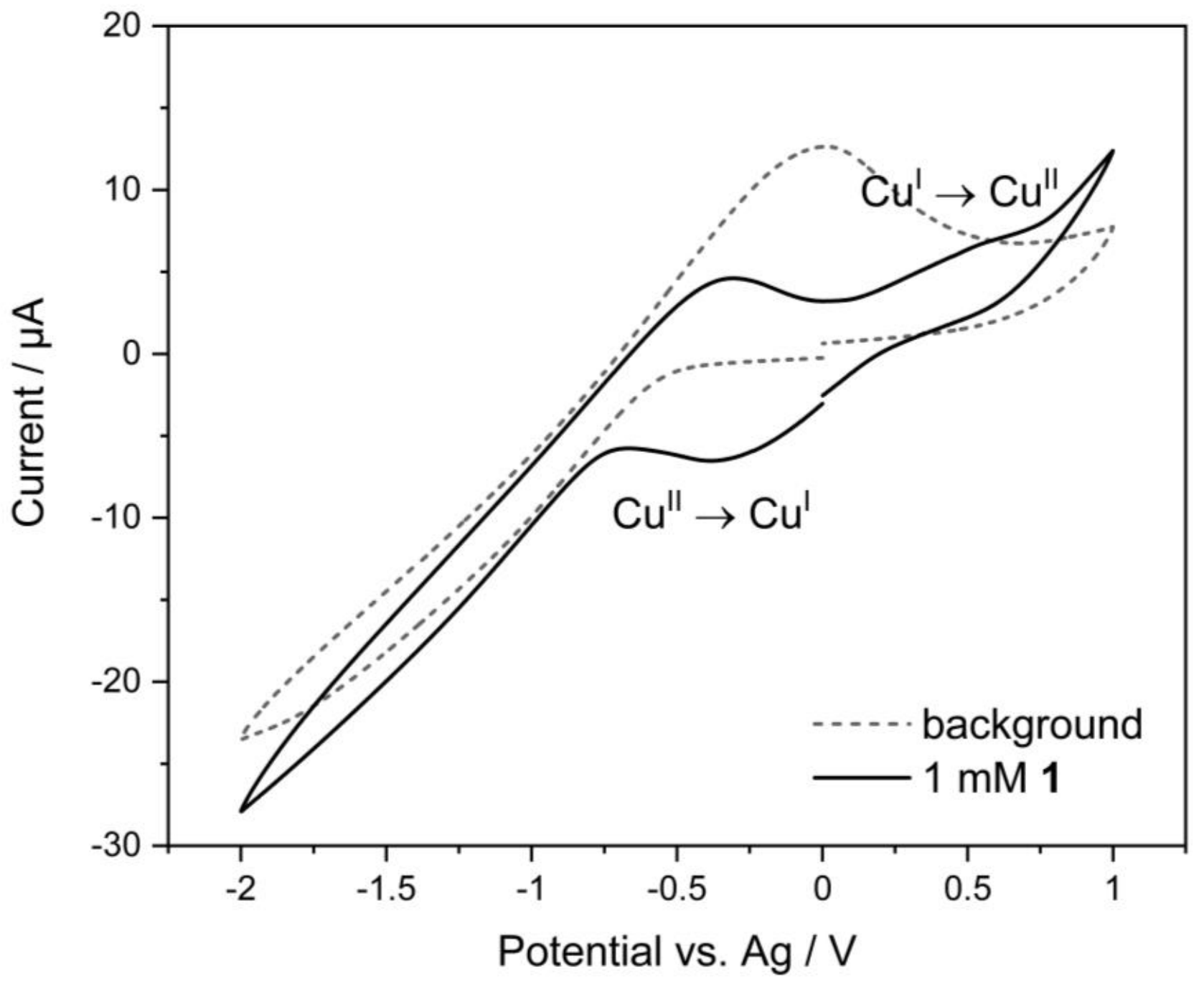

Fig. S18 Cyclic voltammogram of **1** (1 mM) within the SEC-UV-vis set-up in DMSO using $TBAPF_6$ (0.1 M) as the supporting electrolyte.

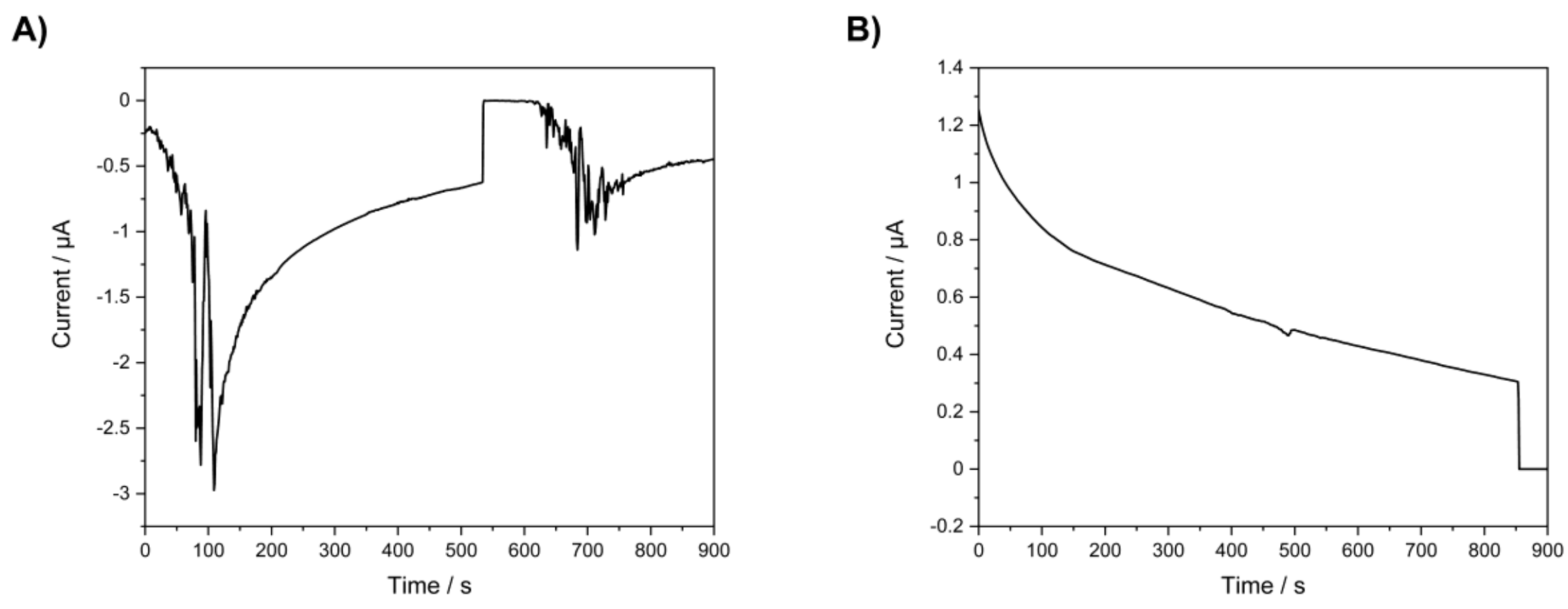

Fig. S19 Controlled potential coulometry of **1** (1 mM) in DMSO with $TBAPF_6$ (0.1 M) for 15 min at -0.2 V vs. Ag (A) and 0.2 V vs. Ag (B) within the SEC-UV-vis set-up.

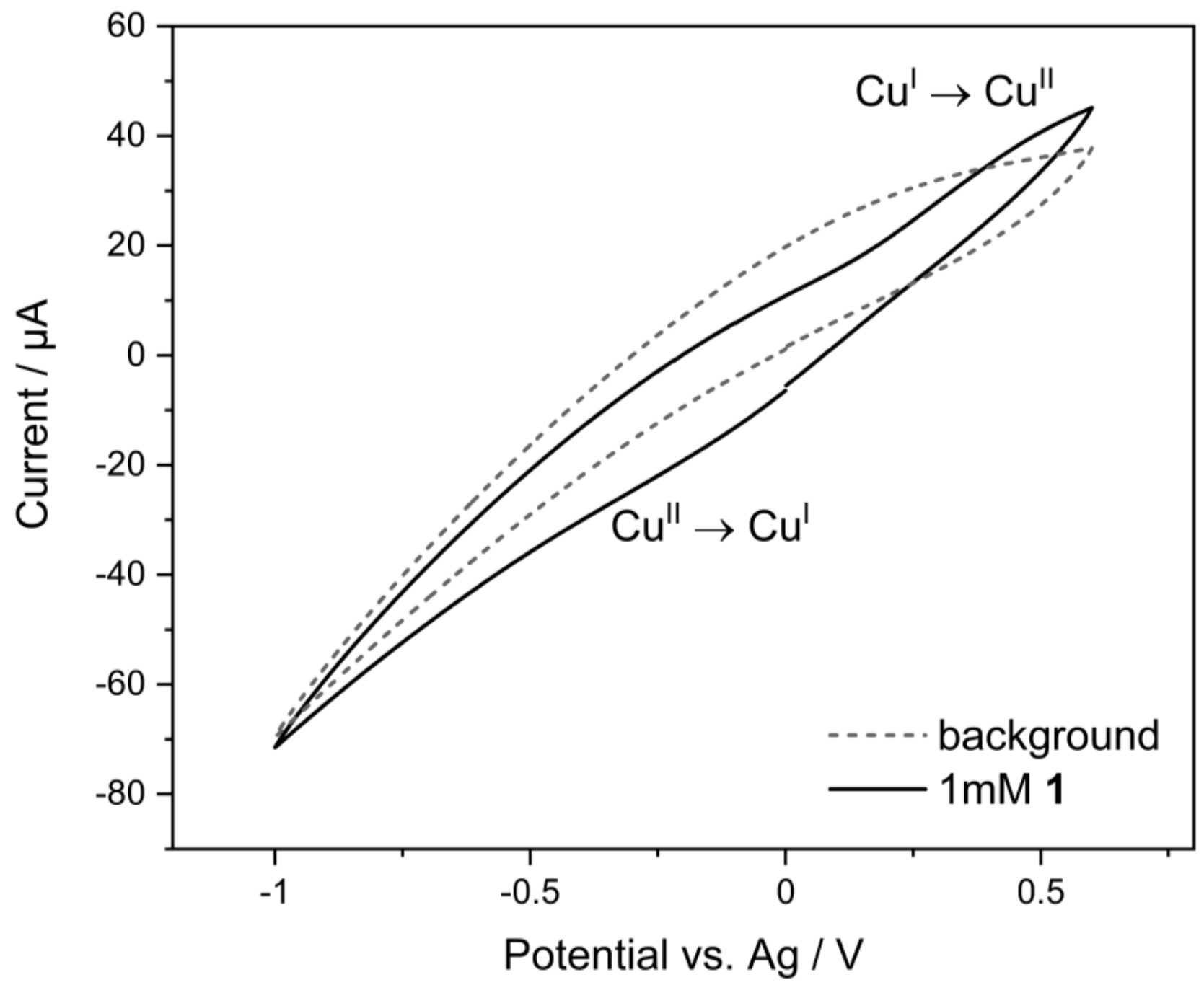

Fig. S20 Cyclic voltammogram of **1** (1 mM) within the SEC-UV-vis set-up in H_2O:DMSO (10:1) using KCl (0.1 M) as the supporting electrolyte.

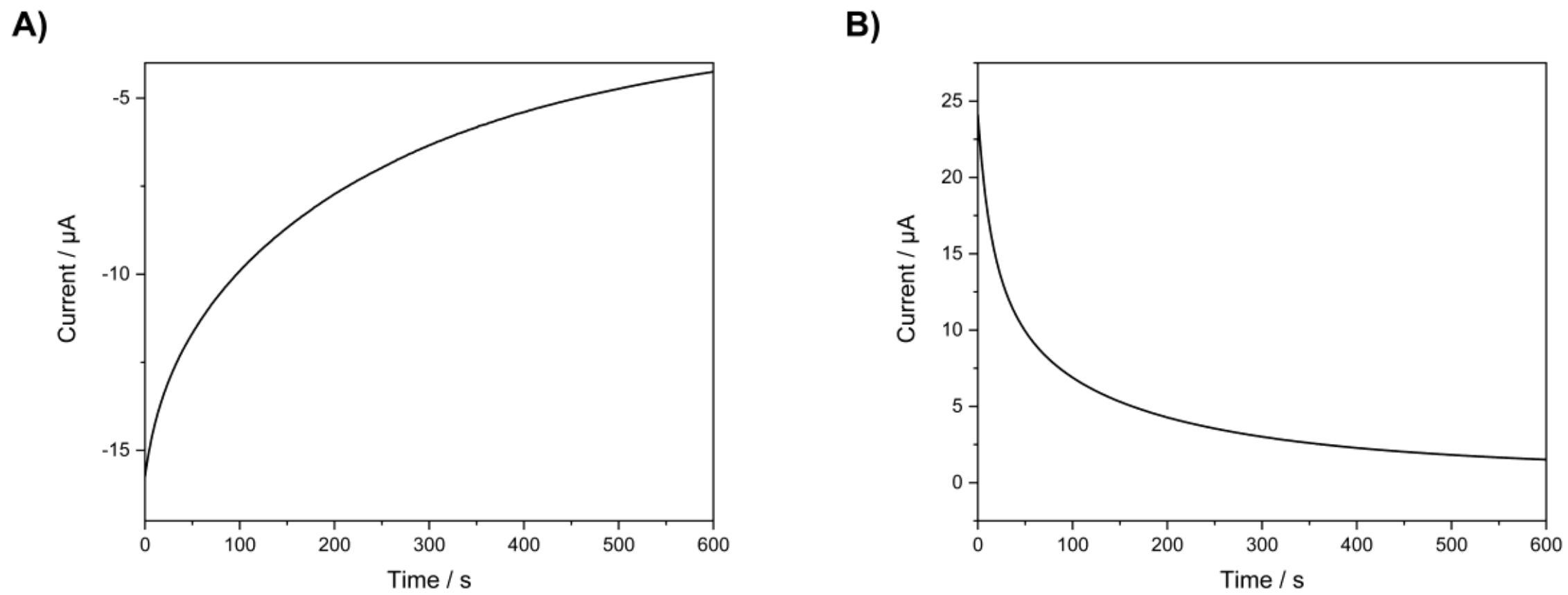

Fig. S21 Controlled potential coulometry of **1** (1 mM) in H_2O:DMSO (10:1) with KCl (0.1 M) for 10 min at -0.2 V vs. Ag (A) and 0.5 V vs. Ag (B) within the SEC-UV-vis set-up.

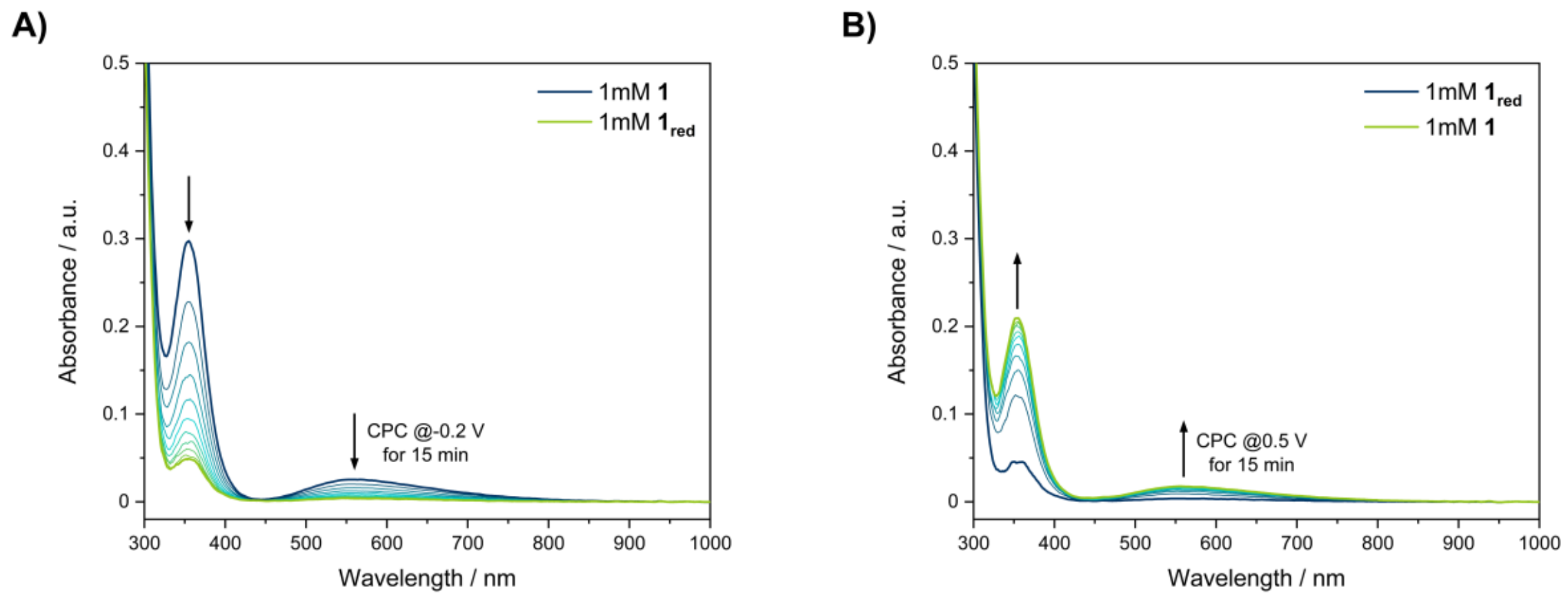

Fig. S22 UV-vis spectra recorded during the electrolysis of **1** (1 mM) in H_2O:DMSO (10:1) at a potential of -0.2 V vs. Ag (1 min interval) (A). To the same solution afterwards a potential of 0.5 V vs. Ag was applied, and UV-vis spectra were recorded in 1 min interval over 15 min (B).

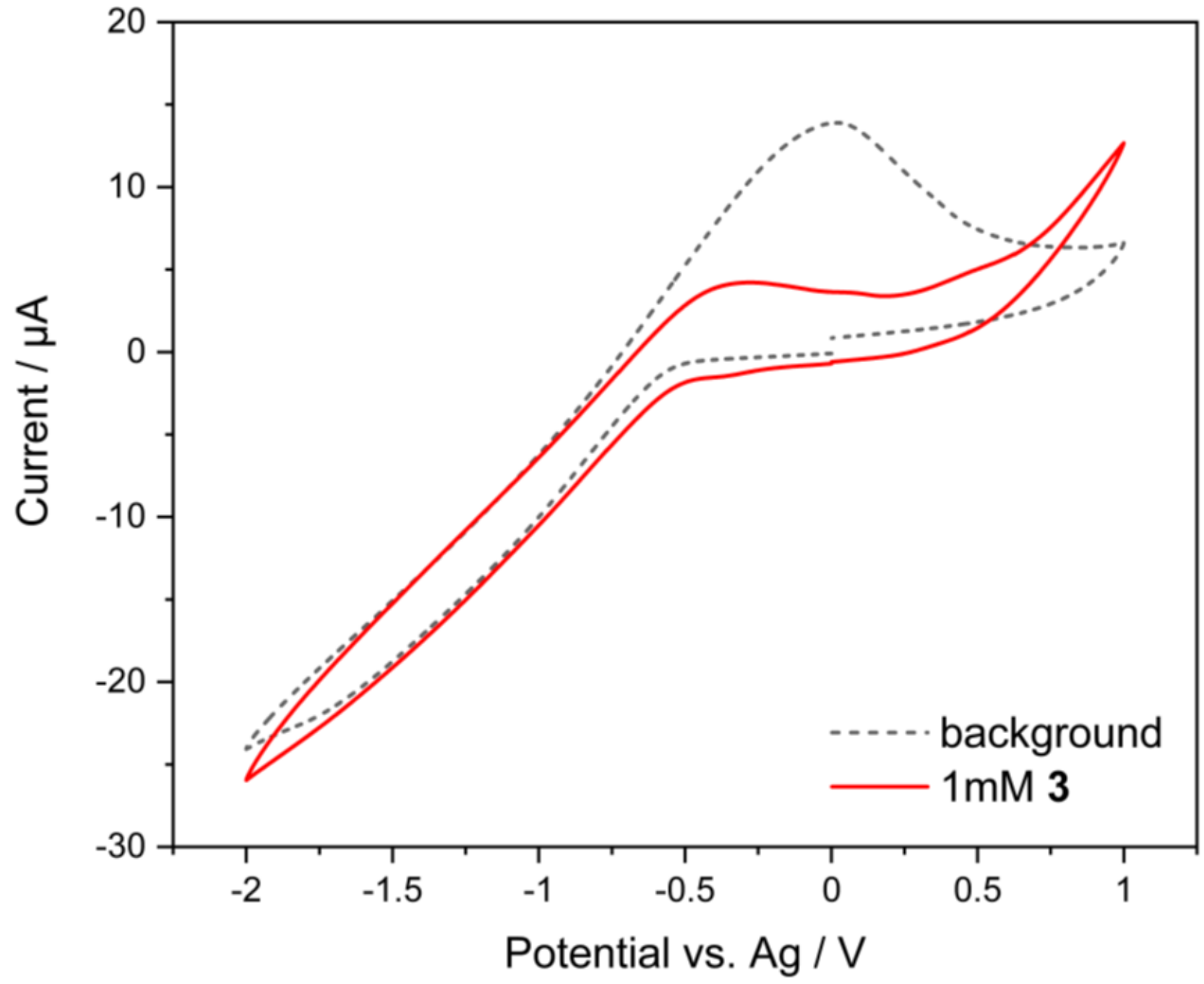

Fig. S23 Cyclic voltammogram of **3** (1 mM) within the SEC-UV-vis set-up in DMSO using $TBAPF_6$ (0.1 M) as the supporting electrolyte.

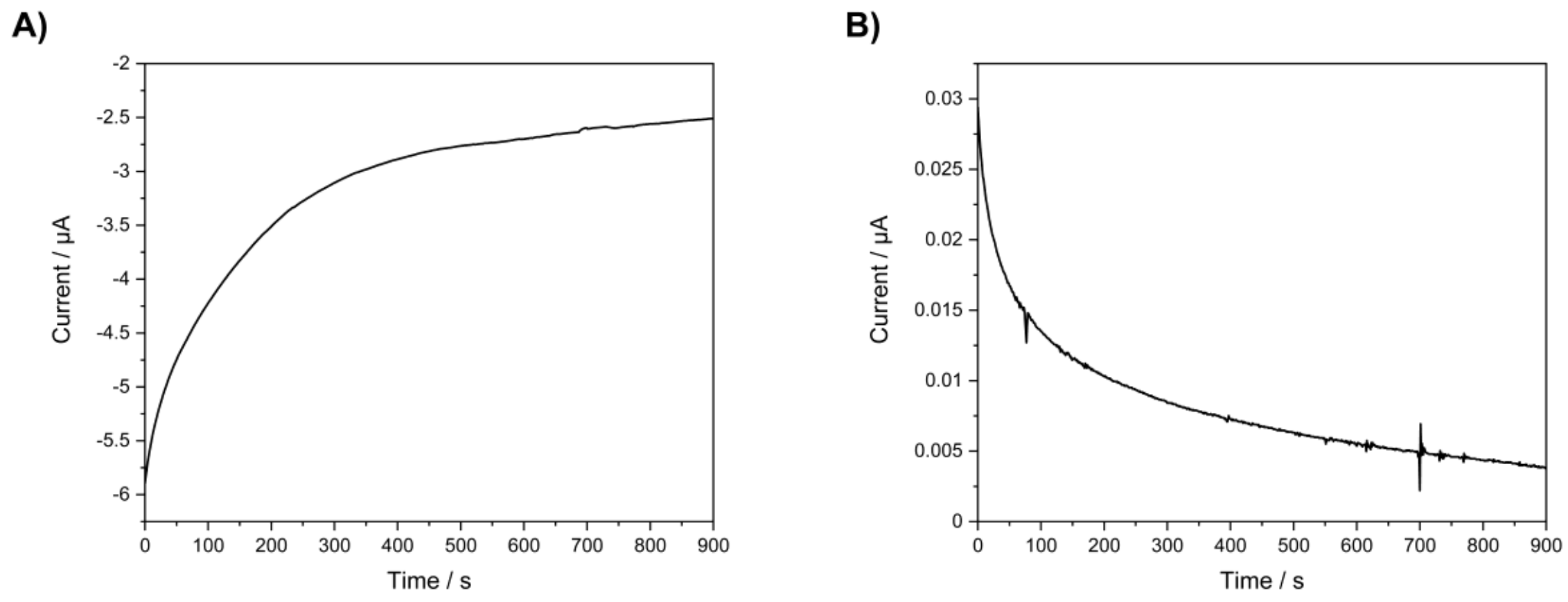

Fig. S24 Controlled potential coulometry of **3** (1 mM) in DMSO with $TBAPF_6$ (0.1 M) for 15 min at -0.8 V vs. Ag (A) and 0 V vs. Ag (B) within the SEC-UV-vis set-up.

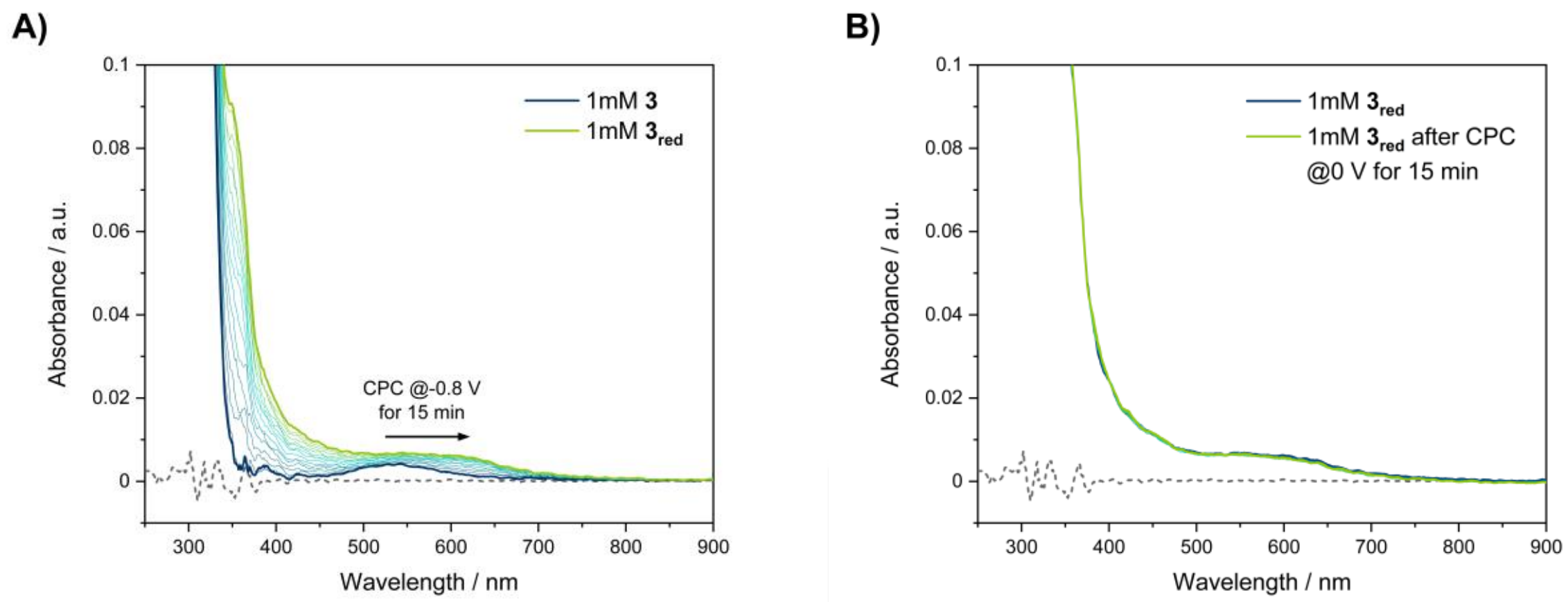

Fig. S25 UV-vis spectra recorded during the electrolysis of **3** (1 mM) in DMSO at a potential of -0.8 V vs. Ag (1 min interval) (A). To the same solution afterwards a potential of 0 V vs. Ag was applied, and UV-vis spectra were recorded in 1 min interval over 15 min (B).

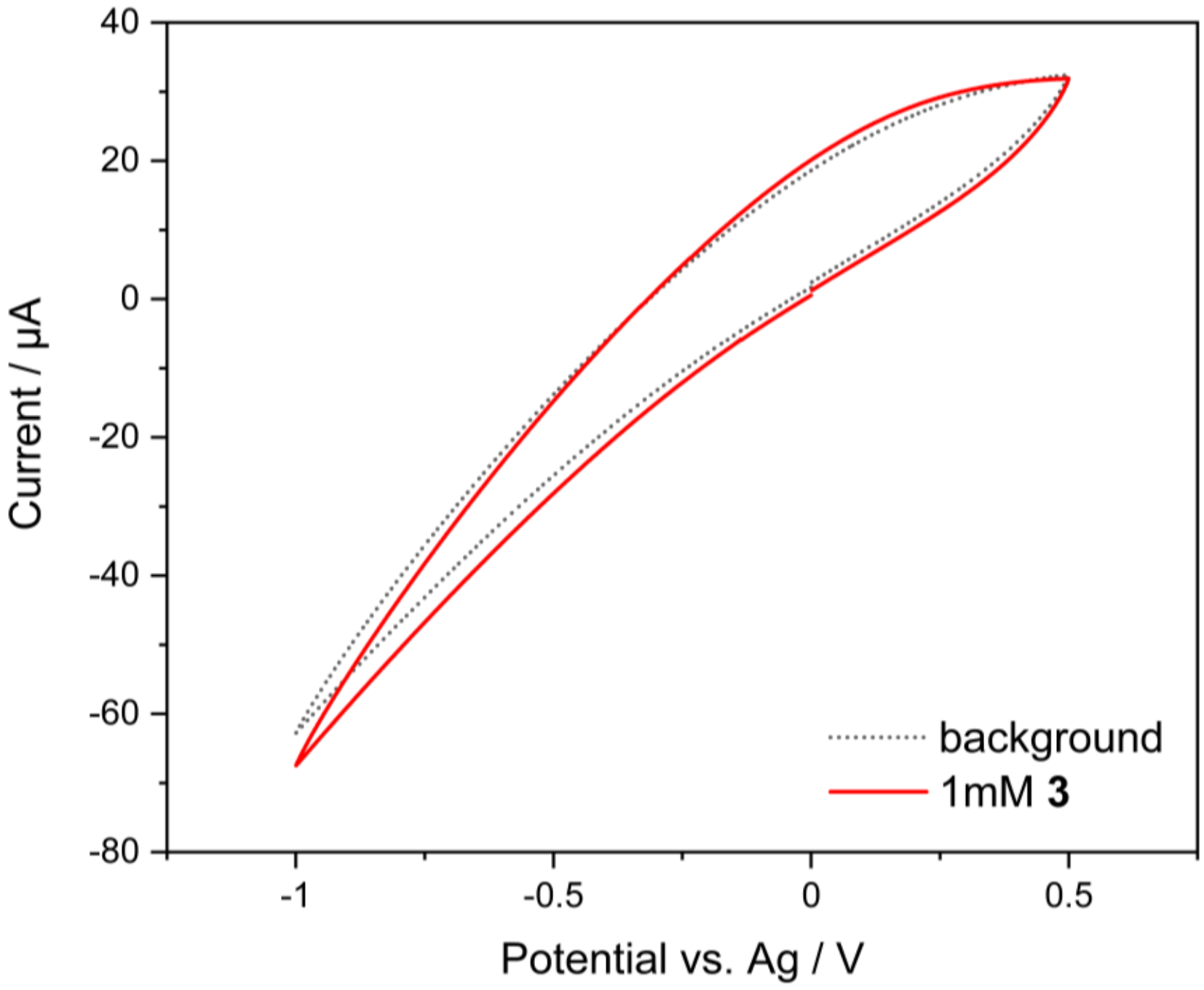

Fig. S26 Cyclic voltammogram of **3** (1 mM) within the SEC-UV-vis set-up in H_2O:DMSO (10:1) using KCl (0.1 M) as the supporting electrolyte.

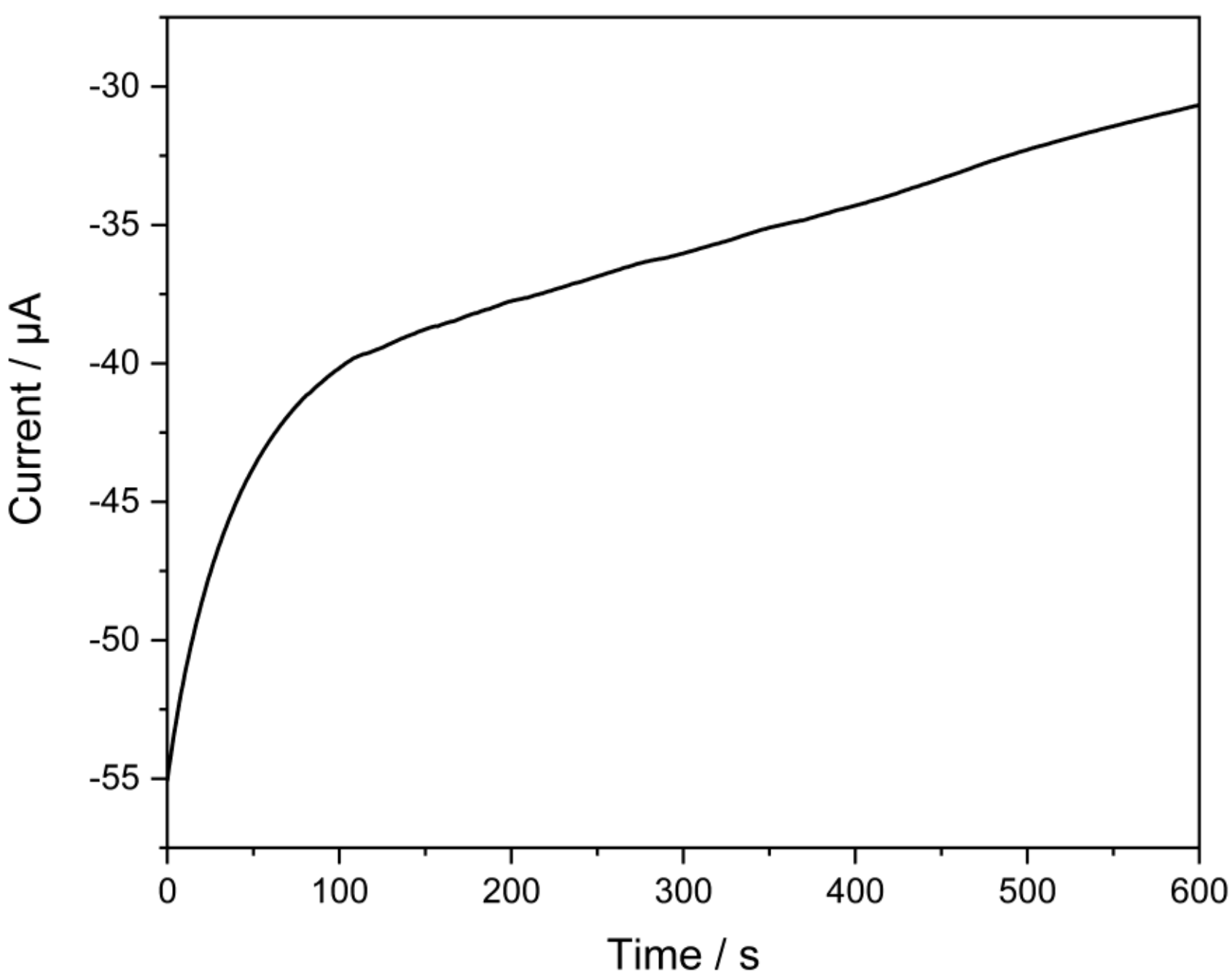

Fig. S27 Controlled potential coulometry of **3** (1 mM) in H_2O:DMSO (10:1) with KCl (0.1 M) for 10 min at -1.0 V vs. Ag within the SEC-UV-vis set-up.

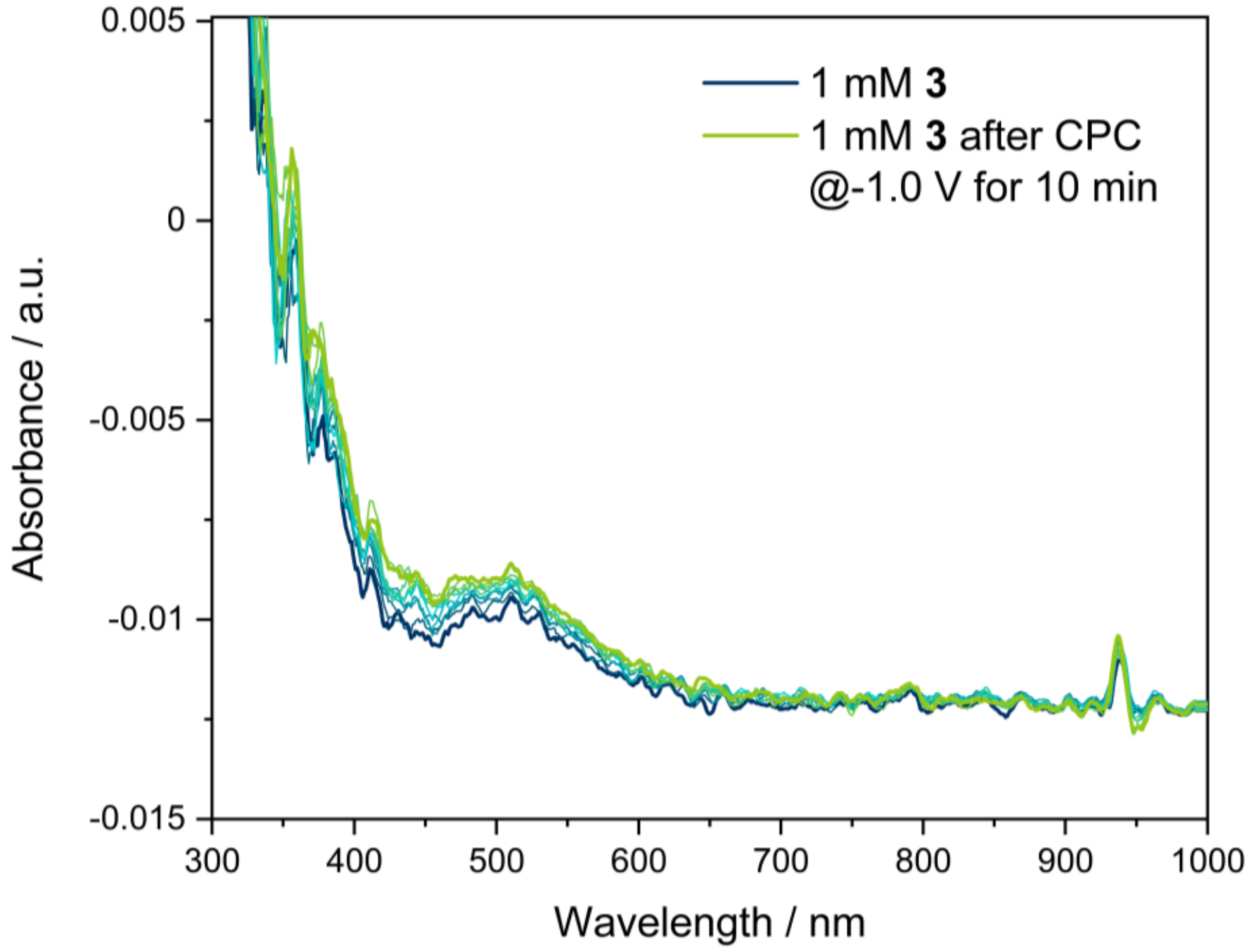

Fig. S28 UV-vis spectra recorded during the electrolysis of **3** (1 mM) in H_2O:DMSO (10:1) at a potential of -1.0 V vs. Ag (1 min interval) over 10 min.

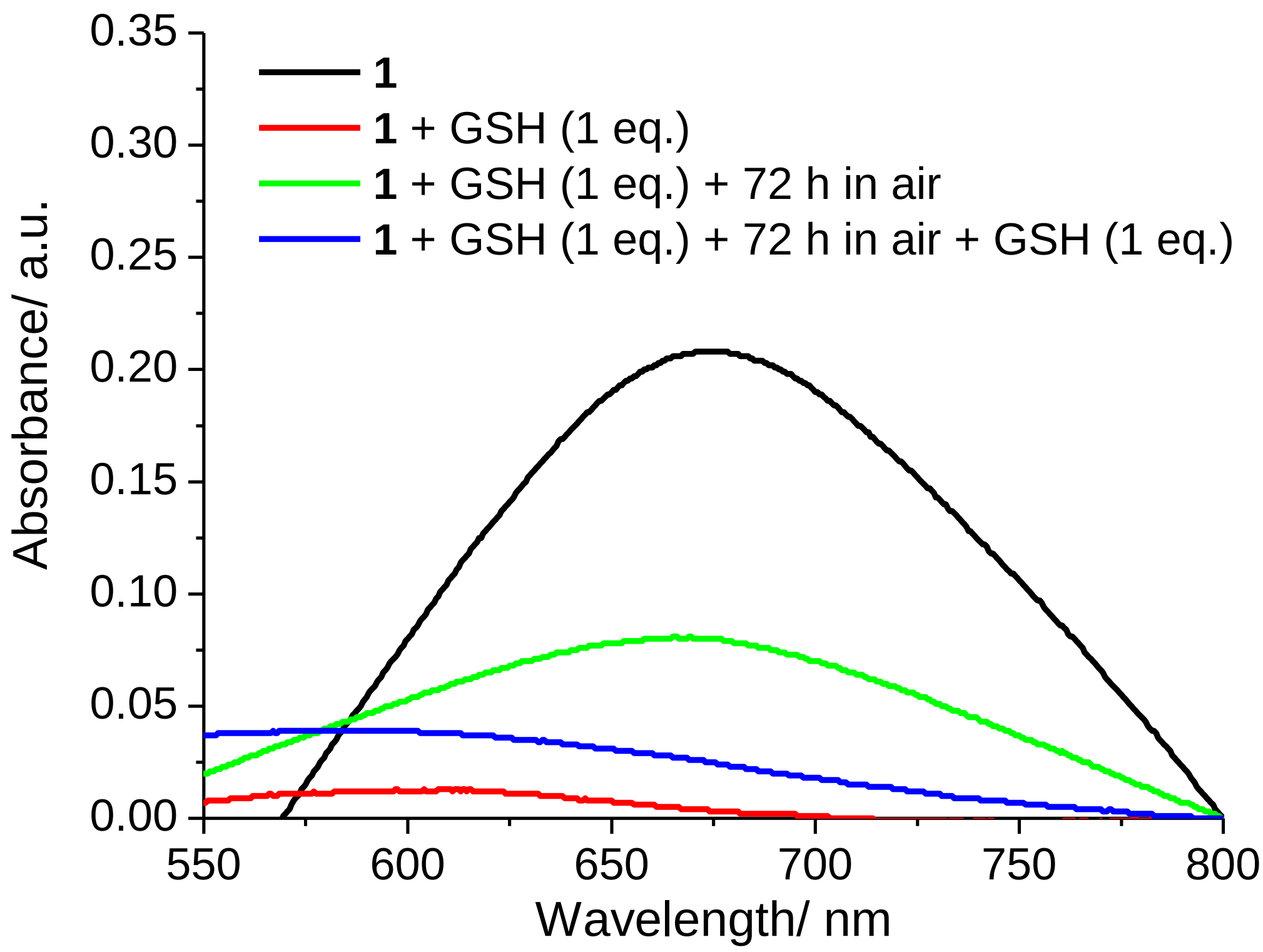

Fig. S29 UV-vis spectra of **1** (1 mM) in DMSO, upon addition of glutathione (1 mM, 1 equivalence), exposure to air for 72 h, and further addition of glutathione (1 mM, 1 equivalence).

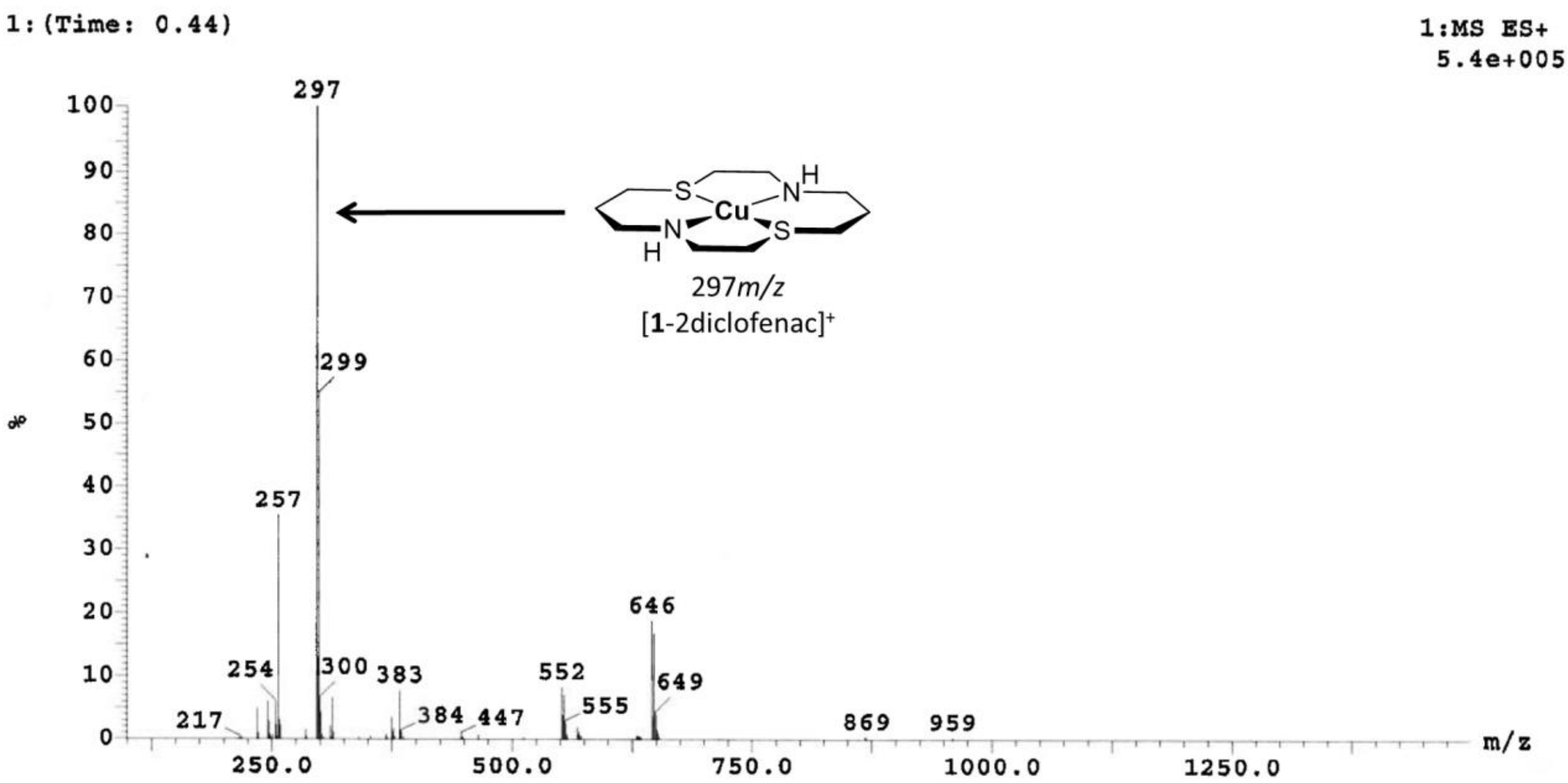

Fig. S30 ESI mass spectra (positive mode) of **1** (1 mM) in DMSO after addition of glutathione (1 mM, 1 equivalence), exposure to air for 72 h, and further addition of glutathione (1 mM, 1 equivalence).

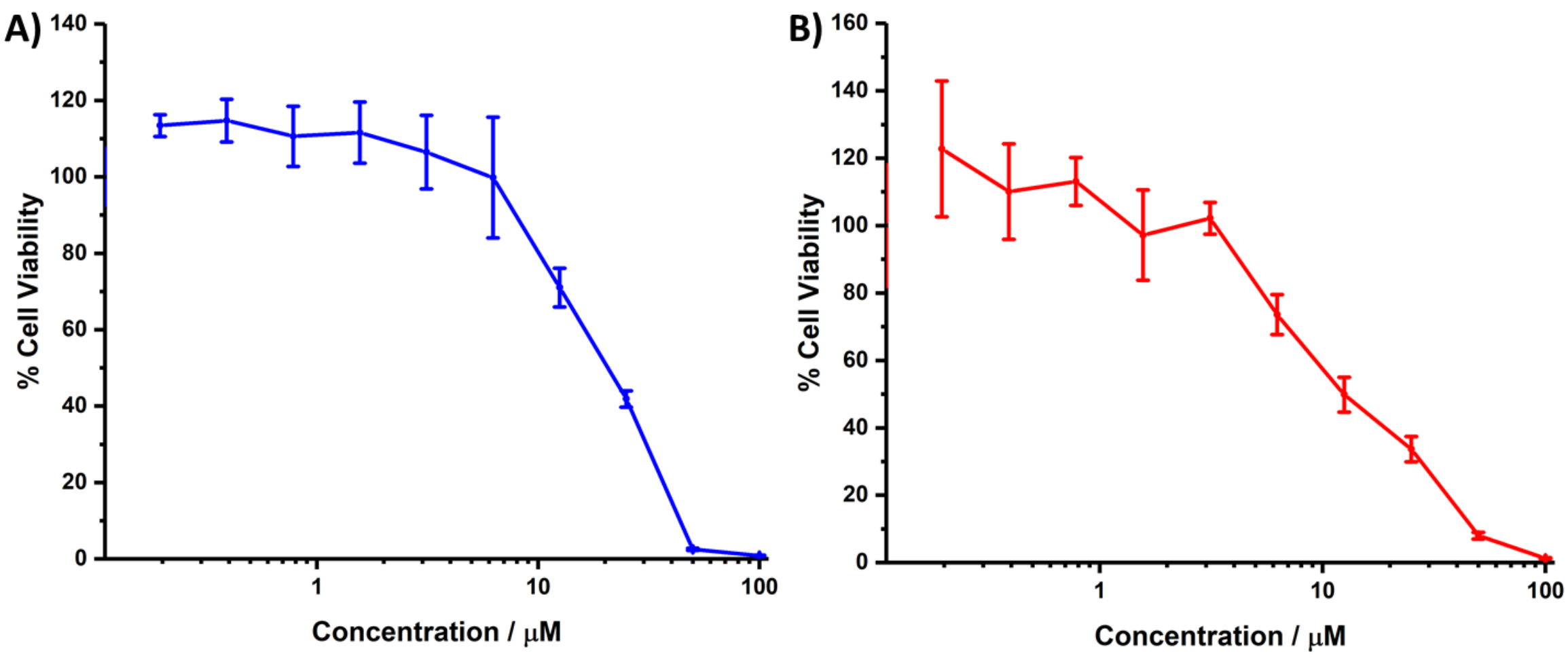

Fig. S31 Representative dose-response curves for the treatment of (A) HMLER or (B) HMLER-shEcad cells with **1** after 72 h incubation.

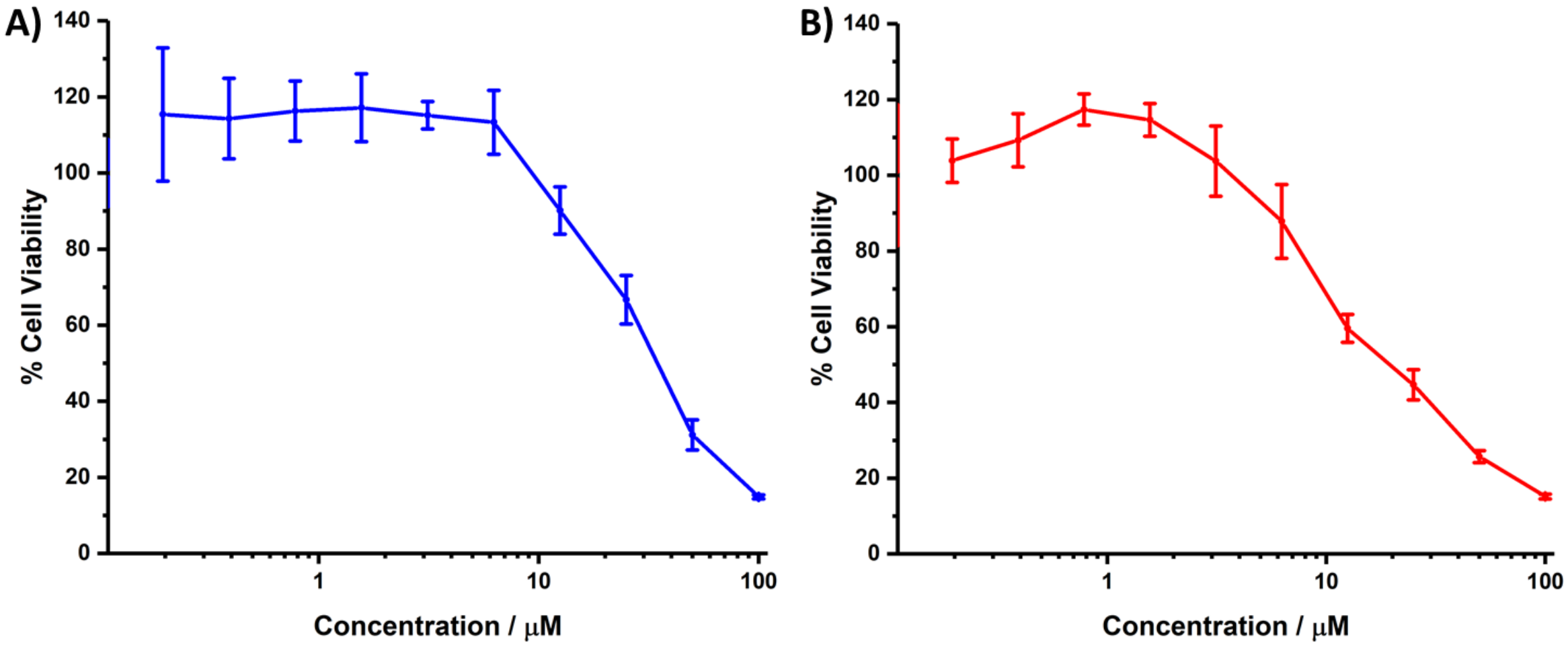

Fig. S32 Representative dose-response curves for the treatment of (A) HMLER or (B) HMLER-shEcad cells with **3** after 72 h incubation.

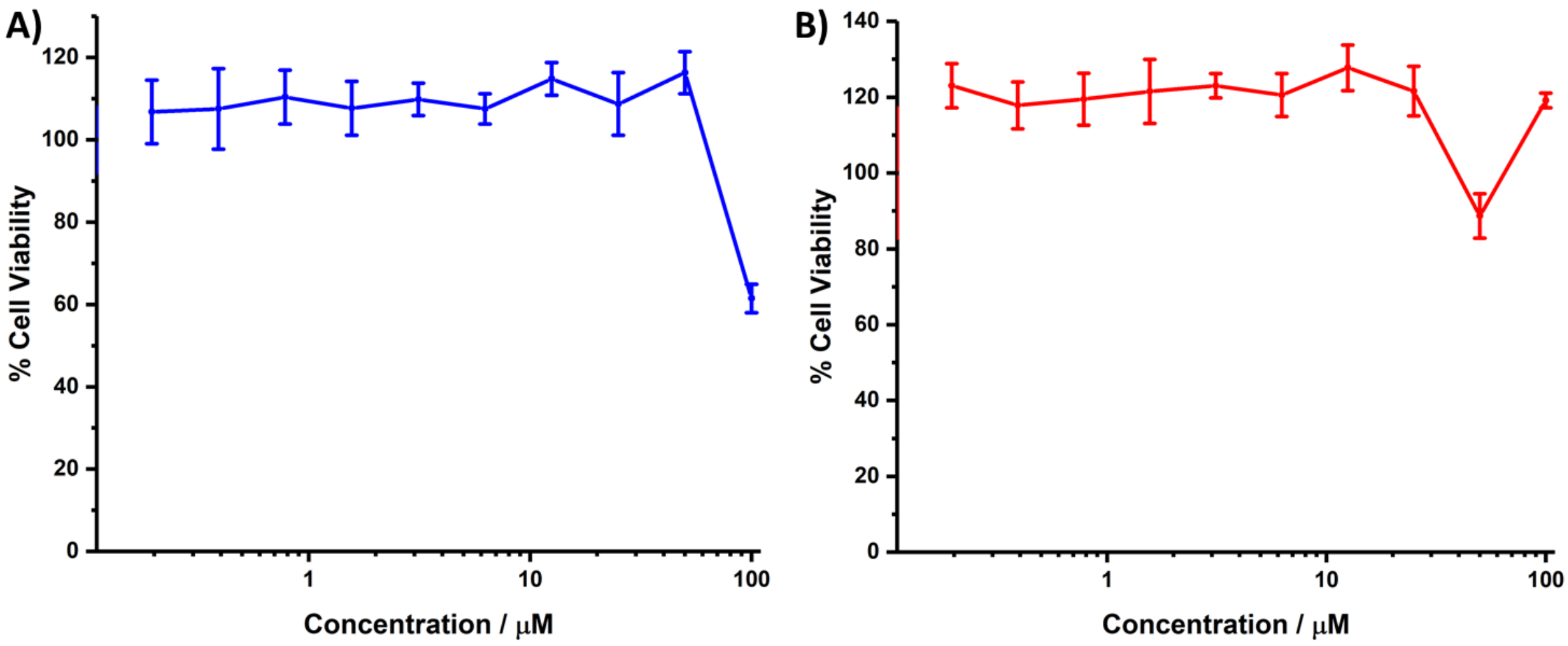

Fig. S33 Representative dose-response curves for the treatment of (A) HMLER or (B) HMLER-shEcad cells with **2** after 72 h incubation.

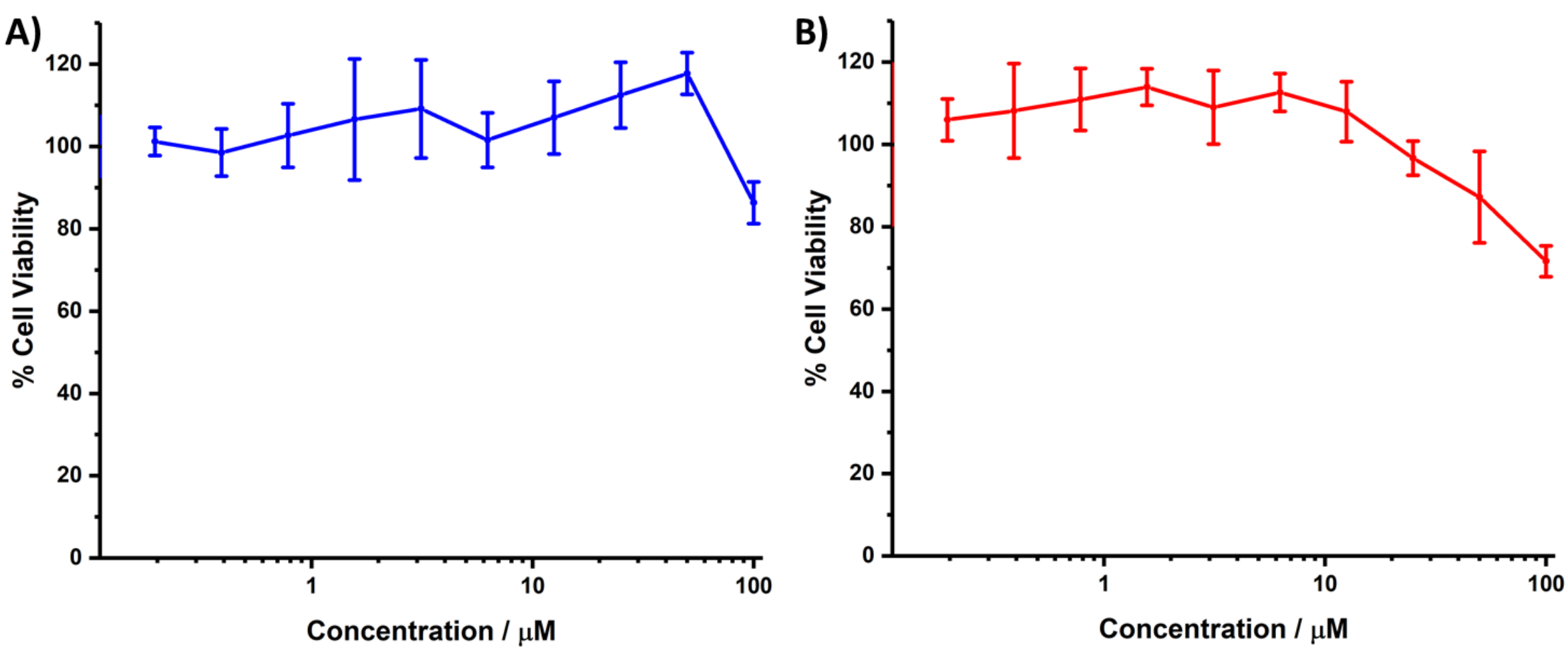

Fig. S34 Representative dose-response curves for the treatment of (A) HMLER or (B) HMLER-shEcad cells with **4** after 72 h incubation.

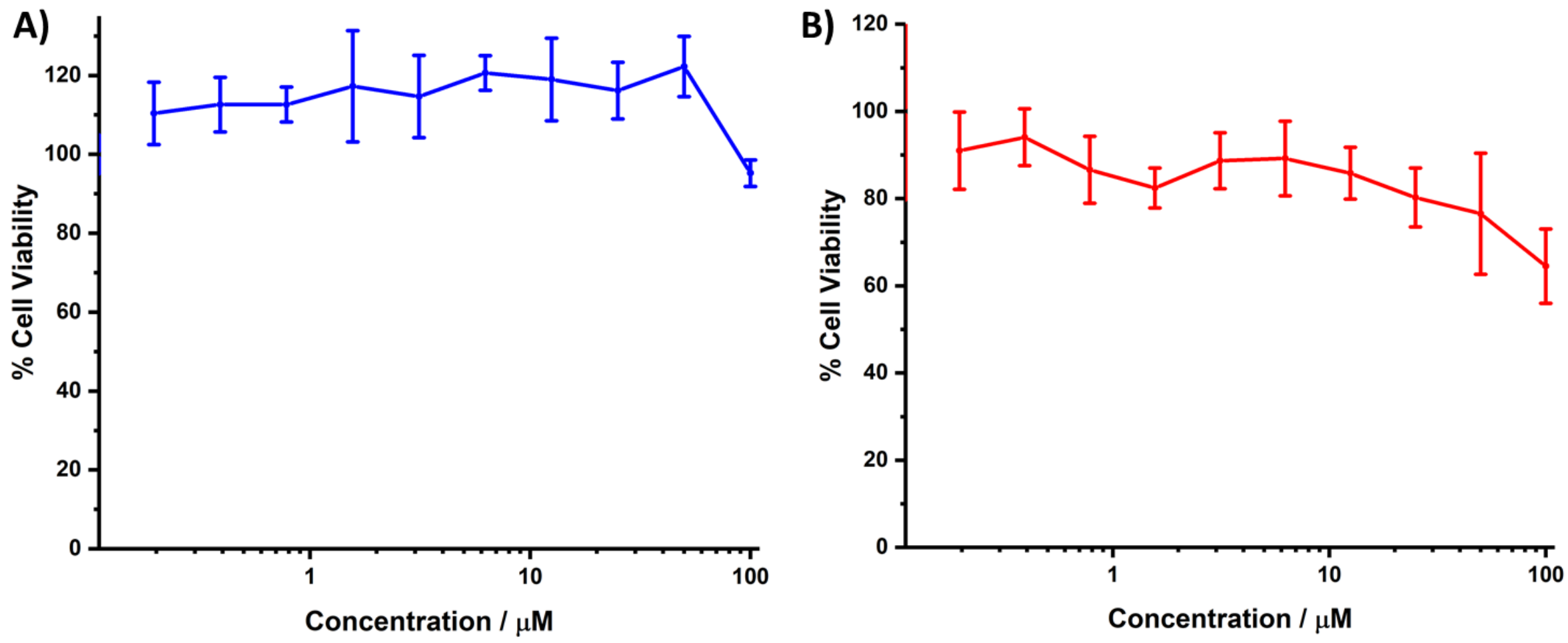

Fig. S35 Representative dose-response curves for the treatment of (A) HMLER or (B) HMLER-shEcad cells with dithiacyclam after 72 h incubation.

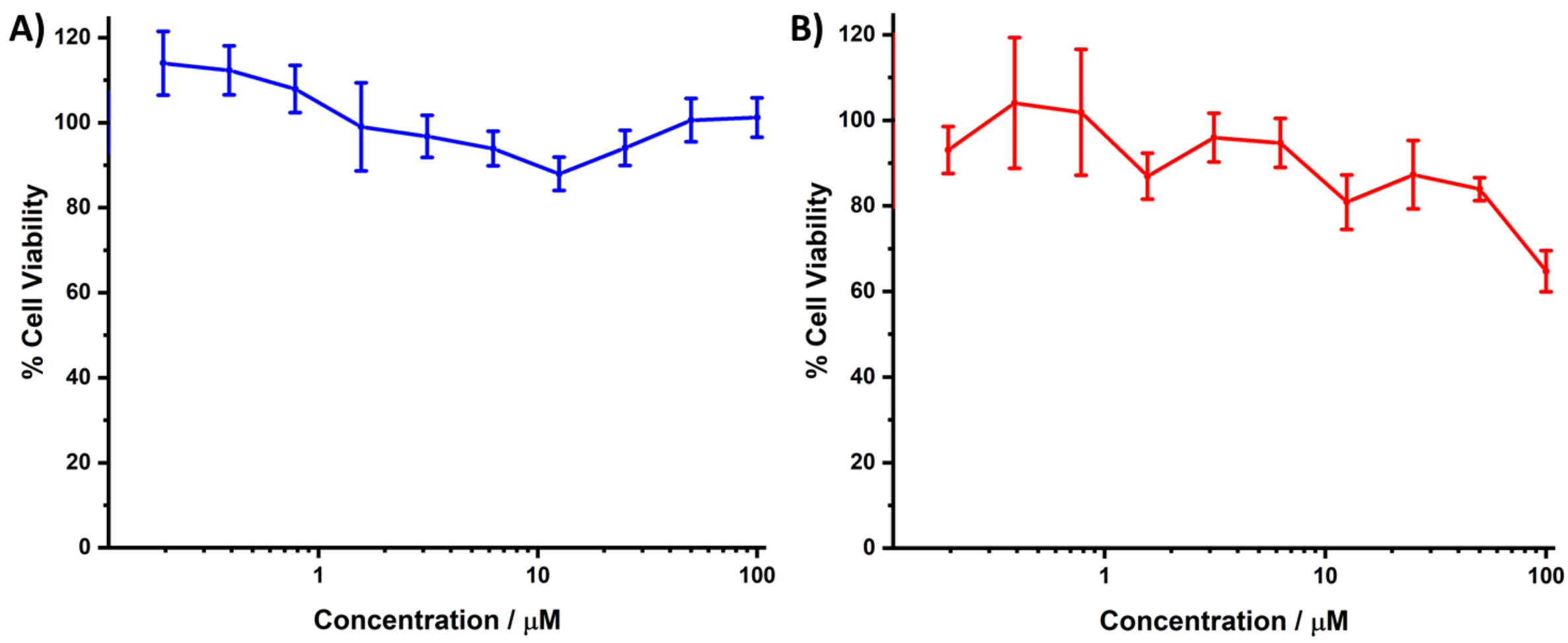

Fig. S36 Representative dose-response curves for the treatment of (A) HMLER or (B) HMLER-shEcad cells with cyclam after 72 h incubation.

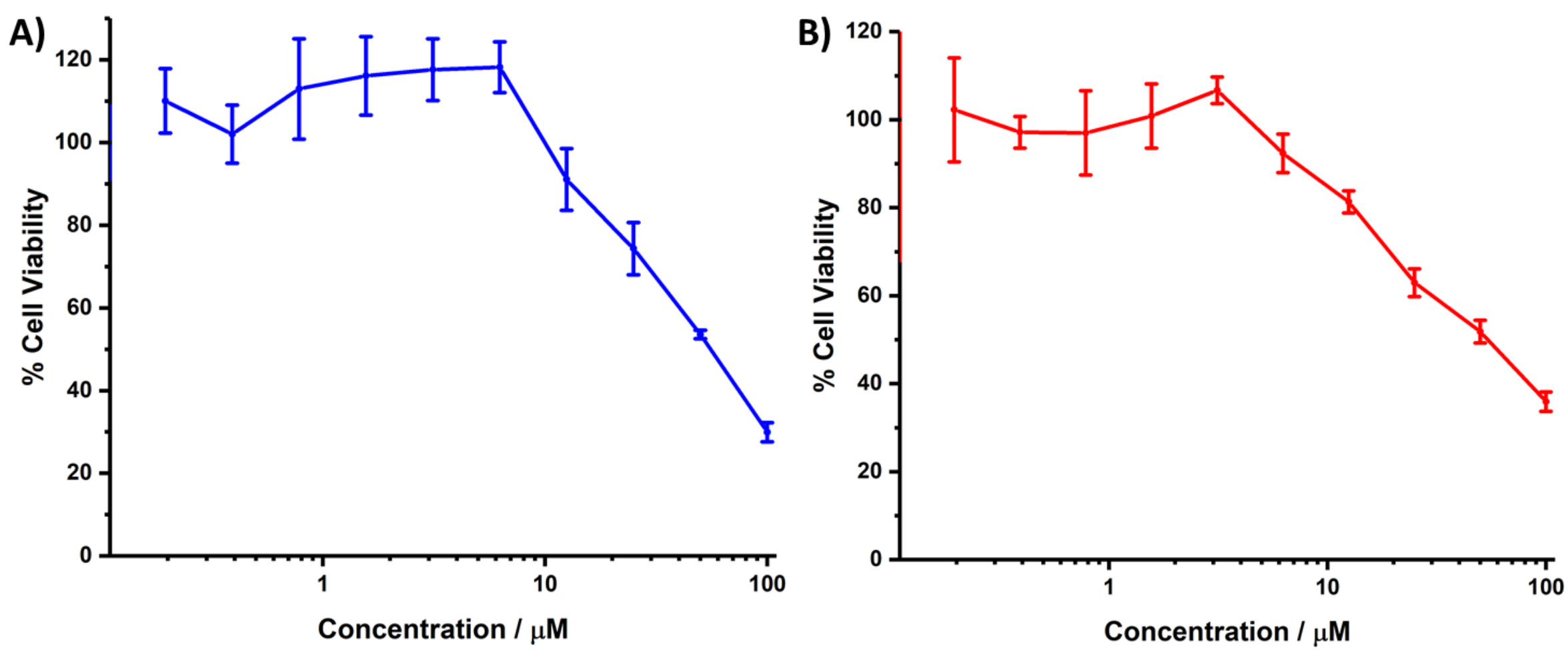

Fig. S37 Representative dose-response curves for the treatment of (A) HMLER or (B) HMLER-shEcad cells with sodium diclofenac after 72 h incubation.

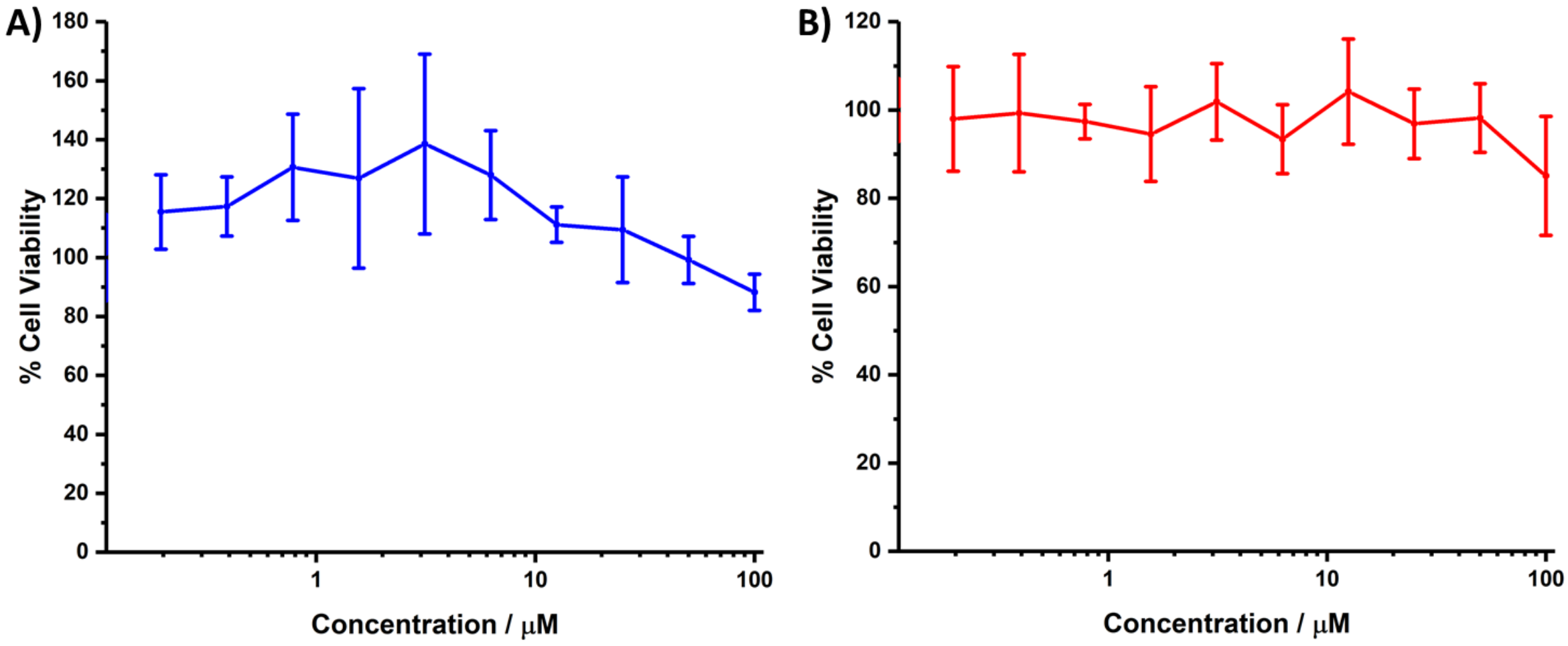

Fig. S38 Representative dose-response curves for the treatment of (A) HMLER or (B) HMLER-shEcad cells with **5** after 72 h incubation.

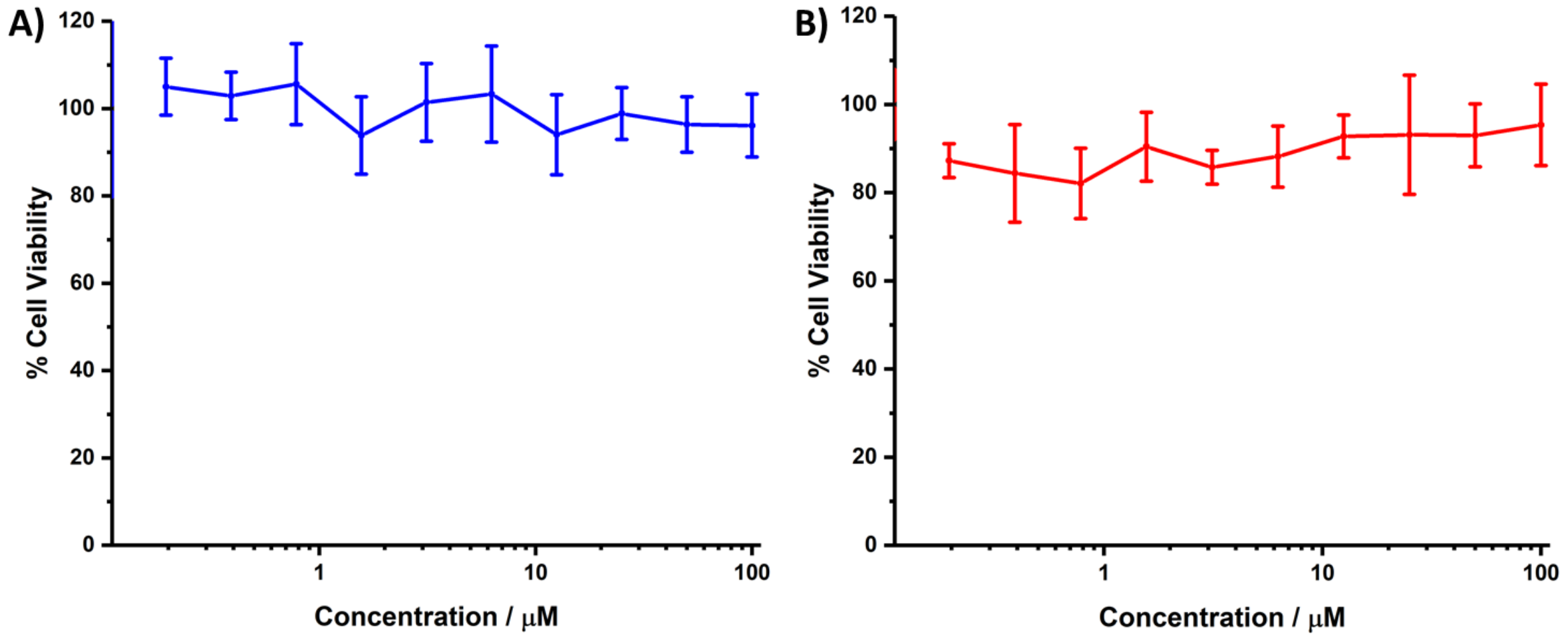

Fig. S39 Representative dose-response curves for the treatment of (A) HMLER or (B) HMLER-shEcad cells with **6** after 72 h incubation.

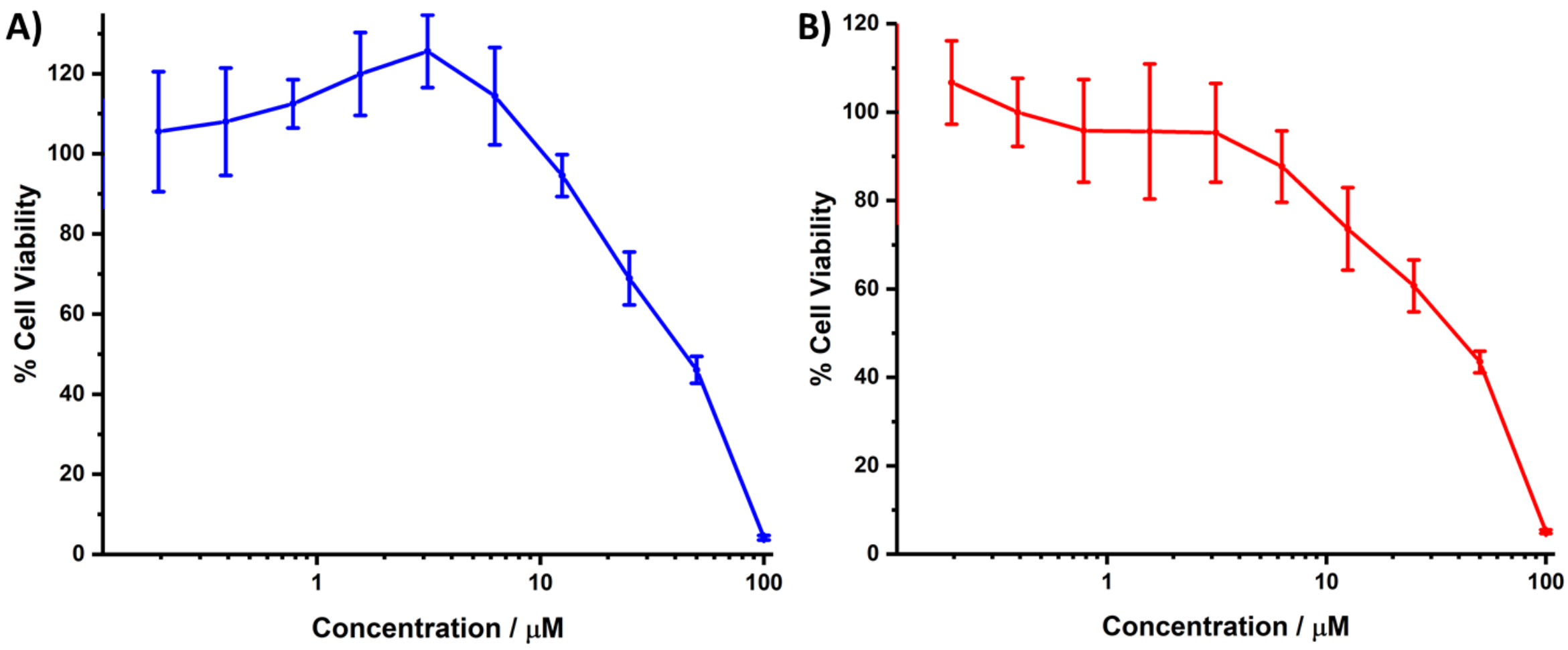

Fig. S40 Representative dose-response curves for the treatment of (A) HMLER or (B) HMLER-shEcad cells with **5** and sodium diclofenac after 72 h incubation.

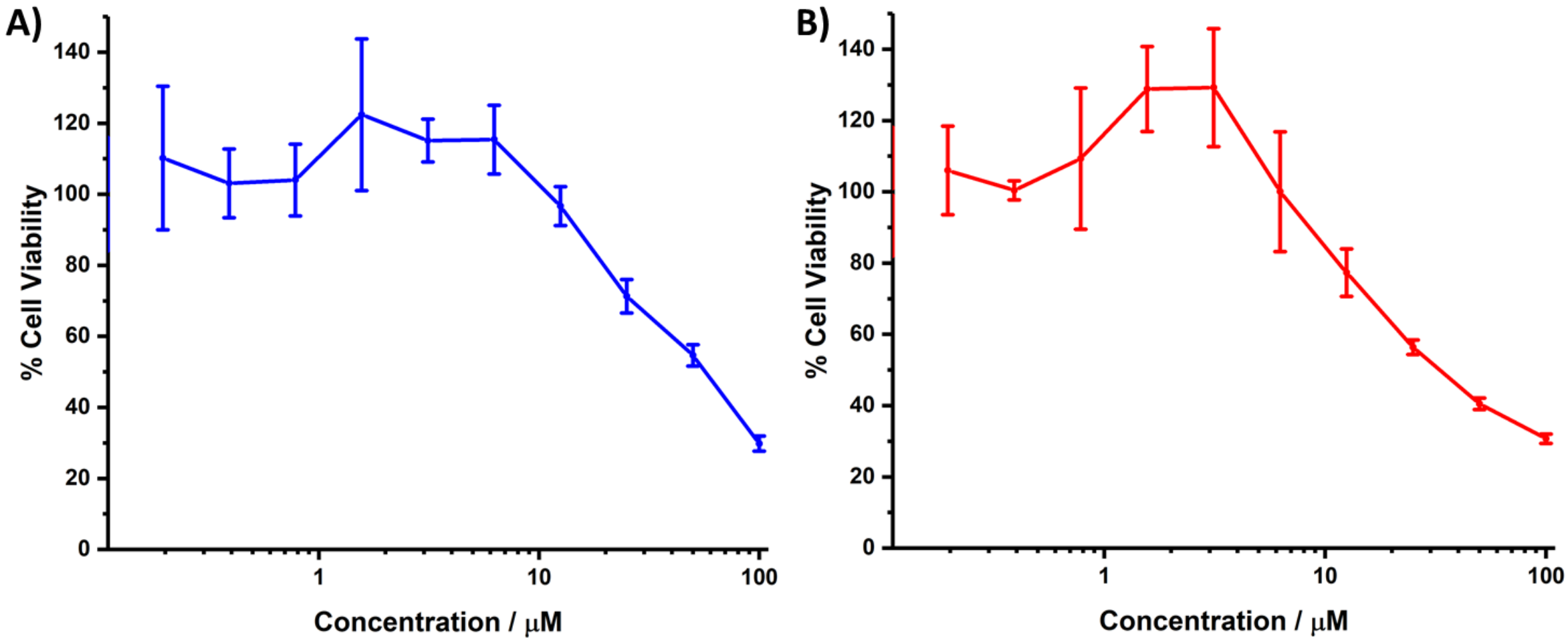

Fig. S41 Representative dose-response curves for the treatment of (A) HMLER or (B) HMLER-shEcad cells with **6** and sodium diclofenac after 72 h incubation.

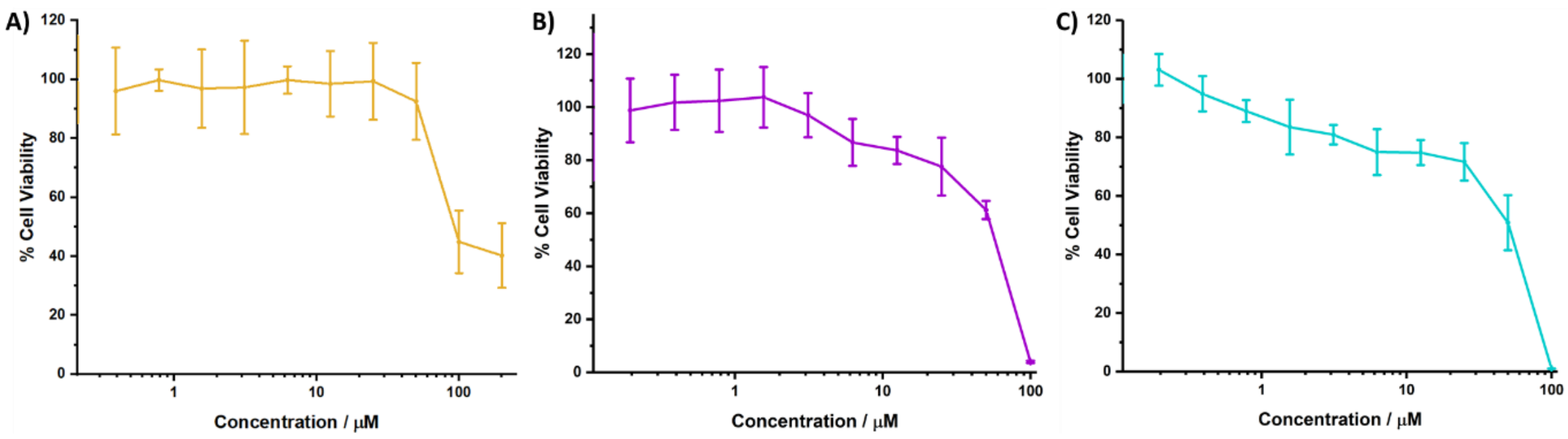

Fig. S42 Representative dose-response curves for the treatment of (A) MCF10A, (B) BEAS-2B, or (C) HEK293 cells with **1** after 72 h incubation.

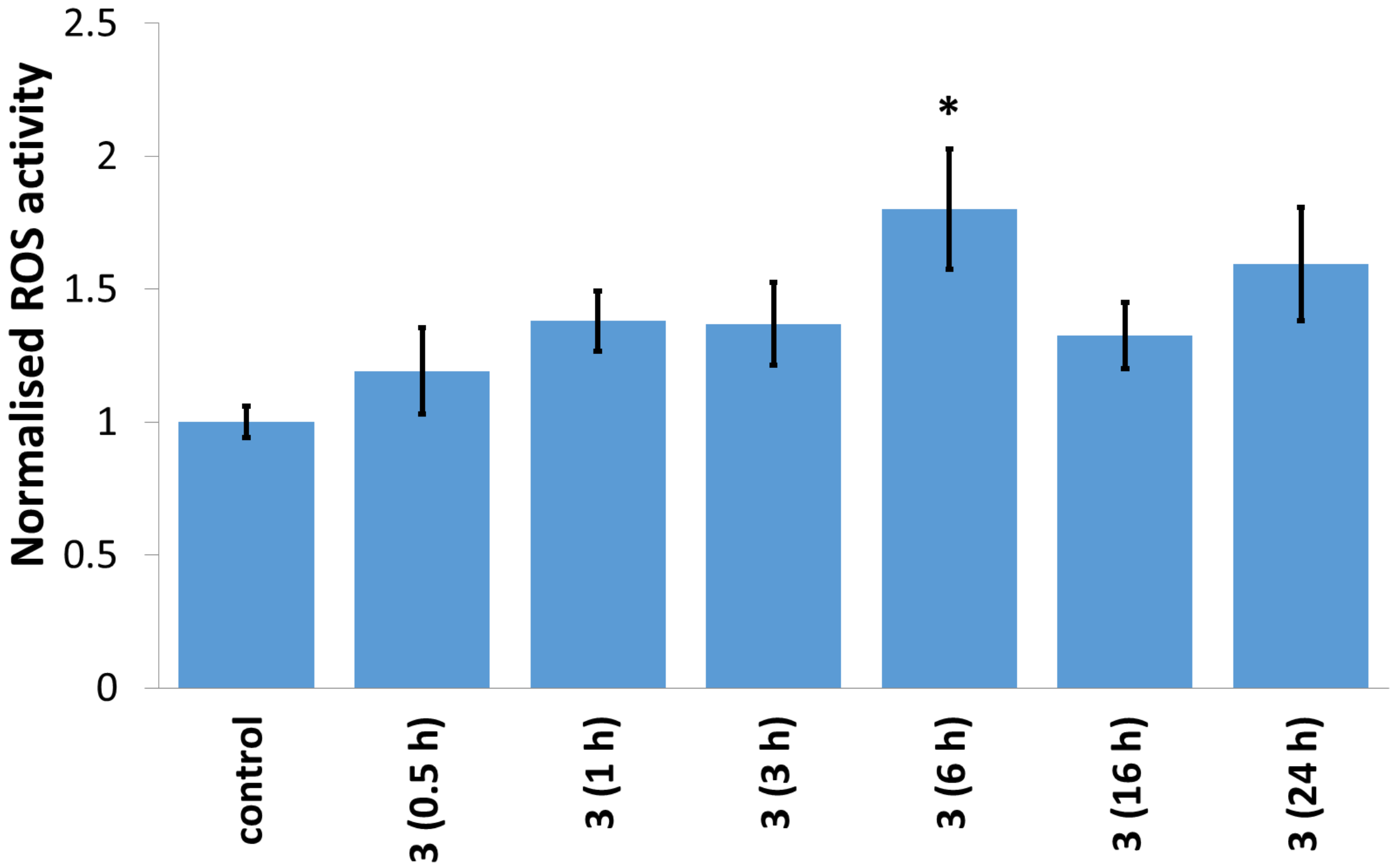

Fig. S43 Normalised ROS activity in untreated HMLER-shEcad cells (control) and HMLER-shEcad cells treated with **3** (IC_{50} value × 2 for 0.5, 1, 3, 6, 16, and 24 h).

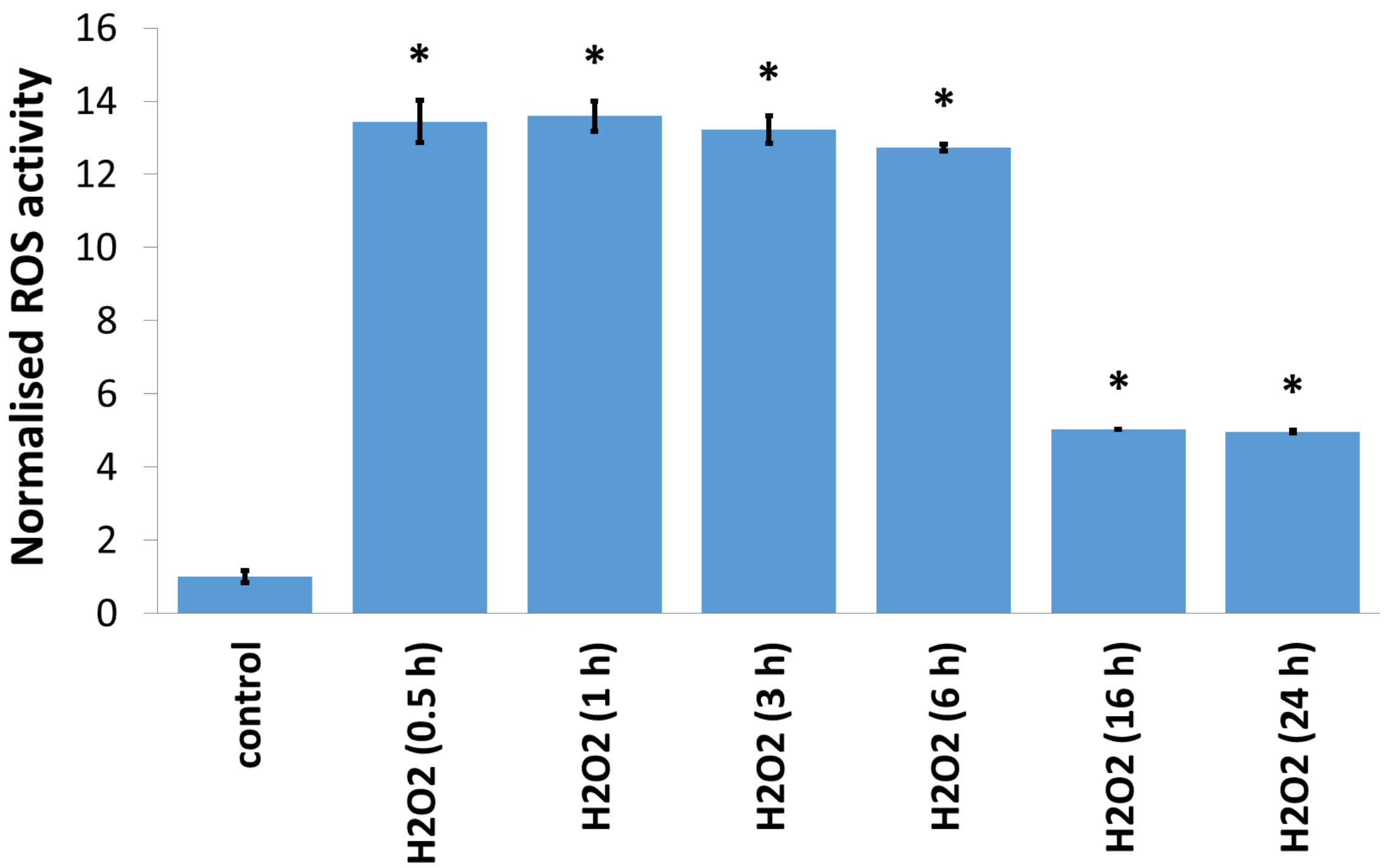

Fig. S44 Normalised ROS activity in untreated HMLER-shEcad cells (control) and HMLER-shEcad cells treated with H_2O_2 (150 µM for 0.5, 1, 3, 6, 16, and 24 h).

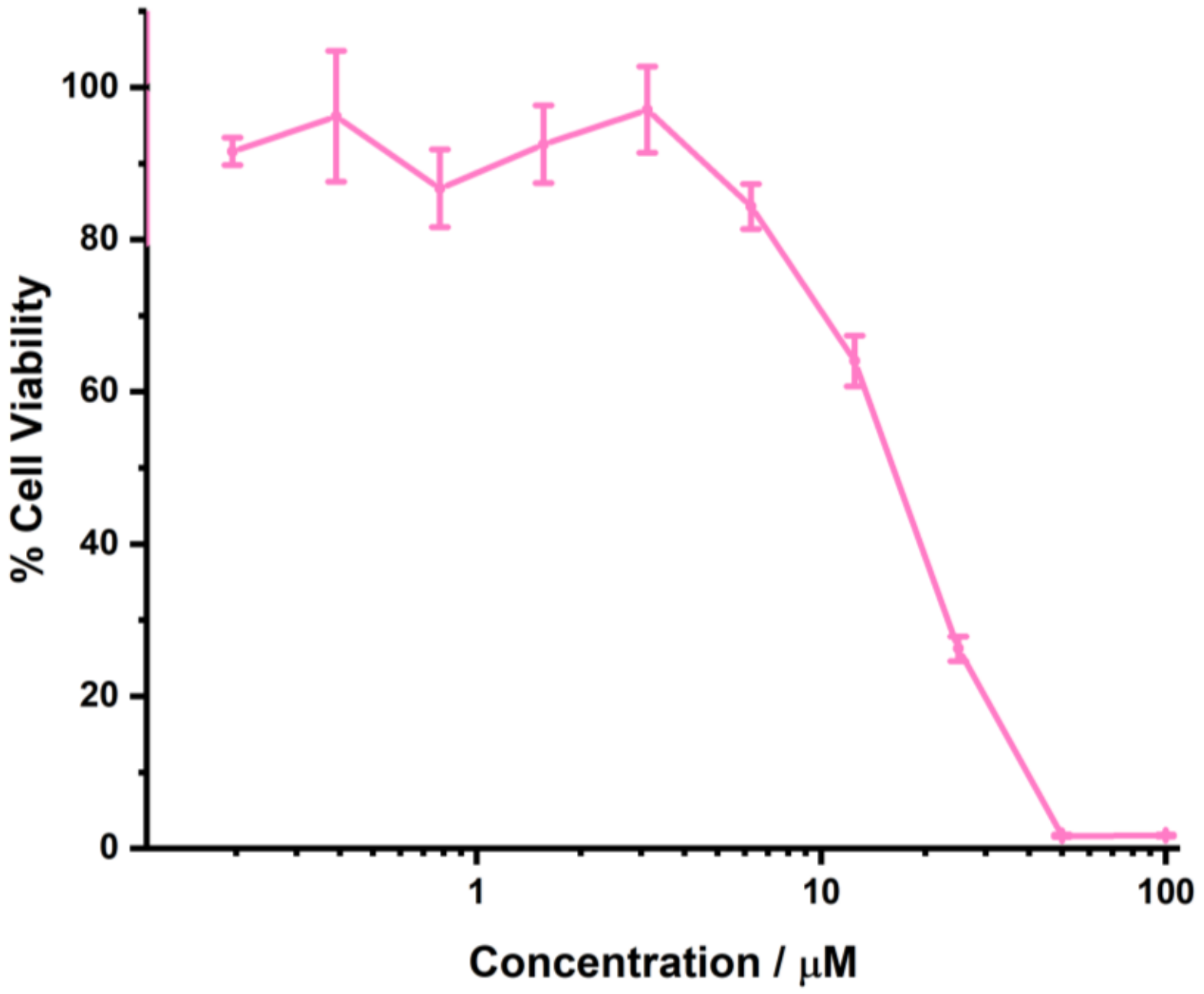

Fig. S45 Representative dose-response curve of **1** against HMLER-shEcad cells in the presence of *N*-acetylcysteine (2.5 mM) after 72 h incubation.

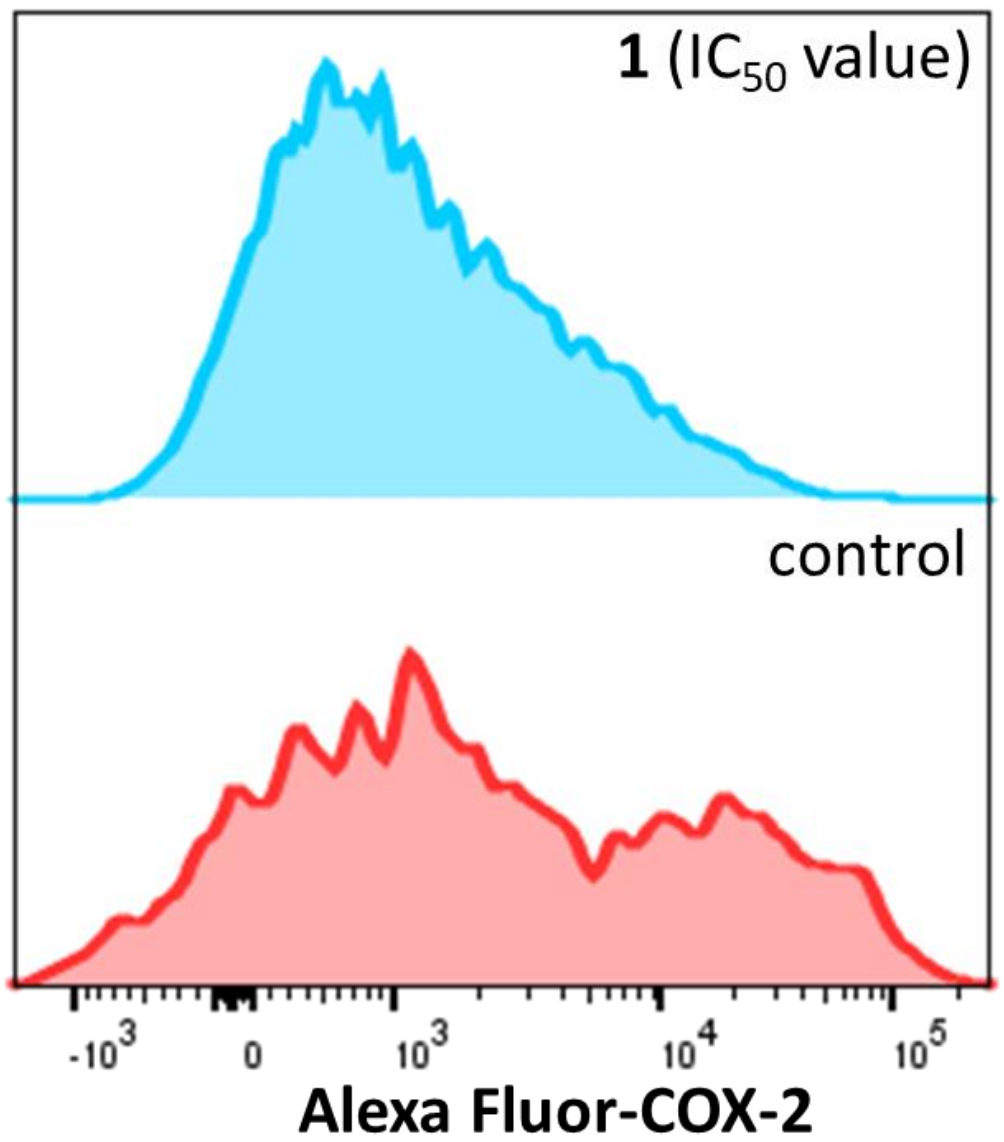

Fig. S46 Representative histograms displaying the green fluorescence emitted by anti-COX-2 Alexa Fluor 488 nm antibody-stained HMLER-shEcad cells treated with LPS (2.5 μg/ L) for 24 h (red) followed by 48 h in media containing **1** (IC_{50} value, blue).

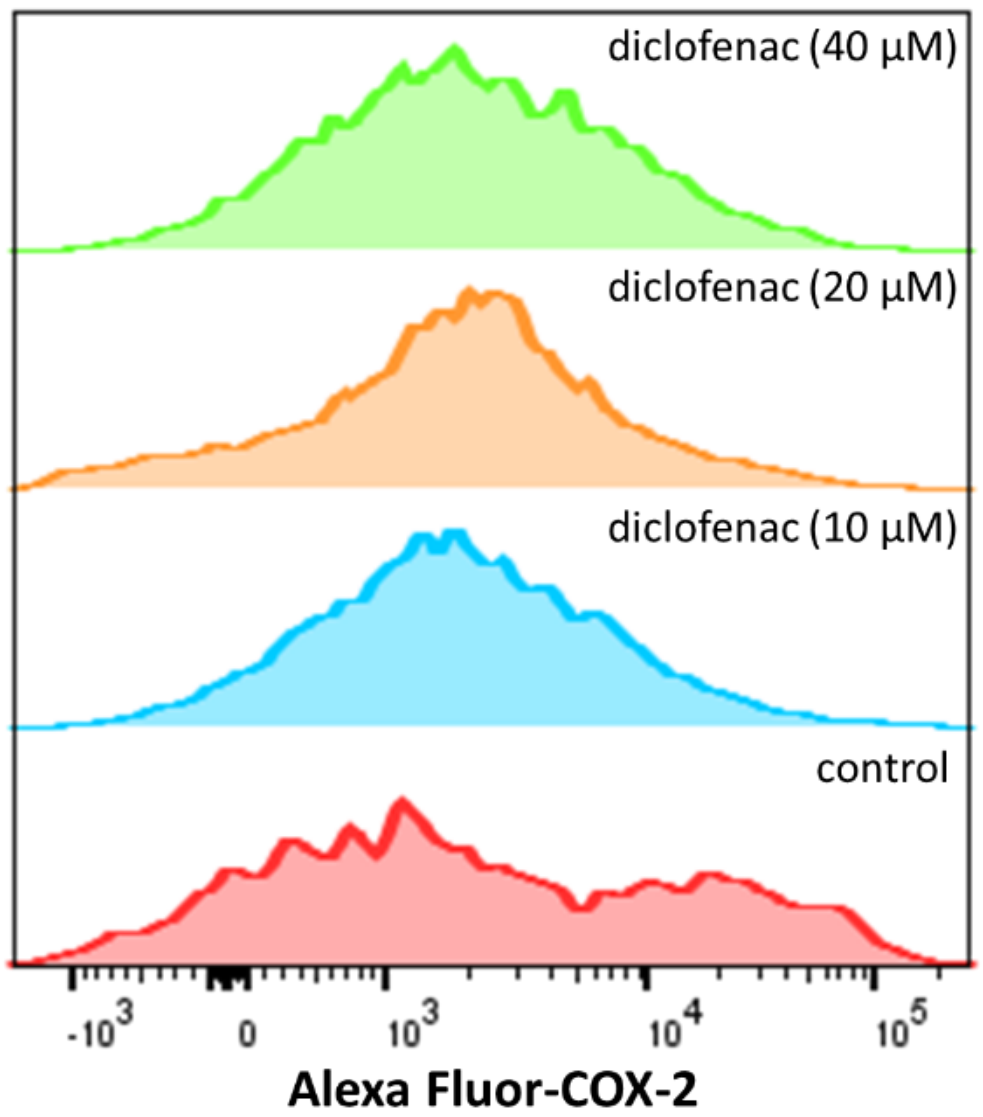

Fig. S47 Representative histograms displaying the green fluorescence emitted by anti-COX-2 Alexa Fluor 488 nm antibody-stained HMLER-shEcad cells treated with LPS (2.5 µg/ L) for 24 h (red) followed by 48 h in media containing diclofenac (10 µM, blue; 20 µM, orange; and 40 µM, green).

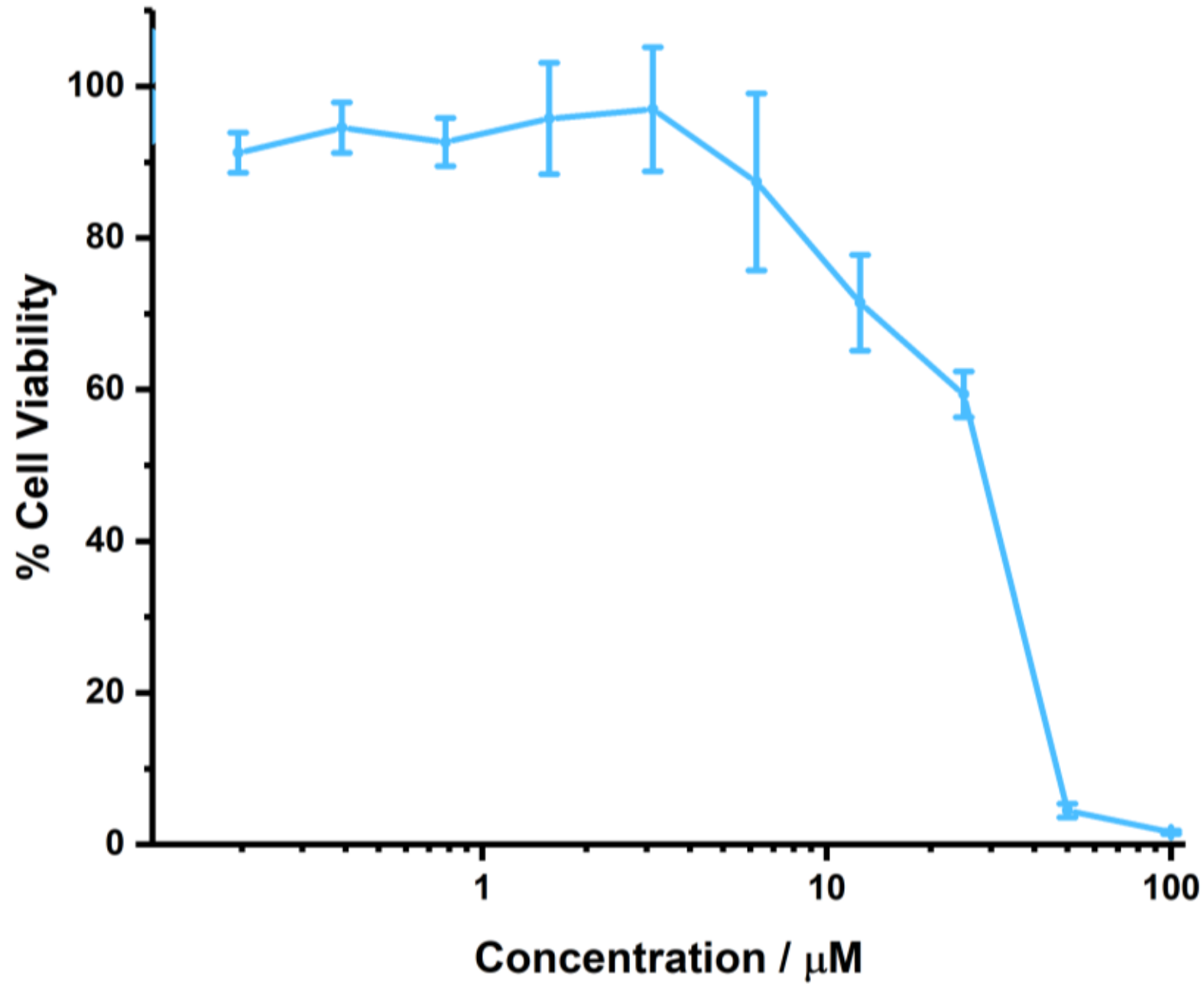

Fig. S48 Representative dose-response curve of **1** against HMLER-shEcad cells in the presence of PGE2 (20 µM) after 72 h incubation.

References.

1. P. Gerschel, K. Warm, E. R. Farquhar, U. Englert, M. L. Reback, D. Siegmund, K. Ray and U. P. Apfel, *Dalton Trans.*, 2019, **48**, 5923-5932.
2. G.M. Sheldrick, *Program for Area Detector Absorption Correction, Institute for Inorganic Chemistry*, University of Göttingen: Göttingen, Germany, 1996.
3. G.M. Sheldrick, *Acta Crystallogr. Sect. A*, 2008, **64**, 112-122.
4. G.M. Sheldrick, *Acta Crystallogr. Sect. C*, 2015, **71**, 3-8.
5. O.V. Dolomanov, L. J. Bourhis, R. J. Gildea, J. A. K. Howard, H. Puschmann, *J. Appl. Crystallogr.*, 2009, **42**, 339-341.
6. L. J. Farrugia, *J. Appl. Crystallogr.*, 2012, **45**, 849-854.
7. *POV-Ray*, Persistence of Vision Raytracer Pty. Ltd.: Williamstown, Australia, 2013.
